# LUMPAC 2.0—Bridging Theory and Experiment in the Study of Luminescent Systems

**DOI:** 10.1002/jcc.70143

**Published:** 2025-06-23

**Authors:** José Diogo Lisboa Dutra, Willyan Farias Oliveira, Gustavo Santana Silva, Thiago Dias Bispo, Ricardo Oliveira Freire

**Affiliations:** ^1^ Pople Computational Chemistry Laboratory, Department of Chemistry Federal University of Sergipe São Cristóvão Brazil; ^2^ Systems Development Office Federal Institute of Sergipe Aracaju Brazil

**Keywords:** energy transfer, lanthanide spectroscopy, lanthanides, luminescence, LUMPAC

## Abstract

There is no doubt about the relevance that lanthanide‐based luminescent systems have gained in recent decades. These systems have applications in various technological fields, and the development of these materials has been of great scientific interest since the 1970s. We made the first version of the LUMPAC software available 10 years ago. In our publication, we highlighted the scarcity of studies concerning the use of theoretical computational methods to design new luminescent systems and to explain laboratory‐observed phenomena involving such systems. In this work, we present the LUMPAC 2.0 software. We show how the first version contributed to increasing the number of studies involving theoretical computational methods, and we evaluate its use since its launch. This second version introduces highly relevant features and implementations and will undoubtedly be an important tool in new studies that will be carried out over the next few years.

## Graphical abstract

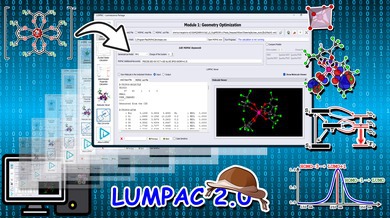

## 1 Introduction

Due to the wide applicability of lanthanide‐based luminescent systems in various technological fields, these systems are being studied by several research groups around the world. However, the use of theoretical computational techniques to aid experimental research only began in the early 1990s, with the development of the first quantum mechanics methods [1, 2].

During the 1990s, the few articles published concerning theoretical studies focused on developing methodologies, mostly involving researchers who developed and implemented these methodologies in theoretical or theoretical/experimental studies [3, 4, 5]. In other words, the use of these theoretical methods was mostly limited to their developers. The main reason is that these methodologies involve very complex equations and, for the most part, were not implemented using user‐friendly software.

**FIGURE 1:**
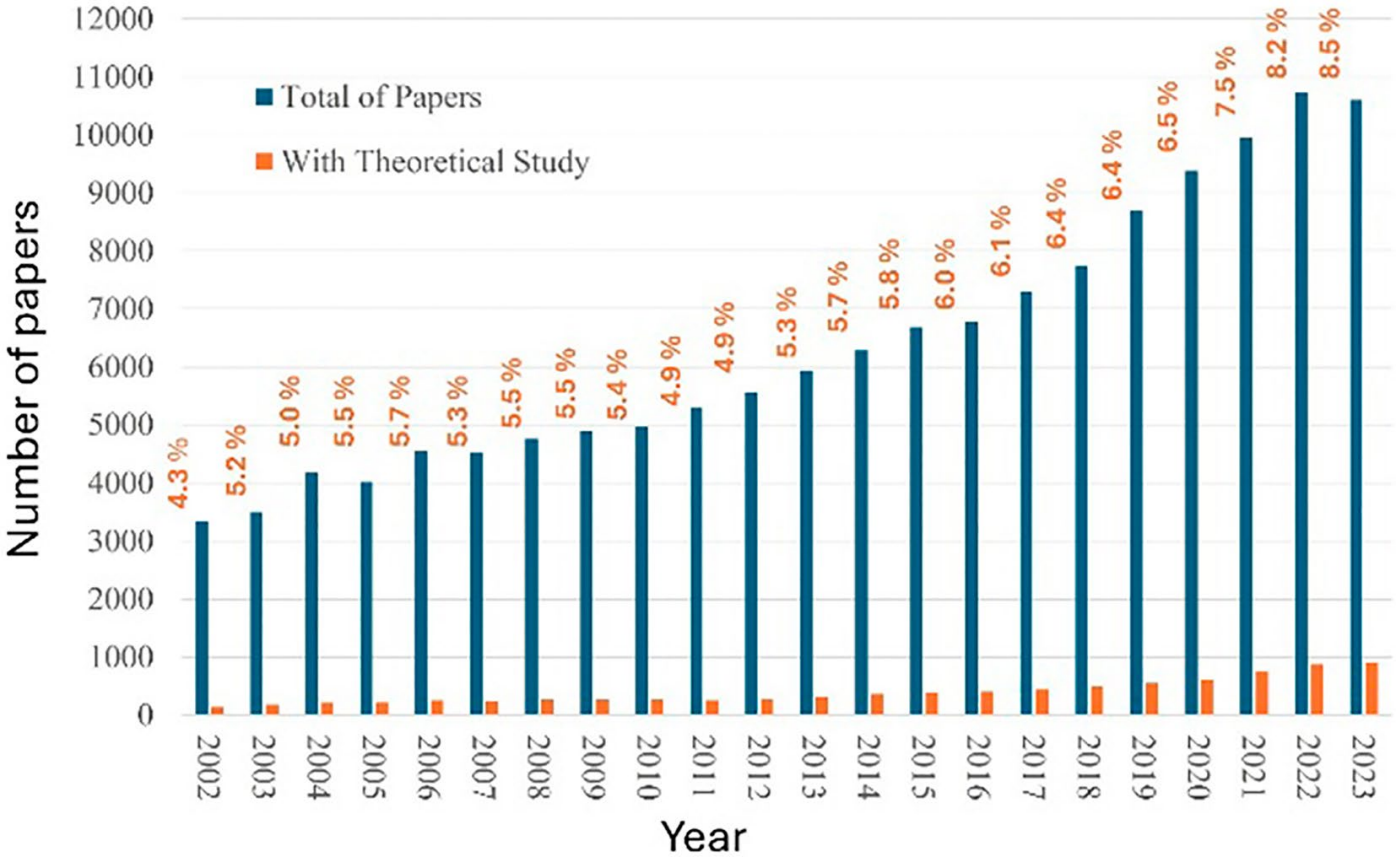
Numbers of papers published in the last decade containing the keywords “Lanthanide OR Rare Earth OR Europium” (in blue) and these keywords together with the keywords “Theoretical OR Calculation” (in orange).

Since the 2000s, there has been greater adoption of theoretical methods for studying systems involving lanthanide ions. Figure 1 shows the numbers of papers published in the last 20 years containing the keywords “Lanthanide OR Rare Earth OR Europium” (in blue) and the numbers of papers containing these keywords together with the keywords “Theoretical OR Calculation” (in orange). From 2002 to 2012, the percentage of works containing the term “Theoretical OR Calculation” ranged between 4.3% and 5.7%.

We developed the LUMPAC program to increase the adoption of theoretical methods by experimental researchers [6]. The first version was released to the scientific community in November 2013, on the website www.lumpac.pro.br. The software was developed to be efficient and user‐friendly, especially to meet the needs of experimental researchers who have limited time to specialize in the fields of theoretical chemistry and physics.

**FIGURE 2:**
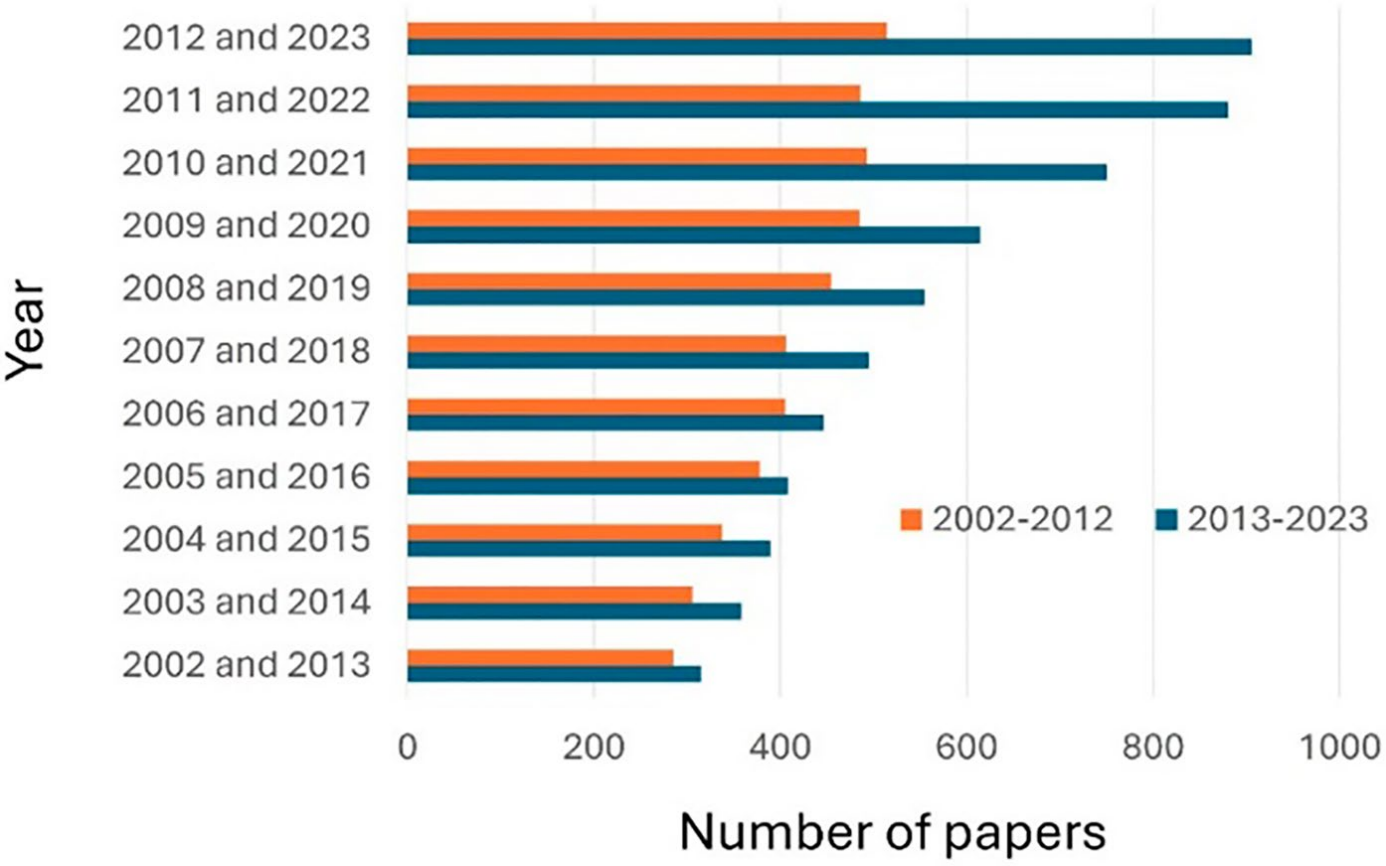
Comparison of the numbers of published papers containing the keywords “Lanthanide OR Rare Earth OR Europium” (in blue) and “Theoretical OR Calculation” (in orange), in sequential decades from 2002 to 2023.

Figure 2 illustrates the growing adoption of theoretical methodologies by presenting a year‐by‐year comparison of the number of works containing the keywords “Theoretical OR Calculation” across sequential decades. There has been a clear year‐by‐year increase in the number of works incorporating theoretical methodologies, suggesting further increases in the next years.

**FIGURE 3:**
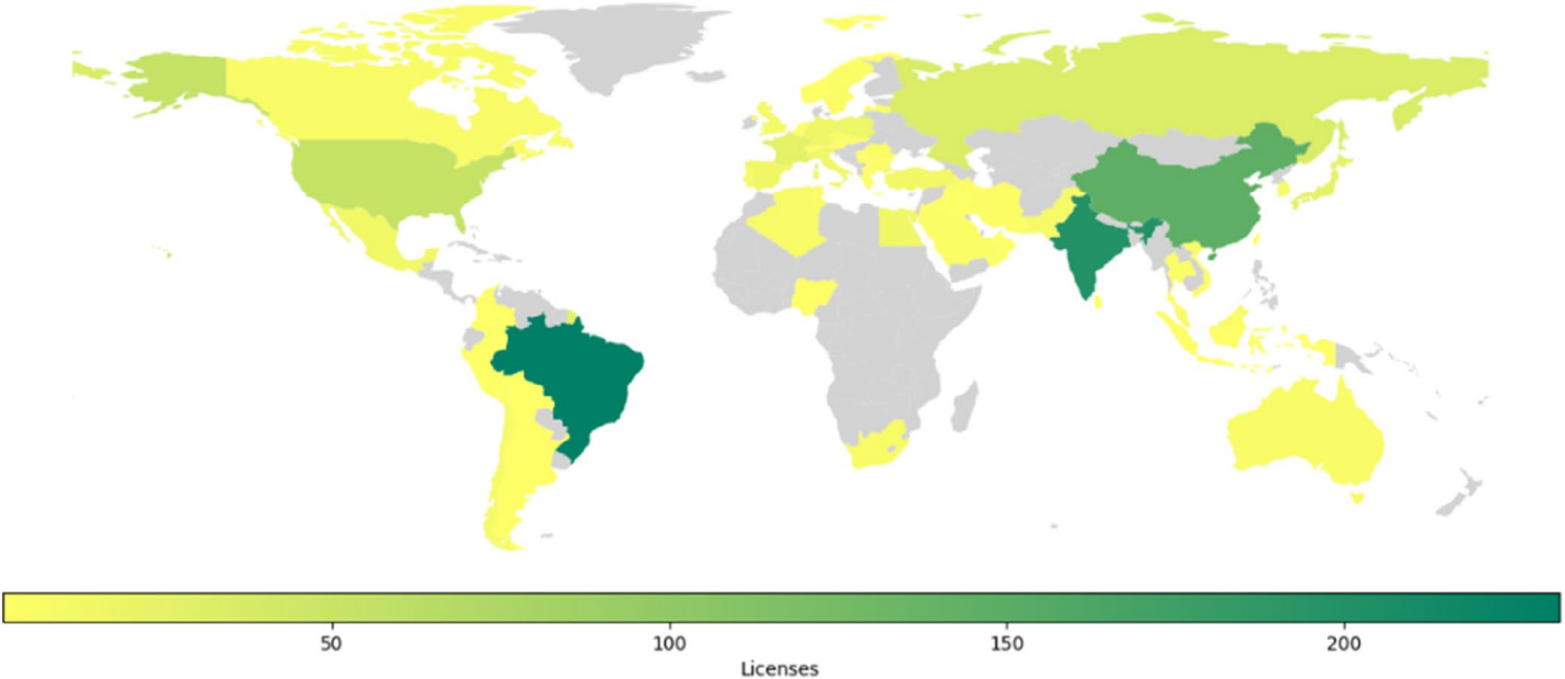
Current global distribution of passwords for the LUMPAC program (data collected on 25/04/2024).

The analysis showed that LUMPAC has been a success, with over 1000 passwords having been distributed across 60 different countries. Although India, China, and Brazil accounted for over 60% of these licenses (Figure 3), there has been widespread global adoption of LUMPAC, with the United States and Russia accounting for 53 and 35 licenses, respectively.

It is evident that this increase has not been directly linked to the use of the LUMPAC program, since the publication has currently received 186 citations. However, the development of LUMPAC has stimulated the creation of other programs with similar applications, such as JOYSpectra [7], JOES [8], and JOEX [9].

JOYSpectra, developed by Moura et al. [7] and released in 2021, is a free web platform that utilizes theoretical and mathematical foundations similar to those employed in LUMPAC for calculating the luminescent properties of systems containing Ln^3+^ ions. Initially, platform users do not need their own computing resources, since calculations submitted to the platform are executed on two dedicated computers, with output files sent to the user by e‐mail. However, there is a requirement to provide external data such as the Cartesian coordinates of the coordination polyhedron, singlet and triplet excited state energies, and the distance between the energy donor and acceptor centers (*R*_L_). These steps require a certain level of computational power.

JOES (Judd‐Ofelt from Emission Spectra), developed in 2019 by Ćirić et al. [8], is free, open‐source, and user‐friendly software, written in Java. The program is limited to calculating Judd‐Ofelt intensity parameters and other quantities derived from the emission spectra of Eu^3+^‐containing systems, such as radiative decay rate, lifetime, quantum efficiency, and sensitization efficiency. In JOES, there is no provision for the determination of theoretical properties including ligand–metal energy transfer rates and emission quantum yield.

JOEX, developed by Ćirić et al. [9], is a platform that enables the calculation of intensity parameters from excitation spectrum data. This methodology is particularly valuable, since it enables the determination of intensity parameters for systems where emission spectrum data are unavailable. The JOEX methodology has been implemented in LUMPAC 2.0.

LUMPAC indirectly contributed to the development and use of these other programs and methodologies by the scientific community studying systems containing lanthanides. Since its launch, LUMPAC has been applied in studies concerning luminescent polymers [10, 11, 12], pesticide sensors [13], potential applications in displays [14, 15, 16, 17, 18, 19, 20, 21] OLEDs [22, 23, 24, 25, 26, 27], potential WLEDs [28, 29, 30, 31], ammunition and forensic markers [32, 33, 34] potential dyes in bioimaging and biological markers [35, 36, 37, 38, 39, 40, 41, 42] potential nanothermometers [43] thermosensors [44, 45] membranes with potential applications in skin disease treatment [46], freshness markers for food [47], potential lasers [48], and potential enantioselectors [49].

There are studies in which all the functionalities of LUMPAC have been applied. For instance, Santos et al. [50] used it for purposes ranging from calculation of the geometry of the ground state to calculation of all the photophysical properties in a study of the luminescent properties of thin films of metal–organic structures. Other studies have employed specific modules for geometry optimization (Module 1), ligand excited state calculations (Module 2), and photophysical property calculations (Module 3). LUMPAC has been described as an easy‐to‐use tool of great importance in the field of lanthanide spectroscopy and has been suggested for use in theoretical studies [51, 52, 53].

Considering the interest shown in the program, this work presents version 2.0 of LUMPAC. In this version, the applicability of the program becomes more generalized to enable the study of the luminescent properties of other lanthanides, including methods for systems containing trivalent terbium ions. Additional new features have been provided in this version of the program. The next section details each of these functionalities, followed by a case study.

## 2 Implementation

The LUMPAC 2.0 program is structured into five modules, one more than in the previous version. The first module is designed for the process of geometry optimization and acts as an interface for the MOPAC program [54]. Due to the wide variety of models available for calculating ground state geometry [55, 56, 57, 58, 59] in MOPAC, Module 1 was modified by adding the option of using different available models for geometry optimization, as well as the ability to compare the optimized geometry with the starting structure (such as a crystallographic structure). Using the root‐mean‐square deviation (RMSD) as a metric, Module 1 facilitates identification of the model that provides a structure closest to the starting structure. Furthermore, the molecule viewer contained in Module 1 has been completely revised, enabling users to save high‐resolution figures, highlight the coordination polyhedron, adjust atom colors, and isolate the central metal environment for visualization.

Module 2 is designed to calculate ligand‐centered excited states, assisted by the ORCA program [60]. Improvements have included optimizing the source code and fixing bugs, while the graphical interface of Module 2 remains unchanged.

Module 3 has four subdivisions, with all of them having received important improvements. Module 3.1 includes the implementation of the calculation of intensity parameters from the excitation spectrum, using the JOEX methodology. Module 3.2 now enables the calculation of theoretical intensity parameters for multiple systems in parallel. Module 3.3, where modeling of ligand–metal energy transfer employs the ORCA or Gaussian output file, has received the greatest number of new features, the most important being the calculation of energy transfer and backtransfer rates for systems containing the Tb^3+^ ion. Other improvements made in this submodule are as follows: (i) the possibility of calculating multiple systems in parallel; (ii) the inclusion of new energy transfer channels in the calculation of the theoretical emission quantum yield; (iii) the possibility of replacing typical values for some quantities employed in the Malta models [61, 62] for calculating ligand–metal energy transfer rates; (iv) characterization of the excited states of the ligands involved in the energy transfer process, in terms of electronic transitions; and (v) automatic plotting of the Jablonski diagram. Finally, Module 3.4, which is responsible for obtaining the theoretical absorption spectrum, now allows the plotting of multiple absorption spectra and characterization of the absorption bands based on the electronic transitions involving the calculated molecular orbitals.

Module 4 is one of the most useful new features of LUMPAC 2.0. The main function of the module is to enable visualization of the structures and molecular orbitals calculated using the ORCA program. This facilitates characterization of the excited states centered on the ligands involved in the energy transfer process, as well as interpretation of the theoretical absorption spectrum. All the features already mentioned for the structure viewer in Module 1 are also present in Module 4.

Module 5 is a utility module for creating input–input or output–input files and converting files. This module was included in the first version of LUMPAC, while in version 2.0, it enables the conversion of several files simultaneously. The new features focus primarily on generating input files for ORCA. A database has been inserted into LUMPAC containing several effective core potentials (ECPs) and basis functions of the Stuttgart/Cologne Group (https://www.tc.uni‐koeln.de/PP/clickpse.en.html) for lanthanide ions. This enables the creation of input files for calculations using, for example, the DFT and TD‐DFT approaches with ORCA.

Because LUMPAC 2.0 primarily relies on semiempirical quantum calculations (from MOPAC and ORCA) and its native methods, calculations are inherently fast. Even for systems with many atoms, they typically finish within a few minutes on a standard personal computer.

## 3 Case Study

**FIGURE 4:**
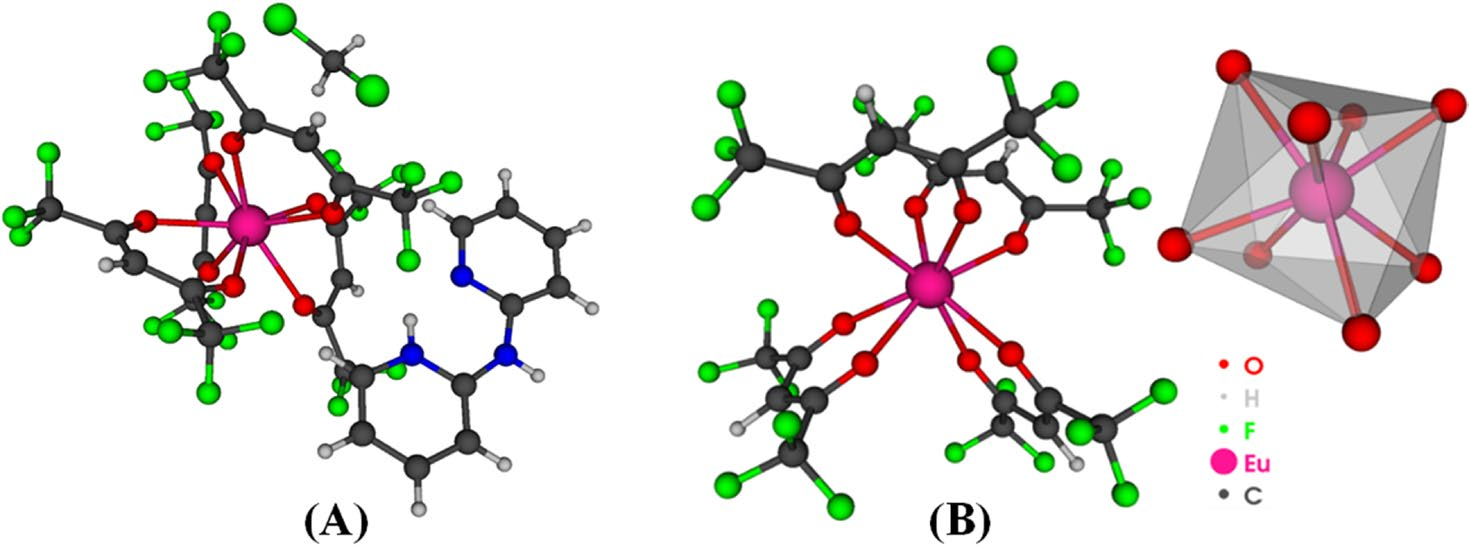
(A) Crystallographic structure of the [Eu(hfaa)_4_]^−^ complex (CSD code TEJSIO). (B) Structure of the complex modeled with the Sparkle/AM1 model, together with the corresponding coordination polyhedron.

A Eu^3+^ complex synthesized and spectroscopically characterized by Ilmi and collaborators [63] was chosen for use in a case study to illustrate the functionalities and potential of LUMPAC 2.0. The selected complex was tetrakis [Eu(hfaa)_4_]^−^, which contains the europium ion coordinated to four hfaa (hexafluroacetylacetonate) β‐diketonate ligands. Figure 4A shows the crystallographic structure of the complex, stabilized by both dichloromethane solvent molecules and the 2,2′‐dipyridylamine counterion. Figure 4 was generated using Module 4 in LUMPAC 2.0. The crystallographic structure and the experimental photophysical data were then used for comparison with the properties calculated using the theoretical approaches implemented in LUMPAC.

The crystallographic structure was used as input in the structural modeling employing different semiempirical models, implemented in the MOPAC 22.0.1 program [54]. Before performing the structural optimization, the solvent and counterion molecules were removed and a −1 charge was assigned to the complex, to compensate for the absence of the counterion. All the calculations were performed in parallel, using the new feature added to Module 1. At the end of the modeling, LUMPAC provided the root‐mean‐square deviation (RMSD) values (Table 1), using the experimental structure as a reference. Among the models evaluated, the Sparkle/AM1 model provided the lowest RMSD value, indicating that it best described the structure of the [Eu(hfaa)_4_]^−^ complex. Figure 4B illustrates the structure optimized with the Sparkle/AM1 model, highlighting the coordination polyhedron of the complex, with both graphical representations generated by the new LUMPAC structure viewer. The experimental Eu‐O bond distances ranged from 2.3595 to 2.4675 Å [63], while the theoretical calculations predicted a narrower range from 2.3944 to 2.3978 Å. Therefore, based on the RMSD results, the Sparkle/AM1 structure was chosen for further spectroscopic property calculations.

**TABLE 1:** RMSD calculated between the crystallographic structure of the [Eu(hfaa)_4_]^−^ complex and the optimized structures using different semiempirical models implemented using MOPAC in module 1 of LUMPAC 2.0.

RMSD (Å)						
Complex	RM1	Sparkle model				
		RM1	AM1	PM3	PM6	PM7
[Eu(hfaa)_4_]^−^	1.4443	1.3332	0.9271	1.0718	0.9763	1.2694

**FIGURE 5:**
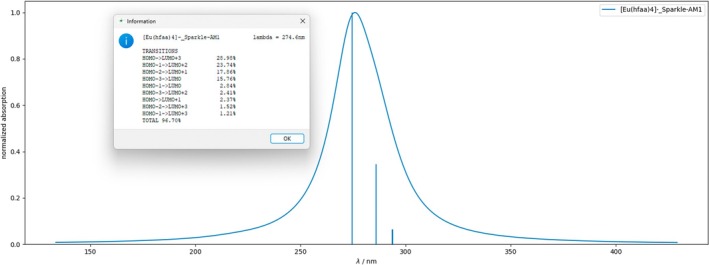
Theoretical absorption spectrum of the [Eu(hfaa)_4_]^−^ complex calculated with the INDO/S‐CIS model, using the Sparkle/AM1 geometry. The window shows the electronic transitions that significantly contribute to the corresponding singlet excited state.

Using Module 2, the ligand‐centered singlet and triplet excited states were calculated with the INDO/S‐CIS model [64, 65], implemented in the ORCA program [66]. In this procedure, the lanthanide ion was replaced by a +3e point charge. Figure 5 shows the absorption spectrum calculated using Module 3.4 of LUMPAC. This new feature represents the oscillator strengths in the form of narrow bars and enables spectral adjustment using Gaussian or Lorentzian functions. Clicking on the bars representing the intensity of the oscillator strength allows observation of the electronic transitions that most contribute to the composition of a given absorption band (Figure 5).

**FIGURE 6:**
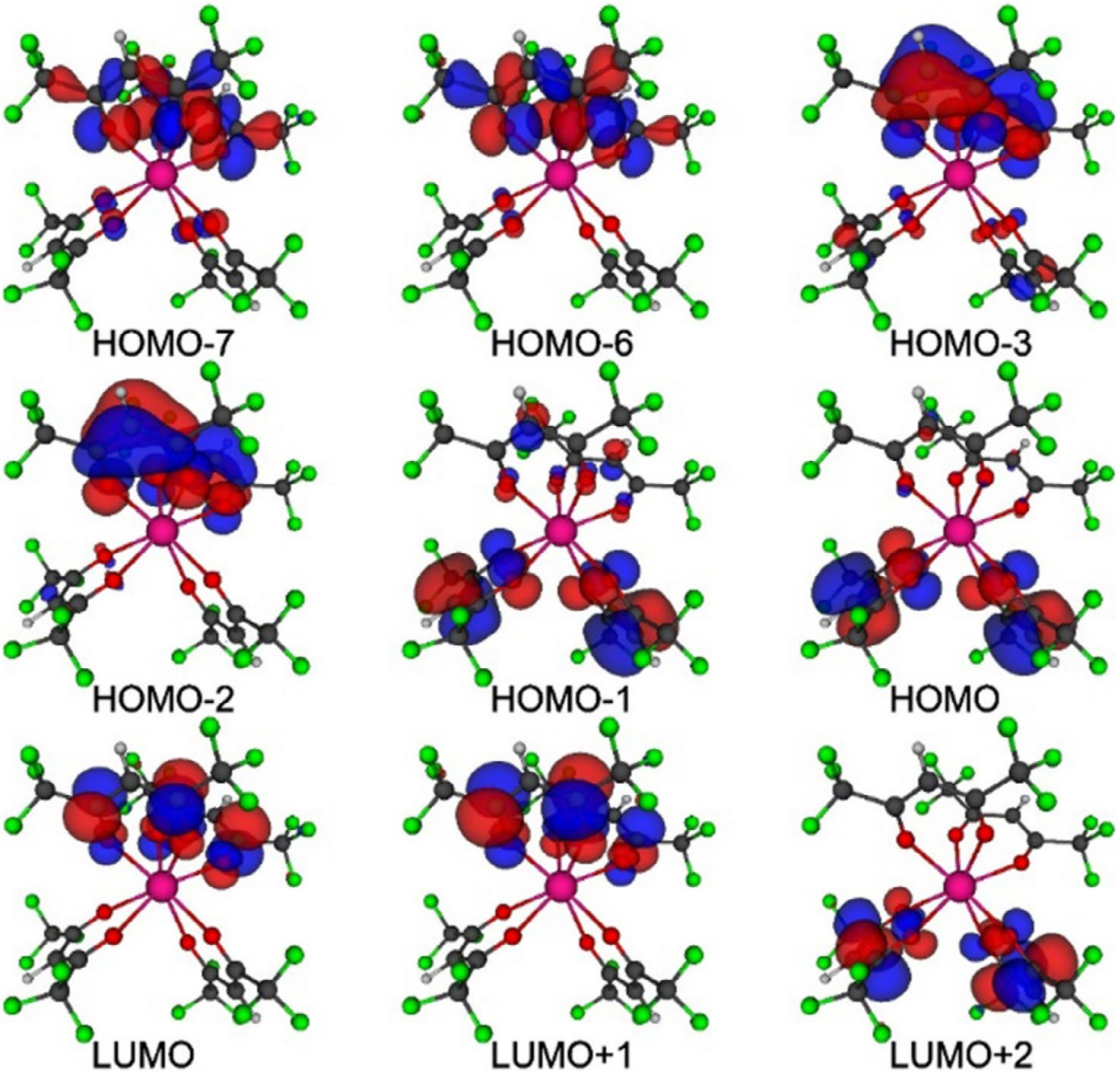
Selected molecular orbitals calculated using the INDO/S‐CIS model and generated by module 4.

The experimental absorption spectrum showed the presence of a broad absorption band in the 300 nm region. The absorption spectrum predicted by the INDO/S‐CIS model showed a maximum at 275.7 nm (Figure 5). The most significant electronic transitions contributing to this band were HOMO → LUMO+3, HOMO−1 → LUMO+2, HOMO−2 → LUMO+1, and HOMO−3 → LUMO, as indicated in the figure. LUMPAC also provided the percentage contribution of each electronic transition. Module 4 of LUMPAC enabled visualization and acquisition of graphical representation images of the molecular orbitals calculated with the INDO/S‐CIS model, using ORCA (Figure 6). Since the molecular orbitals involved in the main transitions had π character, the most intense absorption band was attributed to the π → π* transitions.

A crucial step in modeling the energy transfers of lanthanide complexes is the theoretical calculation of the Judd‐Ofelt intensity parameters (Ω_λ_). This procedure calculates the contribution of the electric dipole term to Ω_λ_ (Ω_λ_^FED^). Consequently, the rate of energy transfer due to the multipolar mechanism can then be determined [67]. The experimental Judd‐Ofelt parameters reported by Ilmi and coworkers were determined from the emission spectrum of the complex. These values served as a reference for the fitting process using the *QDC* model implemented in Module 3.2 [68]. Module 3.3 of LUMPAC was subsequently employed to calculate the theoretical radiative (*A*_rad_) and non‐radiative (*A*_nrad_) emission rates, together with the quantum efficiency (*η*) and the emission quantum yield (*q*) of the complex. Notably, the experimental lifetime was used to quantify *A*_nrad_. These calculated photophysical data are summarized in Table 2.

**TABLE 2:** Experimental and theoretical photophysical data for the [Eu(hfaa)_4_]^−^ complex.

[Eu(hfaa)_4_]^−^	Ω_2_ (Ω_2_^FED^)	Ω_4_ (Ω_4_^FED^)	Ω_6_ (Ω_6_^FED^)	*A*_rad_	*A*_nrad_	*τ*_obs_	*η*	*q*
	× 10^−20^ cm^2^			s^−1^		μs	%	
Experimental	22.02	6.93	—	844.35	145.75	1010	85.28	60.00
Theoretical	22.01 (0.0063)	6.94 (0.0334)	0.27 (0.0894)	815.15	174.94	—	82.33	82.01^a^, 62.56^b^

^a^ The value was calculated using the following default rates: S_1_ → S_0_ (10^6^ s^−1^), S_1_ → T_1_ (10^8^ s^−1^), and T_1_ → S_0_ (10^5^ s^−1^).

^b^ The value was calculated by changing the S_1_ → S_0_ rate from 10^6^ to 10^8^ s^−1^.

Table 3 summarizes the characterization of the lowest energy singlet (S_1_) and triplet (T_1_) states of the complex. Notably, the energies of the S_1_ and T_1_ levels, commonly observed for β‐diketonate ligands, facilitate the energy transfer in Eu^3+^ complexes. In addition, the small size of the β‐diketonate ligands results in a short distance between the energy donor and acceptor centers (*R*_L_), further enhancing the efficiency of the ligand–metal energy transfer. The main electronic transitions for S_1_ involved the HOMO−7, HOMO−6, LUMO, and LUMO+1 orbitals, while for T_1_, the orbitals with the greatest contributions were HOMO, HOMO−1, LUMO+2, and LUMO+3 (Table 3). The molecular orbitals involved in the composition of S_1_ were centered in the same region of the β‐diketonate ligands. This was also observed for T_1_, but involved ligands different from those for S_1_.

**TABLE 3:** Energy, *R*_L_, and electronic transitions with the greatest contributions for the lowest energy singlet and triplet excited states of the [Eu(hfaa)_4_]^−^ complex.

State	Energy (cm^−1^)	*R*_L_ (Å)	Greatest contributions	Total
S_1_	28240.20	3.3684	HOMO‐7 → LUMO (35.04%)	90.79%
			HOMO‐6 → LUMO (27.73%)	
			HOMO‐7 → LUMO+1 (15.84%)	
			HOMO‐6 → LUMO+1 (12.17%)	
T_1_	19333.80	3.3030	HOMO → LUMO+2 (33.49%)	92.90%
			HOMO‐1 → LUMO+2 (29.78%)	
			HOMO‐1 → LUMO+3 (18.12%)	
			HOMO → LUMO+3 (11.50%)	

The new version of LUMPAC allows selection of different transfer channels from the excitations of the ^7^F_0_ and ^7^F_1_ fundamental levels of the Eu^3+^ ion. Table 4 lists 120 rates considered in the modeling of the energy transfer of [Eu(hfaa)_4_]^−^, involving the following acceptor levels for Eu^3+^ available in LUMPAC 2.0: ^5^D_0_, ^5^D_1_, ^5^D_3_, ^5^D_4_, ^5^L_6_, ^5^L_7_, ^5^G_2_, ^5^G_3_, ^5^G_5_, and ^5^G_6_. The energy differences between these levels and the S_1_ and T_1_ states (Δ) showed that for T_1_, only the transfer channels involving the ^5^D_0_ and ^5^D_1_ states had energies lower than the donor level, favoring the energy transfer. This was corroborated by the energy transfer rates (*W*_ET_) obtained by the sum of the energy transfer rates of the multipolar (*W*_ET_^MM^) and exchange (*W*_ET_^EX^) mechanisms, which were greater than the corresponding energy backtransfer rates (*W*_BT_). Hence, the energy transfer efficiency was increased, since energy was directed to ^5^D_1_ and ^5^D_0_ (emitter state), making it difficult to lose energy in other routes. Other studies have found that the most efficient channels for the luminescence of Eu^3+^ compounds are T_1_ → ^5^D_0_ and T_1_ → ^5^D_1_ [69, 70, 71, 72, 73, 74]. As shown in Table 4, all the acceptor levels had energies lower than S_1_, and all the *W*_ET_ values were higher than the *W*_BT_ values for these channels, which also promoted the population of T_1_, due to its high *W*_BT_ with higher energy acceptor levels. These data were consistent with the quantum yield observed for [Eu(hfaa)_4_]^−^.

**TABLE 4:** Energy transfer (*W*_ET_) and backtransfer (*W*_BT_) rates for each transfer channel and corresponding energy difference between the energy donor and acceptor levels (Δ) for the [Eu(hfaa)_4_]^−^ complex.

Channel		Δ (cm^−1^)	*W*_ET_^MM^ (s^−1^)	*W*_ET_^EX^ (s^−1^)	*W*_ET_ (s^−1^)	*W*_BT_ (s^−1^)
Donor	Acceptor					
T_1_	^7^F_0_ → ^5^D_0_	2040.8	5.42 × 10^1^	0.00	5.42 × 10^1^	3.04 × 10^−3^
	^7^F_0_ → ^5^D_1_	306.8	0.00	1.71 × 10^9^	1.71 × 10^9^	3.92 × 10^8^
	^7^F_0_ → ^5^L_6_	−5991.2	5.59 × 10^−1^	0.00	5.59 × 10^−1^	1.68 × 10^12^
	^7^F_0_ → ^5^G_6_	−7418.2	3.85 × 10^−2^	0.00	3.85 × 10^−2^	1.09 × 10^14^
	^7^F_0_ → ^5^D_4_	−8252.2	2.10 × 10^−1^	0.00	2.10 × 10^−1^	3.24 × 10^16^
	^7^F_1_ → ^5^D_0_	2412.8	0.00	7.76 × 10^9^	7.76 × 10^9^	7.32 × 10^4^
	^7^F_1_ → ^5^D_1_	678.8	3.71 × 10^3^	8.84 × 10^5^	8.87 × 10^5^	3.42 × 10^4^
	^7^F_1_ → ^5^D_2_	−1777.2	0.00	7.24 × 10^7^	7.24 × 10^7^	3.64 × 10^11^
	^7^F_1_ → ^5^D_3_	−4649.2	6.67 × 10^1^	0.00	6.67 × 10^1^	3.22 × 10^11^
	^7^F_1_ → ^5^L_6_	−5619.2	2.27 × 10^−1^	0.00	2.27 × 10^−1^	1.15 × 10^11^
	^7^F_1_ → ^5^L_7_	−6651.2	1.71 × 10^−1^	0.00	1.71 × 10^−1^	1.22 × 10^13^
	^7^F_1_ → ^5^G_2_	−6686.2	0.00	1.32 × 10^7^	1.32 × 10^7^	1.12 × 10^21^
	^7^F_1_ → ^5^G_3_	−6916.2	6.26	0.00	6.26	1.59 × 10^15^
	^7^F_1_ → ^5^G_6_	−7046.2	3.05 × 10^−2^	0.00	3.05 × 10^−2^	1.45 × 10^13^
	^7^F_1_ → ^5^G_5_	−7057.2	1.94 × 10^−1^	0.00	1.94 × 10^−1^	9.69 × 10^13^
S_1_	^7^F_0_ → ^5^D_0_	10947.2	7.02 × 10^3^	0.00	7.02 × 10^3^	1.11 × 10^−19^
	^7^F_0_ → ^5^D_1_	9213.2	0.00	5.27 × 10^6^	5.27 × 10^6^	3.40 × 10^−13^
	^7^F_0_ → ^5^L_6_	2915.2	7.77 × 10^5^	0.00	7.77 × 10^5^	6.58 × 10^−1^
	^7^F_0_ → ^5^G_6_	1488.2	2.84 × 10^5^	0.00	2.84 × 10^5^	2.26 × 10^2^
	^7^F_0_ → ^5^D_4_	654.2	4.23 × 10^6^	0.00	4.23 × 10^6^	1.84 × 10^5^
	^7^F_1_ → ^5^D_0_	11319.2	0.00	2.05 × 10^6^	2.05 × 10^6^	5.43 × 10^−18^
	^7^F_1_ → ^5^D_1_	9585.2	2.36 × 10^6^	1.77 × 10^3^	2.36 × 10^6^	2.57 × 10^−14^
	^7^F_1_ → ^5^D_2_	7129.2	0.00	2.56 × 10^6^	2.56 × 10^6^	3.62 × 10^−9^
	^7^F_1_ → ^5^D_3_	4257.2	2.14 × 10^7^	0.00	2.14 × 10^7^	2.91 × 10^−2^
	^7^F_1_ → ^5^L_6_	3287.2	2.05 × 10^5^	0.00	2.05 × 10^5^	2.91 × 10^−2^
	^7^F_1_ → ^5^L_7_	2255.2	5.14 × 10^5^	0.00	5.14 × 10^5^	1.03 × 10^1^
	^7^F_1_ → ^5^G_2_	2220.2	0.00	1.45 × 10^8^	1.45 × 10^8^	3.45 × 10^3^
	^7^F_1_ → ^5^G_3_	1990.2	2.84 × 10^7^	0.00	2.84 × 10^7^	2.03 × 10^3^
	^7^F_1_ → ^5^G_6_	1860.2	1.46 × 10^5^	0.00	1.46 × 10^5^	1.95 × 10^1^
	^7^F_1_ → ^5^G_5_	1849.2	9.53 × 10^5^	0.00	9.53 × 10^5^	1.34 × 10^2^

To calculate the theoretical emission quantum yield, the following default rates, implemented in LUMPAC, were used for the decays involving ligand‐centered states: S_1_ → S_0_ (10^6^ s^−1^), S_1_ → T_1_ (10^8^ s^−1^), and T_1_ → S_0_ (10^5^ s^−1^). However, the new version of LUMPAC provides a very user‐friendly interface whereby these rates can be changed, for example, to reproduce the experimental quantum yield (Table 2). Furthermore, this feature allows the inclusion of other ligand states, in addition to those considered by default (S_1_ and T_1_).

**FIGURE 7:**
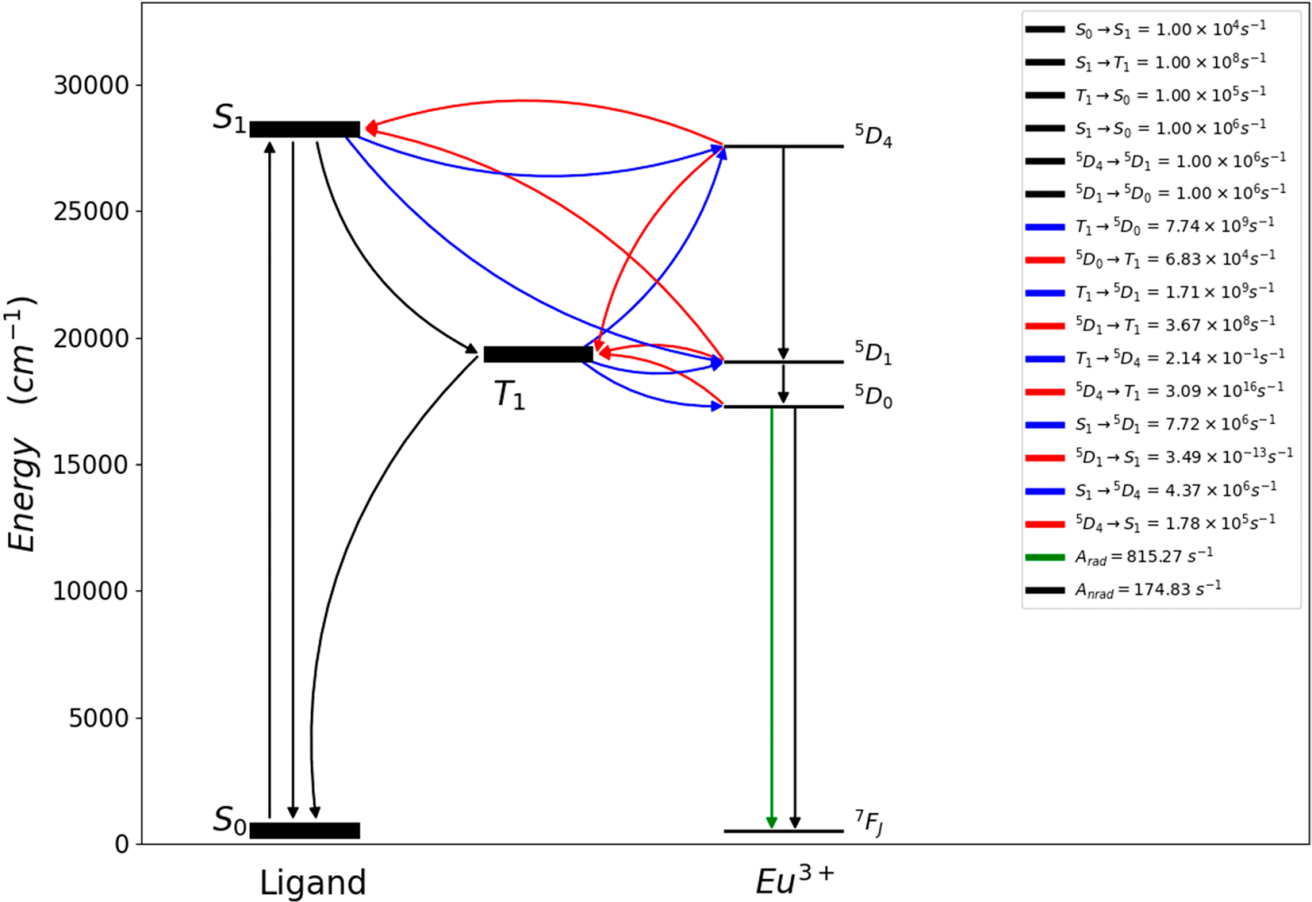
Simplified Jablonski diagram for the [Eu(hfaa)_4_]^−^ complex, obtained using LUMPAC 2.0.

The Jablonski diagram is commonly used to illustrate energy transfer channels. Figure 7 shows a representative example provided by LUMPAC 2.0, where the diagram illustrates the channels and ligand–metal energy transfer rates for the [Eu(hfaa)_4_]^−^ complex.

## 4 Conclusions

A decade ago, with the goal of promoting research concerning lanthanide ions and their luminescent properties, the LUMPAC 1.0 software was made available. Its use by various research groups worldwide, in a broad range of scientific areas, motivated the development of the new version (LUMPAC 2.0). This new version maintains the efficiency and user‐friendly interface of the first version, while incorporating several new features.

LUMPAC 2.0 introduces several new features, most notably: embedded RMSD calculations; the ability to perform parallel calculations on multiple systems; more sophisticated configurations for studying energy transfer channels; automatically generated Jablonski diagrams; and generation of three‐dimensional images of complexes and molecular orbitals. Importantly, all tools were integrated while preserving the layout of the first version, facilitating their learning process. Despite the change in the program's language, calculations remain as quick and efficient as in the first version.

Building on the success of LUMPAC 1.0 within the scientific community, LUMPAC 2.0 is expected to not only accelerate the adoption of theoretical methods by experimental groups, but also to stimulate discoveries in the years to come.

## Conflicts of Interest

The authors declare no conflicts of interest.

## Supporting information


**Data S1**Supporting Information.

## Data Availability

Data sharing not applicable to this article as no datasets were generated or analysed during the current study.
