# Supplementary material for: LUMPAC 2.0—Bridging Theory and Experiment in the Study of Luminescent Systems
Source: J Comput Chem. 2025 Jun 23;46(17):e70143. doi: 10.1002/jcc.70143 (PMC12183675; doi:10.1002/jcc.70143)
Supplement: Supplementary file 1 — Data S1Supporting Information. [file JCC-46-0-s001.docx]

**[LUMPAC](http://lumpac.pro.br)**

**Luminescence Package**

**User’s Manual**

**Version 2.0**

**By**

**José Diogo L. Dutra, Willyan F. Oliveira, Gustavo S. Silva,**

**Thiago D. Bispo, and Ricardo O. Freire**

**Pople Computational Chemistry Laboratory**

**Department of Chemistry**

**Federal University of Sergipe**

**São Cristóvão, SE, Brazil**

[**https://lumpac.pro.br**](https://lumpac.pro.br/)

**March 19^th^, 2025**

**Contents**

[Links to short YouTube videos highlighting the new features of LUMPAC 2.0 3](#_Toc197610325)

[Module 1 – Geometry Optimization 4](#_Toc197610326)

[Procedure for Geometry Optimization using LUMPAC 6](#_Toc197610327)

[Module 2 – Calculation of Excited States 12](#_Toc197610328)

[Procedure for Calculating Excited States using LUMPAC 13](#_Toc197610329)

[Module 3 – Calculation of Spectroscopic Properties 20](#_Toc197610330)

[Calculation of Experimental Intensity Parameters 22](#_Toc197610331)

[Procedure for Calculating Intensity Parameters and Radiative Emission Rate with LUMPAC 23](#_Toc197610332)

[Theoretical Calculation of Intensity Parameters 26](#_Toc197610333)

[Procedure for Calculation of the Theoretical Intensity Parameters using LUMPAC 27](#_Toc197610334)

[Calculation of Energy Transfer Rate and Emission Quantum Yield 32](#_Toc197610335)

[Procedure for Calculating Energy Transfer Rates and Emission Quantum Yield using LUMPAC 32](#_Toc197610336)

[Theoretical Calculation of the Absorption Spectrum 42](#_Toc197610337)

[Procedure for Calculation of the Theoretical Absorption Spectrum using LUMPAC 43](#_Toc197610338)

[Module 4 – Molecule Viewer 45](#_Toc197610339)

[Module 5 – File Converter 49](#_Toc197610340)

[Module 6 – About LUMPAC 53](#_Toc197610341)

Links to short YouTube videos highlighting the new features of LUMPAC 2.0

| **Module #1 and #2** | <https://youtu.be/6B0FZ7jrAbY> |
| --- | --- |
| **Module #3** | <https://youtu.be/CIIKxtwfg1U> |
| **Module #4** | <https://youtu.be/OVzgU8PCIRU> |
| **Module #5** | <https://youtu.be/-jlGJ5RFsoI> |

**Manual for the Computational Software Package LUMPAC – Version 2.0**

Module 1 – Geometry Optimization

Geometry optimization is the first step in studying the luminescence of a system containing a lanthanide ion. The geometry is crucial for the theoretical prediction of the Judd-Ofelt parameters, as these parameters depend on the chemical environment surrounding the lanthanide ion (first coordination sphere). To this end, LUMPAC features a module that serves as a graphical interface for the semiempirical package MOPAC (**Figure 1**). This module further simplifies the application of semiempirical models developed by our research group,^[1–6]^ which are implemented in MOPAC.


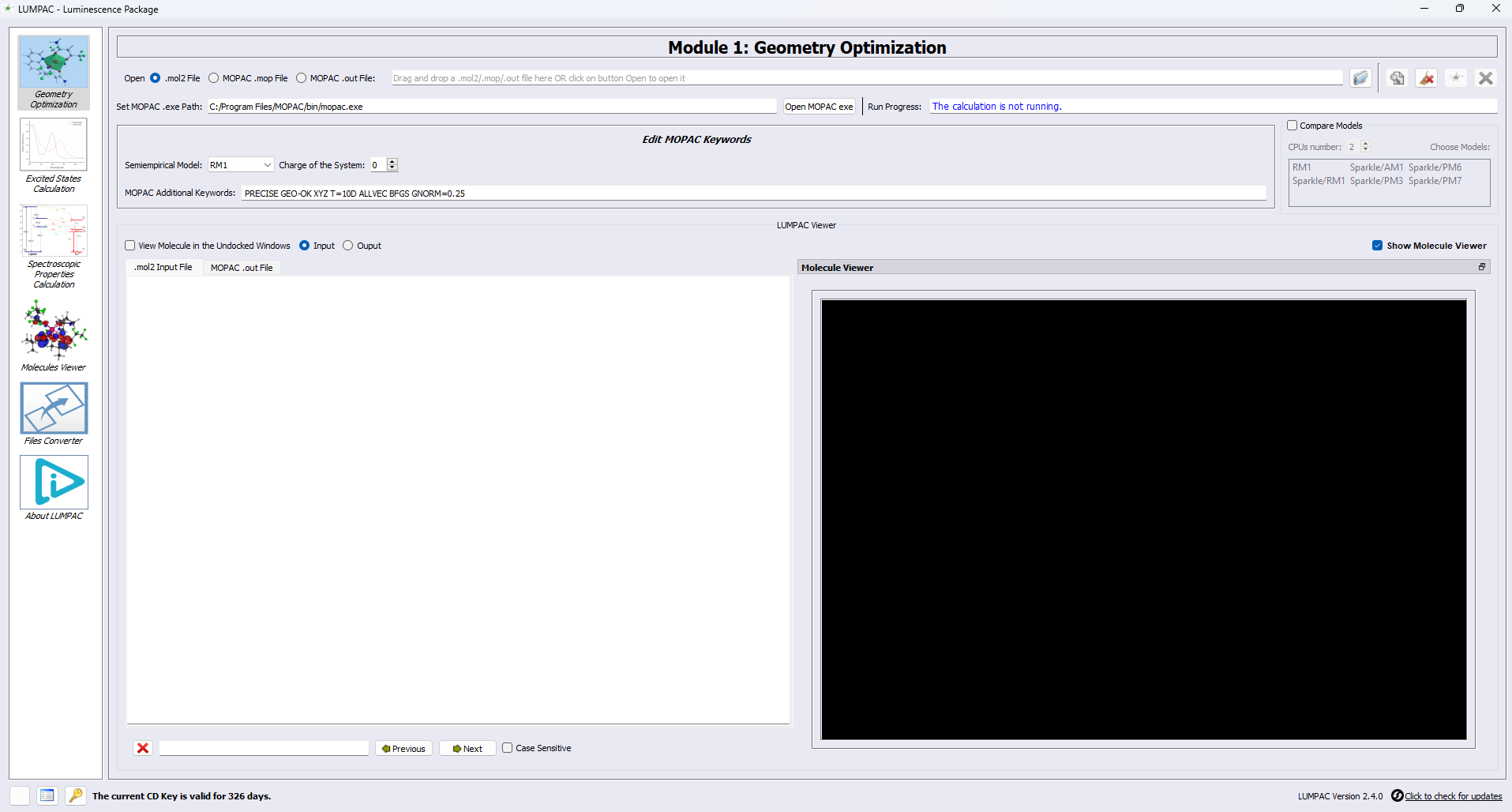


**Figure 1**. Module responsible for the geometry optimization using the semiempirical models included in the MOPAC package.

All features implemented in LUMPAC 2.0 will be demonstrated using the [Eu(btfa)_3_(bpy)] complex (**Figure 2**) as a case study, where btfa ligand is β-diketone 4,4,4-trifluoro-1-phenyl-1,3-butanedione, while bpy stands for bipyridine.^[7]^

**Figure 2**. Two-dimensional representation of the [Eu(btfa)3(bpy)] complex.

A file with a .mol2 extension containing the geometry of the compound is required for the geometry optimization. The .mol2 file (“Tripos Mol2 file”) is a file format that contains atomic positions in Cartesian coordinates and information about the bonds connecting the atoms. **Figure 3** shows the .mol2 file of the [Eu(btfa)_3_(bpy)] complex, created using the Mercury program from the corresponding crystallographic structure.

| 1 | @<TRIPOS>MOLECULE | | | | | | | | | | | | | |
| --- | --- | --- | --- | --- | --- | --- | --- | --- | --- | --- | --- | --- | --- | --- |
| 2 | ***** | | | | | | | | | | | | | |
| 3 | 84 92 0 0 0 | | | | | | | | | | | | | |
| 4 | SMALL | | | | | | | | | | | | | |
| 5 | GASTEIGER | | | | | | | | | | | | | |
| 6 |  | | | | | | | | | | | | | |
| 7 | @<TRIPOS>ATOM | | | | | | | | | | | | | |
| 8 | 1 | Eu1 | 2.1491 | | 13.6285 | | 4.3851 | Eu | 1 | RES11 | | 0.0000 | | |
| 9 | 2 | F1 | -1.9750 | | 11.9825 | | 2.2022 | F | 1 | RES11 | | 0.0000 | | |
| 10 | 3 | F2 | -1.5794 | | 10.3949 | | 3.5416 | F | 1 | RES11 | | 0.0000 | | |
| 11 | 4 | F3 | -1.3002 | | 10.1168 | | 1.4498 | F | 1 | RES11 | | 0.0000 | | |
| 12 | 5 | F4 | 3.9520 | | 18.6738 | | 2.8922 | F | 1 | RES11 | | 0.0000 | | |
| 13 | 6 | F5 | 3.9550 | | 17.4180 | | 1.2010 | F | 1 | RES11 | | 0.0000 | | |
| 14 | 7 | F6 | 5.1691 | | 16.9410 | | 2.8952 | F | 1 | RES11 | | 0.0000 | | |
| 15 | 8 | F7 | -1.8535 | | 14.3589 | | 7.2105 | F | 1 | RES11 | | 0.0000 | | |
| 16 | 9 | F8 | -0.9042 | | 14.5110 | | 9.1019 | F | 1 | RES11 | | 0.0000 | | |
| 17 | 10 | F9 | -0.9429 | | 12.6569 | | 8.1076 | F | 1 | RES11 | | 0.0000 | | |
| 18 | 11 | O1 | 2.9953 | | 12.2862 | | 2.6219 | O.3 | 1 | RES11 | | 0.0000 | | |
| ... |  | | | | | | | | | | | | | |
| 89 | 82 | H39 | 7.5656 | | 14.2822 | | 4.2780 | H | 1 | RES11 | | 0.0000 | | |
| 90 | 83 | C40 | 5.5662 | | 13.9281 | | 4.4866 | C.3 | 1 | RES11 | | 0.0000 | | |
| 91 | 84 | H40 | 5.2995 | | 14.7674 | | 4.1307 | H | 1 | RES11 | | 0.0000 | | |
| 92 | @<TRIPOS>BOND | | | | | | | | | | | | | |
| 93 | 1 | 1 | 11 | 1 | |  | | | |  | | |  | |
| 94 | 2 | 1 | 12 | 1 | |  | | | |  | | |  |  |
| 95 | 3 | 1 | 13 | 1 | |  | | | |  | | |  |  |
| ... |  | | | | | | | | | | | | | |
| 182 | 90 | 81 | 82 | 1 | |  | | | | |  | | |  |
| 183 | 91 | 81 | 83 | 0 | |  | | | | |  | | |  |
| 184 | 92 | 83 | 84 | 1 | |  | | | | |  | | |  |
| 185 |  | | | | | | | | | | | | | |

**Figure 3**. .mol2 file of the [Eu(btfa)_3_(bpy)] system created using the Mercury program.

An input file for MOPAC with a .mop extension is created based on the atomic connectivities present in the .mol2 file. The structure is organized to enable LUMPAC to easily identify the coordination polyhedron. The .mol2 file can be generated using graphical programs such as HyperChem,^[8]^ Gabedit,^[9]^ Avogadro,^[10]^ Mercury,^[11]^ and others. Additionally, programs like HyperChem, Gabedit, and Avogadro enable the interactive drawing of three-dimensional chemical structures. A detailed tutorial on how to build chemical structures using Gabedit and HyperChem can be accessed at the following link: <http://www.sparkle.pro.br/tutorial/drawing-complexes>.

Attention: It is crucial to ensure that, if the structure is manually constructed, all bonds involving the lanthanide ion and the donor atoms are explicitly represented, as shown in **Figure 4**.


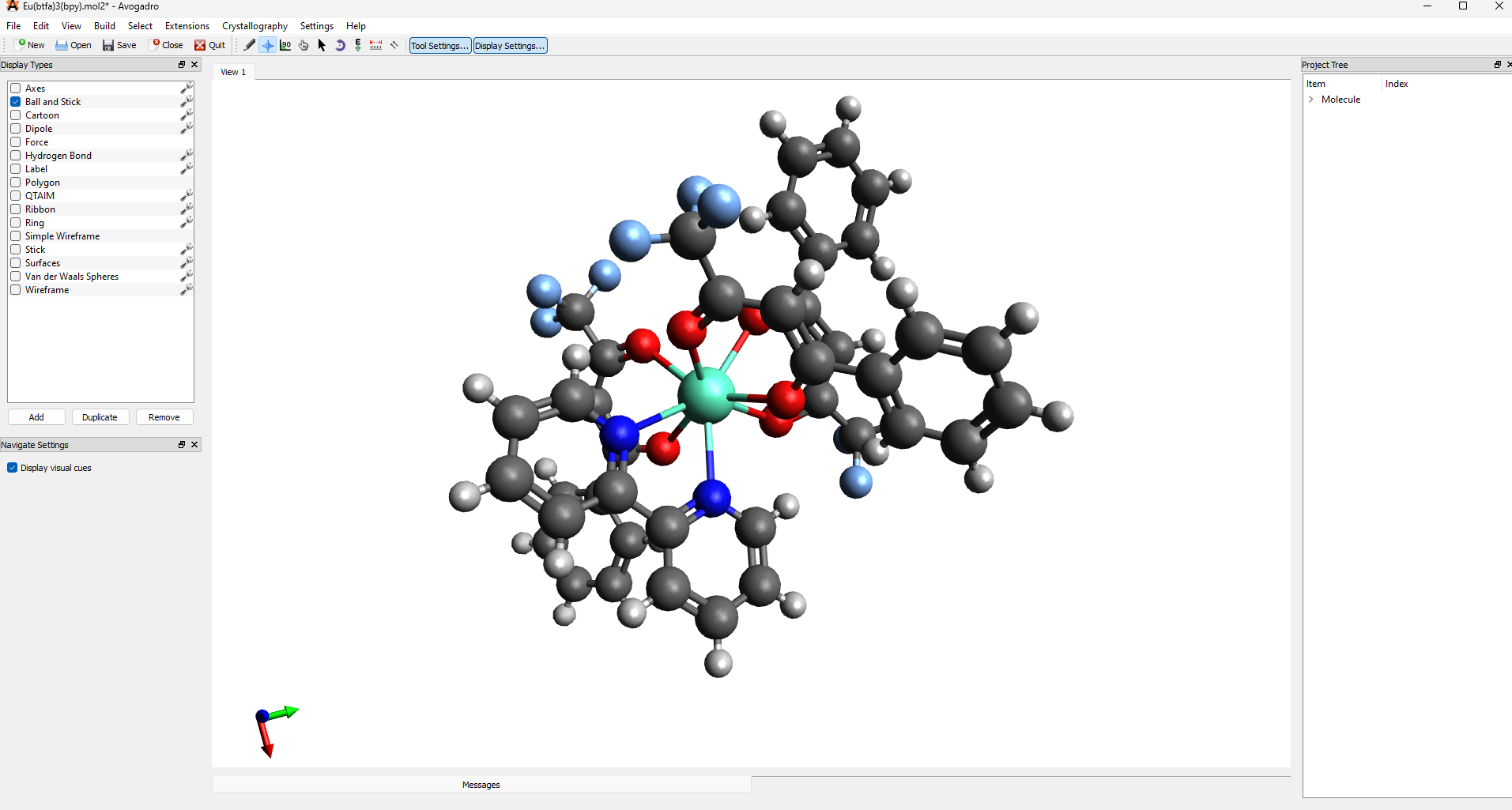


**Figure 4**. Structure of the [Eu(btfa)_3_(bpy)] complex constructed using the Avogadro program, explicitly showing all bonds between the lanthanide ion and the donor atoms of the ligands.

Procedure for Geometry Optimization using LUMPAC

1. *Ensure that the “Open .mol2 File” option is selected. Then, click on button
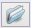
 (***Figure *5****) to open the .mol2 file.*

**Figure 5** provides a detailed overview of the functionalities of each graphical element in Module 1 of LUMPAC, emphasizing the different types of files that can be used as input files for LUMPAC.


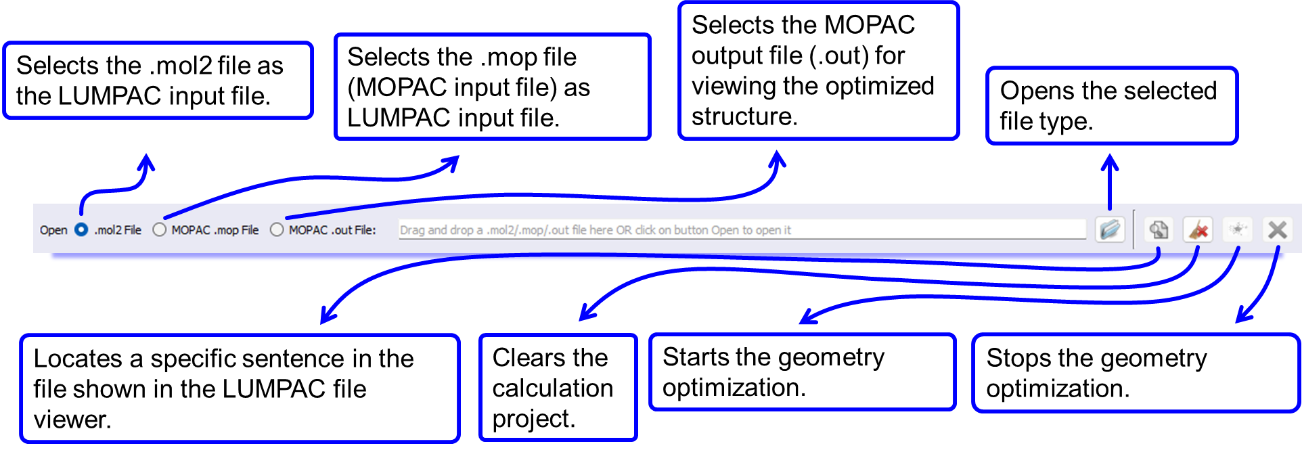


**Figure 5**. Different types of files can be used as input files for the geometry optimization using LUMPAC.

1. *Click on button
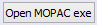
 (****Figure 6****) to define the directory of the external MOPAC program.*

Since 2022, the latest source code of MOPAC^[12]^ has been freely distributed through the following GitHub repository: <https://github.com/openmopac/mopac>. The executable version of MOPAC is installed without requiring an activation key. MOPAC includes all semiempirical models parameterized for the lanthanide ions, namely: RM1, Sparkle/AM1, Sparkle/PM3, Sparkle/PM6, Sparkle/PM7, and Sparkle/RM1. Integrating MOPAC into LUMPAC is straightforward, requiring only the provision of the directory where MOPAC is located (**Figure 6**).

**
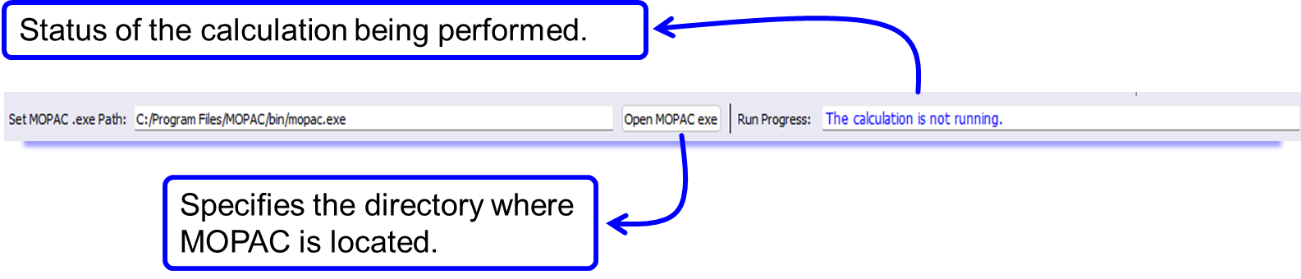
**

**Figure 6**. Procedure for integrating a MOPAC executable into LUMPAC.

1. *The keywords (***Figure *7****) must be appropriately specified before performing the geometry optimization. Once the .mol2 file is opened, the button
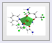
 (***Figure *5****) will be enabled.*

The LUMPAC interface (**Figure 7**) allows for the editing of keywords. The semiempirical model and the total charge of the system are the most important parameters to define. As users type, the edit line autocompletes the keywords. In this way, the edit line ensures that the user enters the keywords with the correct syntax.


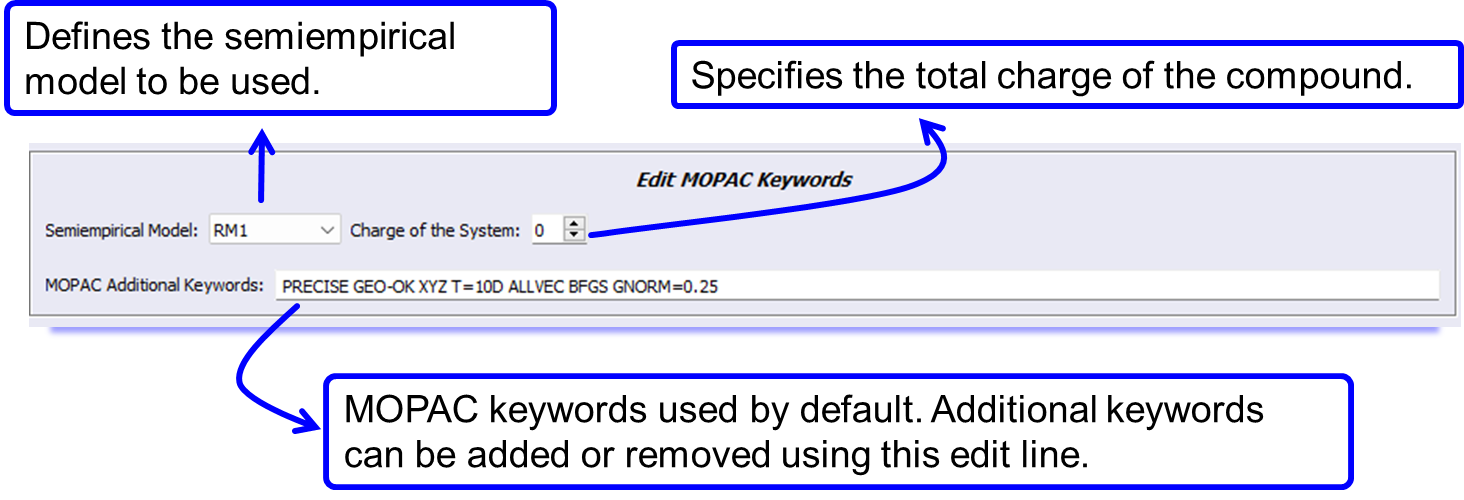


**Figure 7**. MOPAC keyword editor in the geometric optimization module.

1. *Select the Compare Models group box (***Figure *8****) to compare the geometry of the input file with the geometries calculated by the semiempirical models.*

The Compare Models group box allows for the estimation of the differences between the input geometry and the geometries calculated using different semiempirical models. This estimation is performed by superimposing the calculated structures onto the initial structure and using RMSD (root mean square deviation of atomic positions) to quantify errors in bond distances and angles. The resulting error values are saved in a text file named rmsd.txt (**Figure 9**), located in a folder called *compare_geoms* along with other calculation output files.


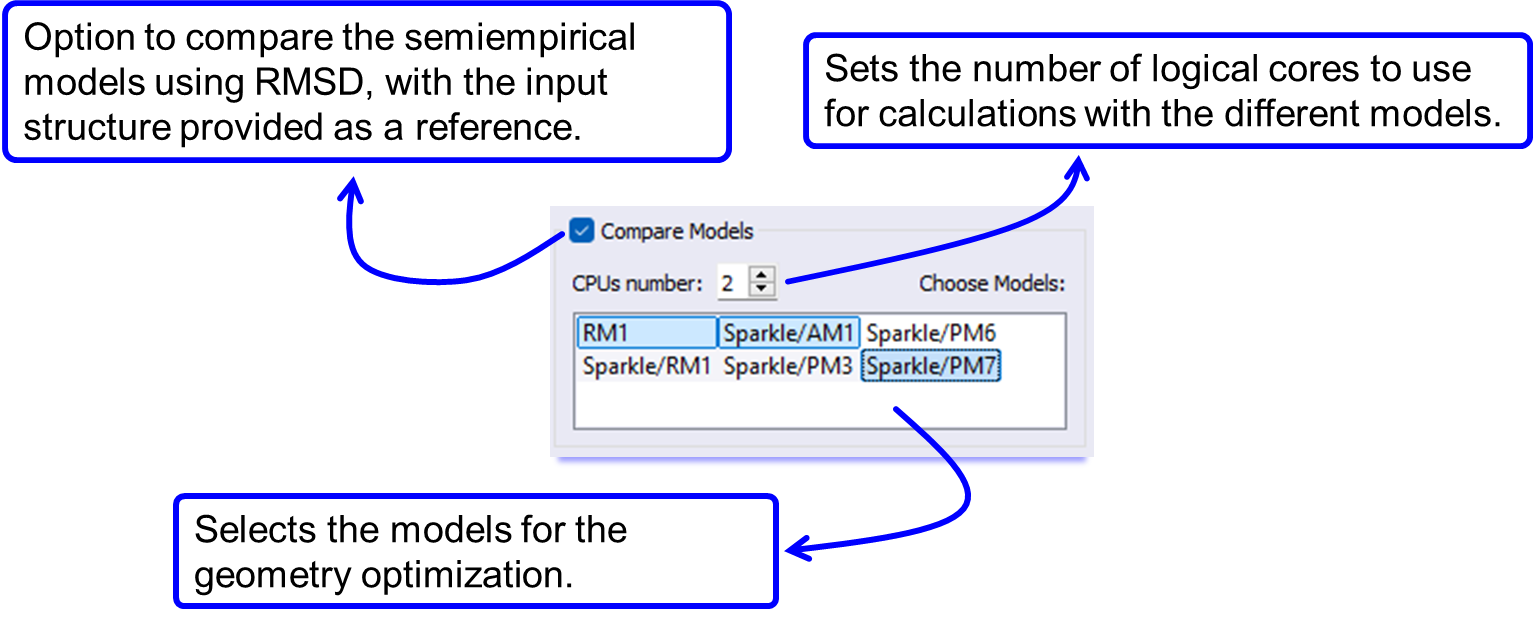


**Figure 8**. Selection of models for comparison and configuring the number of logical cores for parallel computation using the Compare Models feature.

| ... |  | | |
| --- | --- | --- | --- |
| 23 |  | Values of RMSD(angs.) | |
| 24 |  | normal | kabsch |
| 25 | Eu(btfa)3(bpy)_RM1 | 1.4880 | 1.4630 |
| 26 | Eu(btfa)3(bpy)_Sparkle-RM1 | 1.4747 | 1.4558 |
| 27 | Eu(btfa)3(bpy)_Sparkle-AM1 | 1.3336 | 1.3048 |
| 28 | Eu(btfa)3(bpy)_Sparkle-PM3 | 1.2959 | 1.2683 |
| 29 | Eu(btfa)3(bpy)_Sparkle-PM6 | 1.3218 | 1.2993 |
| 30 | Eu(btfa)3(bpy)_Sparkle-PM7 | 1.1727 | 1.1599 |

**Figure 9**. The rmsd.txt file contains the estimated errors between the input structure and the structures calculated using the selected semiempirical models.

1. *Click on button*
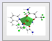
 *to execute the geometry optimization using the MOPAC program*.

Attention: The output file generated by MOPAC will have a .out extension and retains the same name as the input file. This output file will be saved in the same directory as the input file. As soon as the .out file is modified during the background execution of MOPAC, the LUMPAC file viewer updates its content to show the progress of the geometry optimization.

1. *After the calculation completes, the user can view either the initial or optimized structure (***Figure *10****).*

**Figure 10** displays the LUMPAC file and molecule viewers within the geometry optimization module. The molecule viewer can be highlighted to visualize the molecular structure in detail, modify visual parameters, and save the image as a .png file (**Figure 11**). Double-clicking the molecule viewer window returns it to its original position.


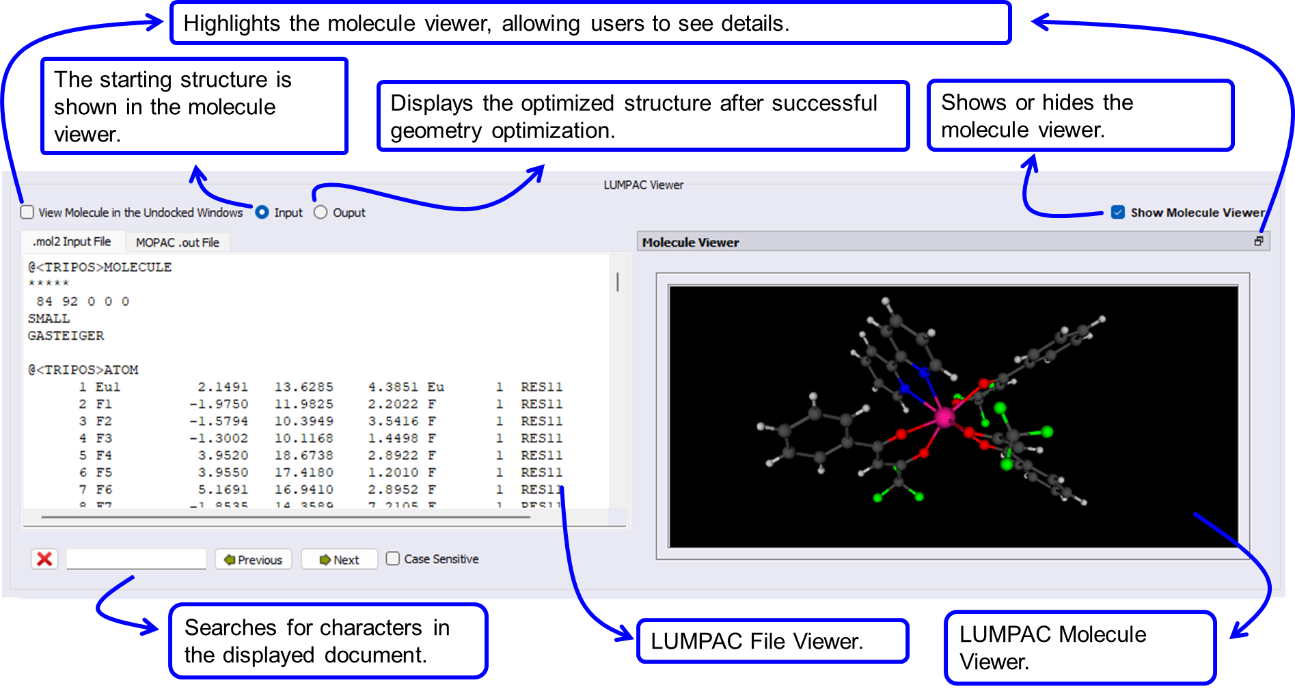


**Figure 10**. LUMPAC file and molecule viewers contained in the geometry optimization module.


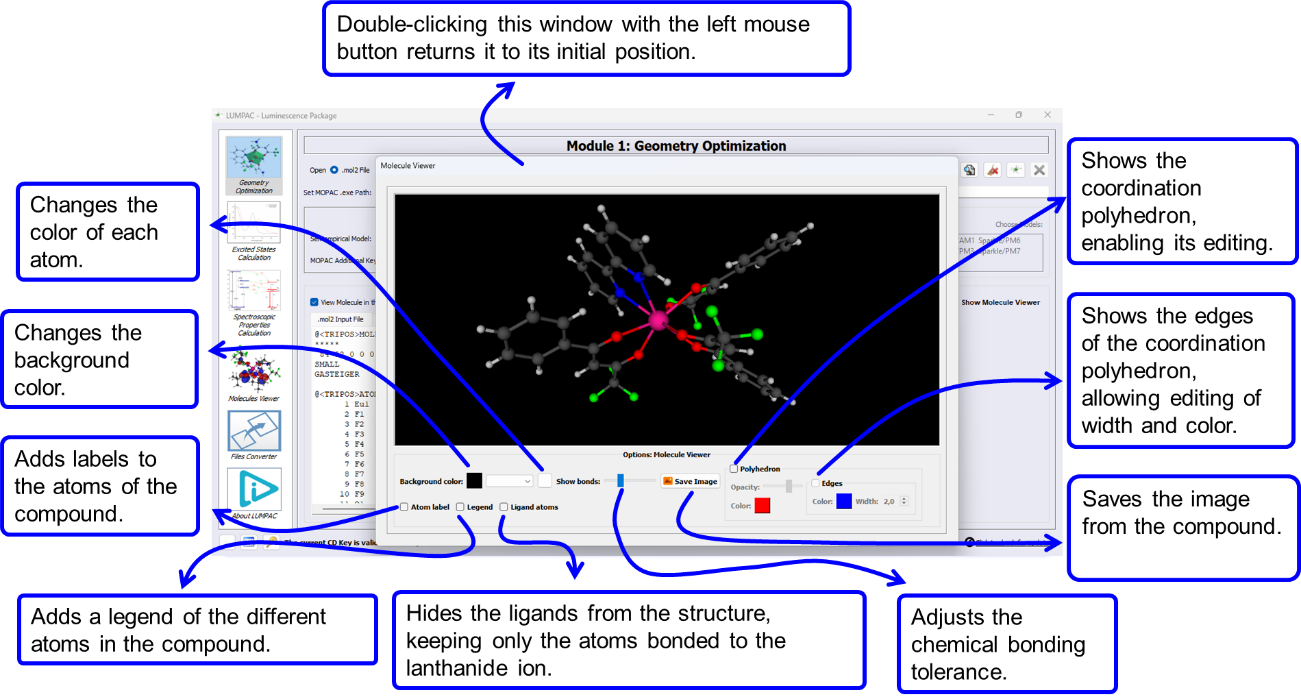


**Figure 11**. Visualization of the [Eu(btfa)_3_(bpy)] complex and options for editing the image.

**Table 1** lists the mouse cursor commands for translation, rotation, and zoom functions, which control visualization settings.

| **Table 1**. Translation, rotation, and zoom commands for the molecule viewer. | |
| --- | --- |
| **Command** | **Function** |
| Press the left mouse button and move the mouse. | The view is moved freely. |
| While holding SHIFT, press the left mouse button and move the mouse. | A translation transformation is performed. |
| Press the right mouse button and move the mouse. | Moving the mouse forward increases the zoom; moving it backward decreases the zoom. |
| Scroll the mouse wheel up or down. | The zoom is increased or decreased. |
| While holding CTRL, press the left mouse button and move the mouse. | A rotation transformation is applied. |

LUMPAC can perform geometry optimization with a .mop file (MOPAC input file), but MOPAC keywords editing is disabled within the LUMPAC interface. Therefore, users must edit the .mop file using a text editor.

Module 2 – Calculation of Excited States

Module 2 of LUMPAC acts as a graphical interface for the ORCA program,^[13]^ enabling the calculation of the singlet and triplet excited states of the ligands in the compound (**Figure 12**). The excited states energies are important for calculating the energy transfer and back-transfer rates between the ligands, which act as an antenna, and the lanthanide ion. The lanthanide ion is conveniently replaced by a 3e+ point charge to calculate the excited states of the ligands using the semiempirical INDO/S model (Intermediate Neglect of Differential Overlap),^[14,15]^ implemented in ORCA.


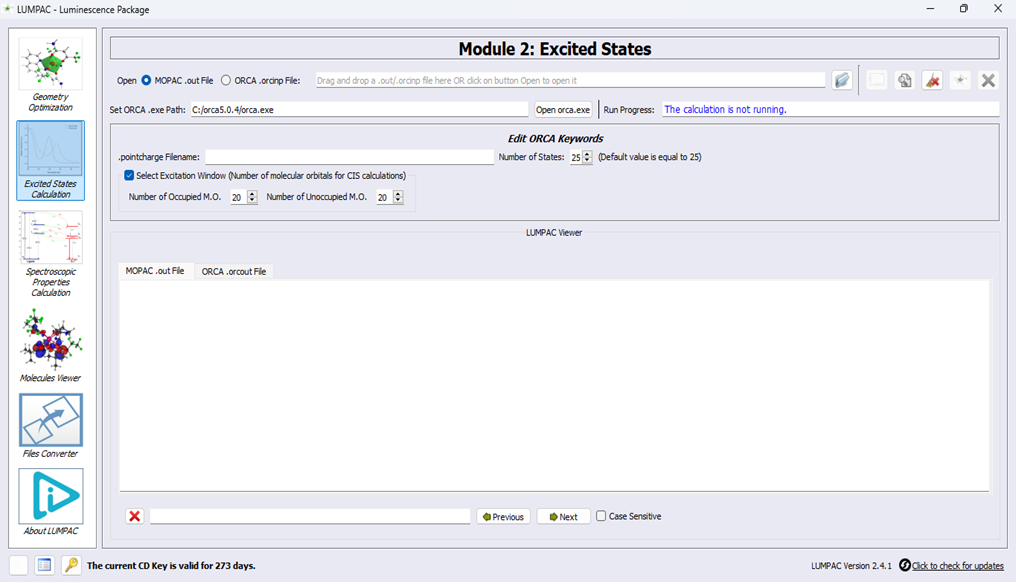


**Figure 12**. Module for calculating the excited states energies of the ligands using the ORCA program.

ORCA is a modern computational package for electronic structure calculations developed by Prof. Frank Neese (Universität Bonn). The ORCA development project benefits from contributions by numerous research groups and is made freely available to academics. Users can download the ORCA installer by registering on the website, <https://orcaforum.kofo.mpg.de/app.php/portal>, with no password required. Newer versions of ORCA increasingly require more storage space due to added features. For example, ORCA version 5.0.4 for Windows requires approximately 24 GB of storage space. The latest version, 6.0, includes an installer that allows users to select specific packages for installation. Regardless of the version, all files must be located within a single directory.

As LUMPAC uses command lines to execute ORCA, placing the ORCA executable files in a short-named directory (e.g., C:\ORCA) it is strongly recommended to prevent interaction issues.

The ORCA program can perform a wide variety of calculations, from geometry optimization to spectral parameters calculations, using various levels of theory. Despite this extensive functionality, LUMPAC uses ORCA only for calculating the excited states of ligands using the configuration interaction singles (CIS) method, applying the semiempirical INDO/S model. Because the ORCA program is not public domain like MOPAC, LUMPAC cannot distribute it. Therefore, users are required to obtain ORCA via the procedure described above.

Procedure for Calculating Excited States using LUMPAC

1. *Specify the directory of the external ORCA program (***Figure *13****), using the procedure previously demonstrated for the geometry optimization module.*
2. *After defining the directory containing the ORCA program, click on button
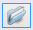
 (***Figure *13****) to open the MOPAC output file (.out) created by the geometry optimization described earlier.*


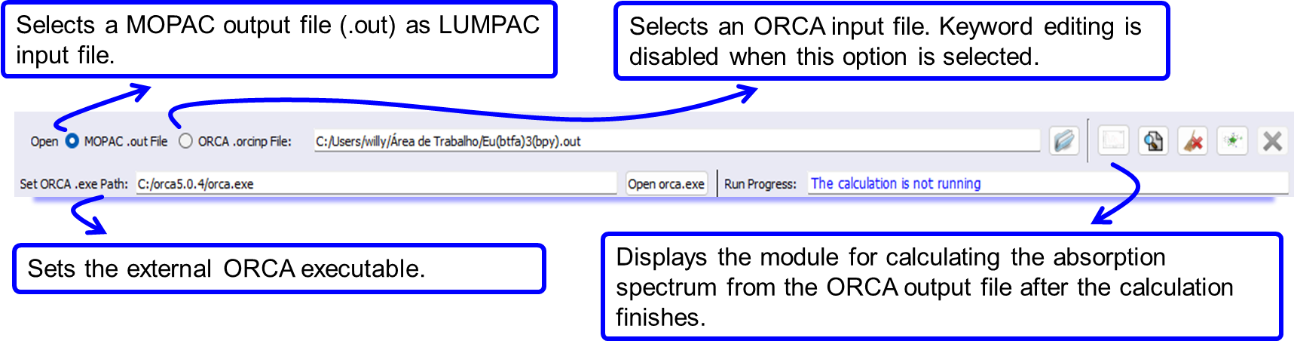


**Figure 13**. ORCA program integration in LUMPAC and the various input file formats supported for excited states calculations.

1. *Before executing the excited states calculations, specify the parameters for the ORCA calculation (***Figure *14****). Optionally, the number of excited states and the range of orbitals considered in the configuration interaction singles (CIS) can be modified. The button*
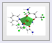
 *(***Figure *13****) will be enabled once the MOPAC output file is opened.*


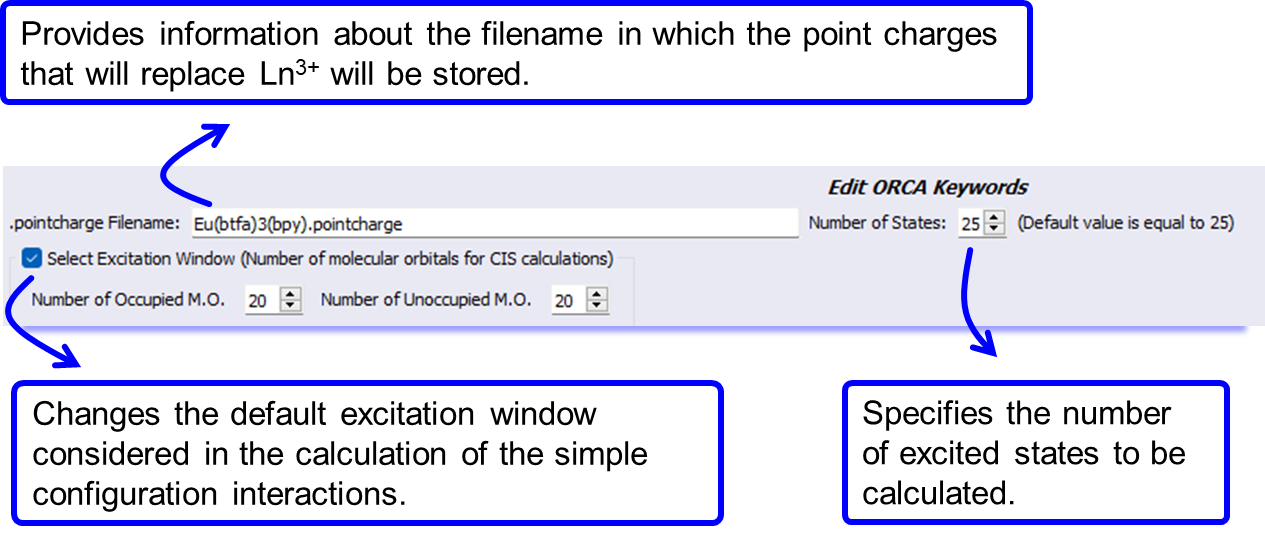


**Figure 14**. Editor for setting the parameters of the excited states calculations using ORCA.

Before LUMPAC executes ORCA, the ORCA input file is created with the same name as the corresponding MOPAC output file, but with the .orcinp extension. The ORCA input file created for the [Eu(btfa)_3_(bpy)] compound can be viewed in **Figure 15**, and the ORCA output file will have the .orcout extension.

| 1 | !RHF ZINDO/S TightSCF DIIS PrintBasis | | | | | | | | | |
| --- | --- | --- | --- | --- | --- | --- | --- | --- | --- | --- |
| 2 | %method | | | | | | | | | |
| 3 | frozencore fc_ewin | | | | | | | | | |
| 4 | End | | | | | | | | | |
| 5 | %cis | | | | | | | | | |
| 6 | ewin -0.415580,0.073982 | | | | | | | | | |
| 7 | nroots 25 | | | | | | | | | |
| 8 | maxdim 100 | | | | | | | | | |
| 9 | Triplets true | | | | | | | | | |
| 10 | end | | | | | | | | | |
| 11 | %output | | | | | | | | | |
| 12 | print[p_mos] 1 | | | | | | | | | |
| 13 | print[p_basis] 2 | | | | | | | | | |
| 14 | print[p_orben] 2 | | | | | | | | | |
| 15 | end | | | | | | | | | |
| 16 | %pointcharges "[Eu(btfa)3(bpy)].pointcharge" | | | | | | | | | |
| 17 | *xyz -3 1 | | | | | | | | | |
| 18 | O | 2.8953 | 12.4062 | | 2.8338 | |  |  |  |  |
| 19 | O | 0.6162 | 12.0218 | | 3.9834 | |  |  |  |  |
| 20 | O | 0.7133 | 15.0113 | | 3.6223 | |  |  |  |  |
| 21 | O | 3.3223 | 15.2461 | | 3.4113 | |  |  |  |  |
| … |  | | | | | | | | | |
| 98 | H | 7.8108 | 13.7665 | | 4.7531 | |  |  |  |  |
| 99 | C | 5.6575 | 13.5756 | | 4.8680 | |  |  |  |  |
| 100 | H | 5.4579 | 14.4262 | | 4.1831 | |  |  |  |  |
| 101 | * |  | |  | |  |  |  |  |  |

**Figure 15**. .orcinp file created by LUMPAC from the MOPAC output file of [Eu(btfa)_3_(bpy)] and used as the ORCA input file.

The number of excited states (nroots) to be calculated is specified in the seventh line of the .orcinp file (**Figure 15**). The eighth line displays the size of the Configuration Interaction (CI) matrix used in the CIS calculation. As shown in **Figure 15**, the sixteenth line indicates the filename for the .pointcharge file, which stores the point charges replacing the lanthanide. Therefore, the .orcinp file contains only the atomic coordinates of the organic ligands. Because the [Eu(btfa)_3_(bpy)] complex contains a single Eu^3+^ ion, only one point charge will be used (**Figure 16**).

| 1 | 1 |  |  |  |
| --- | --- | --- | --- | --- |
| 2 | 3.0 | 2.2392 | 13.6030 | 4.7796 |

**Figure 16**. .pointcharge file containing the +3e point charge used to replace the lanthanide ion.

Warning: The .pointcharge file provides the Cartesian coordinates of the lanthanide ion when the .orcout file is used as input for LUMPAC. Therefore, the .pointcharge file must be in the same directory and have the same name as the corresponding .orcout file.

The seventeenth line of the .orcinp file (**Figure 15**) specifies the coordinate type, compound charge, and multiplicity (considering only the ligands). A multiplicity of 1 (singlet) indicates paired electrons; therefore, the first line shows the RHF (Restricted Hartree-Fock) keyword, indicating a closed-shell calculation. ORCA will report an error and terminate the calculation if the charge is incorrect. To minimize errors, LUMPAC automatically transfers the charge from geometry optimization to the ORCA input file. Thus, users must verify the charge from defined in Module 1.

1. *The ORCA output file will have the same name as the .orcinp file, but with the .orcout extension.*

**Figure 17** shows the ORCA output file in the LUMPAC file viewer. The .orcout file can also be viewed with any text editor.


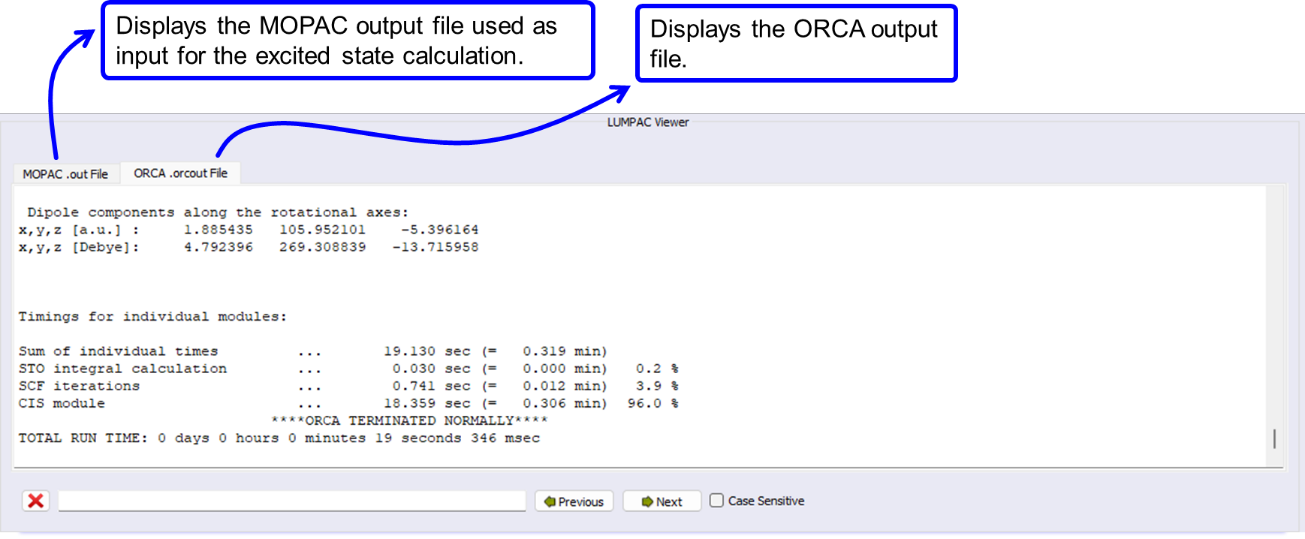


**Figure 17**. ORCA output file displayed in the LUMPAC file viewer.

LUMPAC enables excited states calculations using an existing ORCA input file (.orcinp). As LUMPAC does not provide editing capabilities for the .orcinp file keywords, users must utilize a text editor if modifications are required.

The “Select Excitation Window” option (**Figure 14**) allows users to customize the molecular orbital range considered in the CIS calculation. By default, LUMPAC uses a 20×20 excitation window, which includes the 20 highest energy occupied and the next 20 lowest energy unoccupied molecular orbitals.

**Figure 18** shows the section of the ORCA output file containing the energies of the molecular orbitals calculated for [Eu(btfa)_3_(bpy)]. The OCC column in **Figure 18** indicates the orbital occupation: 2.0000 for occupied and 0.0000 for unoccupied orbital. Thus, orbital 148 corresponds to the highest energy occupied orbital (HOMO), and orbital 149 is the lowest energy unoccupied orbital (LUMO).

| . . . 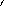 | | | | | |
| --- | --- | --- | --- | --- | --- |
| ---------------- | | | | | |
| ORBITAL ENERGIES | | | | | |
| ---------------- | | | | | |
|  | | | | | |
| NO | OCC | E(Eh) | E(eV) |  |  |
| 0 | 2.0000 | -1.880755 | -51.1780 |  |  |
| 1 | 2.0000 | -1.879220 | -51.1362 |  |  |
| 2 | 2.0000 | -1.878338 | -51.1122 |  |  |
| . . . |  |  |  |  |  |
| **129** | **2.0000** | **-0.415580** | **-11.3085** |  |  |
| 130 | 2.0000 | -0.415056 | -11.2942 |  |  |
| 131 | 2.0000 | -0.406435 | -11.0597 |  |  |
| . . . |  |  |  |  |  |
| 147 | 2.0000 | -0.322395 | -8.7728 |  |  |
| 148 | 2.0000 | -0.315355 | -8.5812 |  |  |
| 149 | 0.0000 | -0.045314 | -1.2330 |  |  |
| 150 | 0.0000 | -0.028167 | -0.7665 |  |  |
| . . . |  |  |  |  |  |
| **168** | **0.0000** | **0.073982** | **2.0131** |  |  |
| 169 | 0.0000 | 0.079583 | 2.1656 |  |  |
| 170 | 0.0000 | 0.086871 | 2.3639 |  |  |
| . . . |  |  |  |  |  |

**Figure 18**. Section of the .orcout output file displaying the energies of the molecular orbitals used to select the orbital range will be used in the CIS calculation.

A single-point SCF (Self-Consistent Field) calculation determines the orbital energies before a CIS calculation. **Figure 18** illustrates the orbital window used in the CIS calculation for the case study: orbitals 129 (-0.415580 Eh) and 168 (0.073982 Eh), highlighted in bold, correspond to the lower and upper limits of the excitation window, respectively. The sixth line of **Figure 15** demonstrates how the orbital range was specified to define a 20×20 excitation window.

The excited states are calculated from single excitations involving the orbitals included in the defined excitation window (**Figure 19**). The *R_L_* quantity, representing the distance between the energy donor center (located on the ligands) and the lanthanide ion (energy density acceptor), is calculated using the coefficients of the atomic orbital contributions for the molecular orbital formation and the distances between the corresponding atoms and the lanthanide ion. These coefficients are presented in another section of the ORCA output file (not shown here).

A calculation is successfully completed only when the phrase ******ORCA TERMINATED NORMALLY****** appears, as shown in **Figure 19**.

| . . . | | | | | | |
| --- | --- | --- | --- | --- | --- | --- |
| ----------------------------- | | | | | | |
| CIS-EXCITED STATES (SINGLETS) | | | | | | |
| ----------------------------- | | | | | | |
| the weight of the individual excitations are printed if larger than 1.0e-02 | | | | | | |
|  | | | | | | |
| STATE 1: E= 0.141600 au 3.853 eV 31077.6 cm**-1 <S**2> = 0.000000 | | | | | | |
| 131a -> 169a : 0.023605 (c= 0.15363809) | | | | | | |
| 138a -> 152a : 0.826324 (c= -0.90902342) | | | | | | |
| 138a -> 161a : 0.028342 (c= 0.16835057) | | | | | | |
| 138a -> 169a : 0.026394 (c= -0.16246218) | | | | | | |
| 143a -> 152a : 0.018771 (c= 0.13700802) | | | | | | |
| 144a -> 152a : 0.015974 (c= 0.12638998) | | | | | | |
| 148a -> 152a : 0.016077 (c= -0.12679346) | | | | | | |
| . . . | | | | | | |
| STATE 25: E= 0.196252 au 5.340 eV 43072.3 cm**-1 <S**2> = 0.000000 | | | | | | |
| 130a -> 151a : 0.013064 (c= -0.11429804) | | | | | | |
| 139a -> 151a : 0.013577 (c= 0.11652131) | | | | | | |
| 140a -> 151a : 0.107311 (c= -0.32758363) | | | | | | |
| 143a -> 151a : 0.083823 (c= 0.28952175) | | | | | | |
| 148a -> 150a : 0.010827 (c= -0.10405417) | | | | | | |
| 148a -> 151a : 0.724462 (c= 0.85115308) | | | | | | |
| . . . | | | | | | |
| ******************************** | | | | | | |
| * | Entering triplet calculation | | | * | | |
| ******************************** | | | | | | |
| . . . | | | | | | |
| ----------------------------- | | | | | | |
| CIS EXCITED STATES (TRIPLETS) | | | | | | |
| ----------------------------- | | | | | | |
| the weight of the individual excitations are printed if larger than 1.0e-02 | | | | | | |
|  | | | | | | |
| STATE 1: E= 0.091084 au 2.479 eV 19990.6 cm**-1 <S**2> = 2.000000 | | | | | | |
| 143a -> 152a : 0.194349 (c= -0.44085043) | | | | | | |
| 143a -> 161a : 0.090433 (c= 0.30072117) | | | | | | |
| 148a -> 152a : 0.590625 (c= -0.76852126) | | | | | | |
| 148a -> 161a : 0.064389 (c= 0.25375053) | | | | | | |
| 148a -> 169a : 0.031329 (c= 0.17699914) | | | | | | |
| . . . | | | | | | |
| STATE 25: E= 0.154690 au 4.209 eV 33950.4 cm**-1 <S**2> = 2.000000 | | | | | | |
| 143a -> 152a : 0.147476 (c= -0.38402645) | | | | | | |
| 143a -> 161a : 0.089325 (c= -0.29887306) | | | | | | |
| 143a -> 169a : 0.199749 (c= -0.44693234) | | | | | | |
| 148a -> 152a : 0.039233 (c= -0.19807217) | | | | | | |
| 148a -> 161a : 0.129474 (c= -0.35982488) | | | | | | |
| 148a -> 169a : 0.361649 (c= -0.60137222) | | | | | | |
| . . . | | | | | | |
| Timings for individual modules:  Sum of individual times  STO integral calculation  SCF iterations  CIS module | | ...  …  …  … | 18.657 sec  0.032 sec  0.639 sec  17.986 sec | | (= 0.311 min)  (= 0.001 min)  (= 0.011 min)  (= 0.300 min) | 0.2 %  3.4 %  96.4 % |
| ****ORCA TERMINATED NORMALLY****  TOTAL RUN TIME: 0 days 0 hours 0 minutes 18 seconds 862 msec | | | | | | |

**Figure 19**. Singlet and triplet state energies and individual excitations that form the respective excited states in the .orcout file.

Module 3 – Calculation of Spectroscopic Properties

**Figure 20** shows the module for calculating spectroscopic properties, such as experimental and theoretical intensity parameters, energy transfer rates, and emission quantum yield.


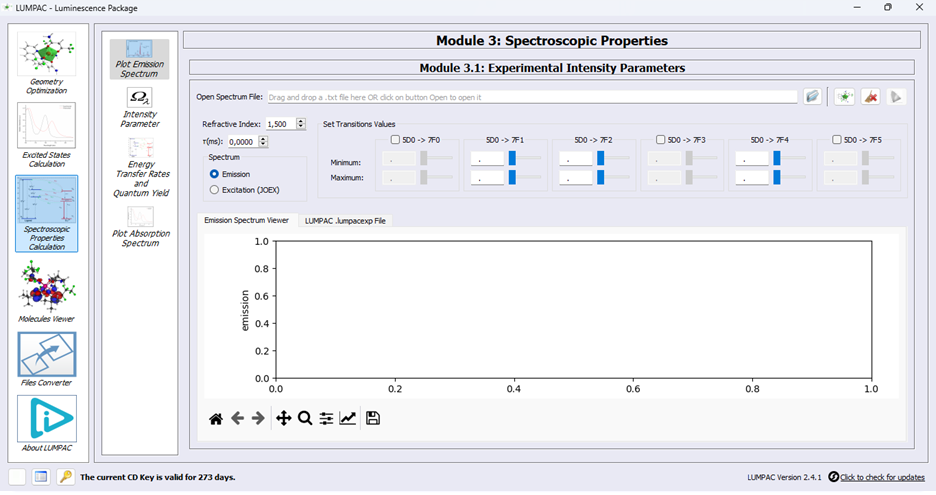


**Figure 20**. Module for spectroscopic properties calculations.

The primary objective of the theoretical protocol implemented in LUMPAC is focused on calculating the theoretical emission quantum yield. Therefore, the module for calculating the spectroscopic properties is structured into four submodules:

i) The first submodule determines the experimental intensity parameters (Ω_λ_) using the experimental emission or excitation spectrum.

ii) The second submodule calculates the theoretical intensity parameters by fitting the charge factors and polarizabilities to reproduce the experimental intensity parameters. By default, LUMPAC uses the *QDC* model ^[16]^ to adjust the intensity parameters, estimating the charge factors and polarizabilities from the adjustable *Q*, *D*, and *C* parameters, along with the atomic charge and electrophilic superdelocalizability of the coordination polyhedron atoms.

iii) The third submodule calculates the ligand-lanthanide ion energy transfer rates using the intensity parameters and the energies of the singlet and triplet excited states. If the lifetime of the ^5^D_0_ emitter level of the Eu^3+^ complex is supplied, the theoretical emission quantum yield is quantified.

iv) Finally, the fourth submodule generates the theoretical absorption spectrum using the ORCA or GAUSSIAN output files.

The following sections will detail the calculation of the cited properties using LUMPAC.

Calculation of Experimental Intensity Parameters

Warning: Users must provide the experimental emission or excitation spectrum file to calculate the experimental intensity parameters.

The experimental intensity parameters for the Eu^3+^ ion are calculated using the following equation:

|  | Eq. 1 |
| --- | --- |

where the $\chi=n(n+2)^{2}/9$ factor is known as the Lorentz local field correction term. The refractive index (*n*) varies depending on the medium; for the solid state, *n* = 1.5. 〈^7^F_2_||U^(2)^||^5^D_0_〉^2^ = 0.0032 and 〈^7^F_4_||U^(4)^||^5^D_0_〉^2^ = 0.0023 correspond to the squared reduced matrix elements of the unit operator. The *A*_01_ term, representing the radiative emission rate of the ^5^D_0_→^7^F_1_ transition, is calculated by Eq. 2. The *A*_02_ (^5^D_0_→^7^F_2_) and *A*_04_ (^5^D_0_→^7^F_4_) quantities are given by Eq*.* 3.

| $A_{01}=0,31\times10^{-11}(n)^{3}(\nu_{01})^{3}$ | Eq. 2 |
| --- | --- |
| $A_{0\lambda}=\frac{\nu_{01}}{\nu_{0J}}.\frac{S_{0\lambda}}{S_{01}}.(A_{01})$ | Eq. 3 |

The *S*_01_ and *S*_0λ_ parameters are the areas under the peaks of the ^5^D_0_→^7^F_1_ and ^5^D_0_→^7^F*_λ_* transitions, respectively. The *ν*_01_ and *ν*_0λ_ quantities are the barycenter energies of ^5^D_0_→^7^F_1_ and ^5^D_0_→^7^F_λ_, respectively. For the Eu^3+^ ion, ^5^D_0_→^7^F_1_ is assumed as the reference transition due to its magnetic dipole nature. As a result, this transition is practically independent of the chemical environment and exhibits minimal variation across different Eu^3+^ complexes.

As mentioned earlier, LUMPAC calculates the experimental intensity parameters for Eu^3+^ complexes using the experimental emission or excitation spectrum. From the emission spectrum, the areas corresponding to the ^5^D_0_→^7^F_1_, ^5^D_0_→^7^F_2_, and ^5^D_0_→^7^F_4_ transitions must be defined. For the excitation spectrum, the areas of the ^7^F_0_→[^5^D_4_, ^5^L_6_, ^5^D_2_, and ^5^D_1_] transitions are required. The ^7^F_0_→^5^D_1_ area is constant and serves as a calibration to determine Ω_2_ (^7^F_0_→^5^D_2_), Ω_4_ (^7^F_0_→^5^D_4_), and Ω_6_ (^7^F_0_→^5^L_6_). LUMPAC provides a user-friendly interface for selecting these areas.^[17]^

Procedure for Calculating Intensity Parameters and Radiative Emission Rate with LUMPAC

1. *Click on button*
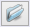
 *(***Figure *21****) to open the .txt file of the emission or excitation spectrum, where wavelengths and intensities must be separated by a comma (“,”) or a space (“ ”).*


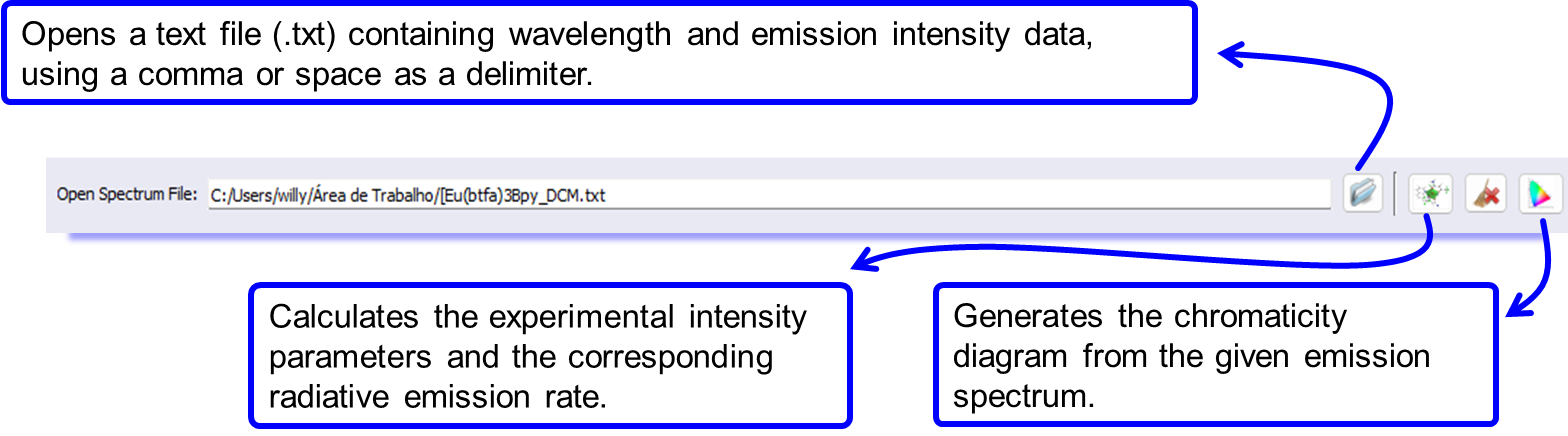


**Figure 21**. LUMPAC interface for inserting the emission or excitation spectrum and generating the chromaticity diagram.

1. *A chromaticity diagram can be generated from the selected emission spectrum by clicking on button
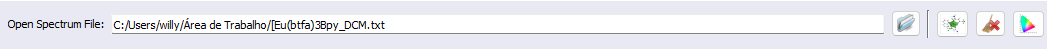
 (***Figure *21****). The chromaticity diagram of the [Eu(btfa)_3_(bpy)] complex is shown in* **Figure *22****, illustrating its emission color in the visible spectrum.*


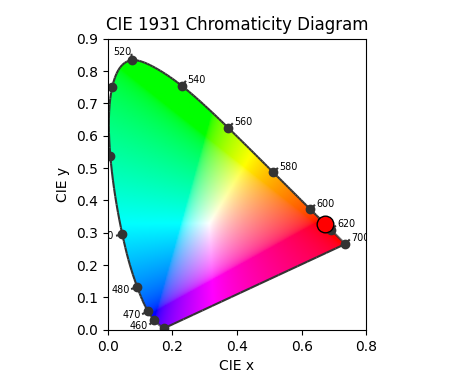


**Figure 22**. Chromaticity diagram of the [Eu(btfa)_3_(bpy)] complex in dichloromethane.

1. *For an emission spectrum, the areas of the ^5^D_0_→^7^F_1_, ^5^D_0_→^7^F_2_, and ^5^D_0_→^7^F_4_ transitions, and optionally ^5^D_0_→^7^F_0_, ^5^D_0_→^7^F_3_, and ^5^D_0_→^7^F_5_, must be appropriately selected using the LUMPAC interface (***Figure *23****).*

It is important to specify the refractive index of the medium in which the spectrum was obtained to correct for light deviations caused by the medium. The emission spectrum of the [Eu(btfa)_3_(bpy)] complex was obtained in dichloromethane (DCM), which has a refractive index of 1.424. The observed decay time (*τ*) is another essential parameter. Combining *τ* and the radiative emission rate (*A*_rad_), determined by selecting bands in the spectrum, the non-radiative emission rate (*A*_nrad_) of the complex can then be calculated.


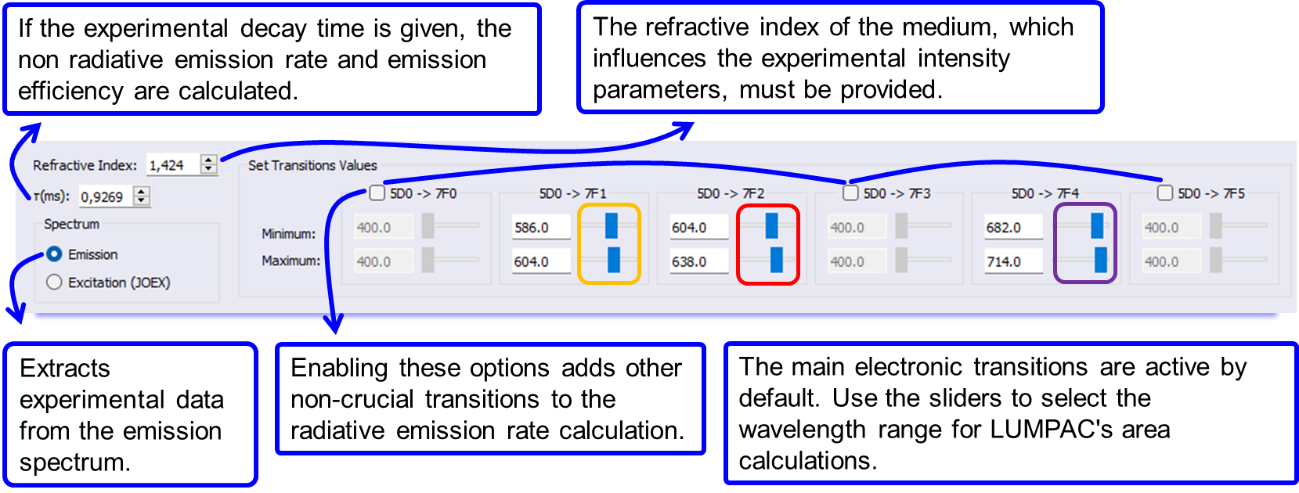


**Figure 23**. Procedure for selecting the areas under the main transitions for compounds based on the europium ion.

1. *The emission spectrum areas can be selected by numerically entering the initial and final wavelengths of the band or by using the sliders*
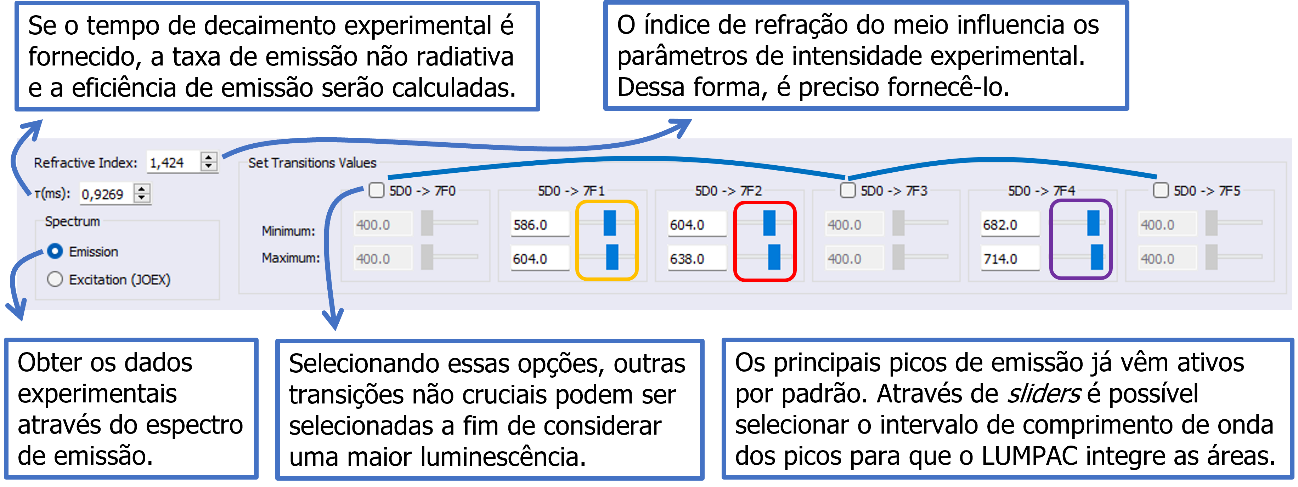
*.* **Figure *24*** *shows all possible selections.*


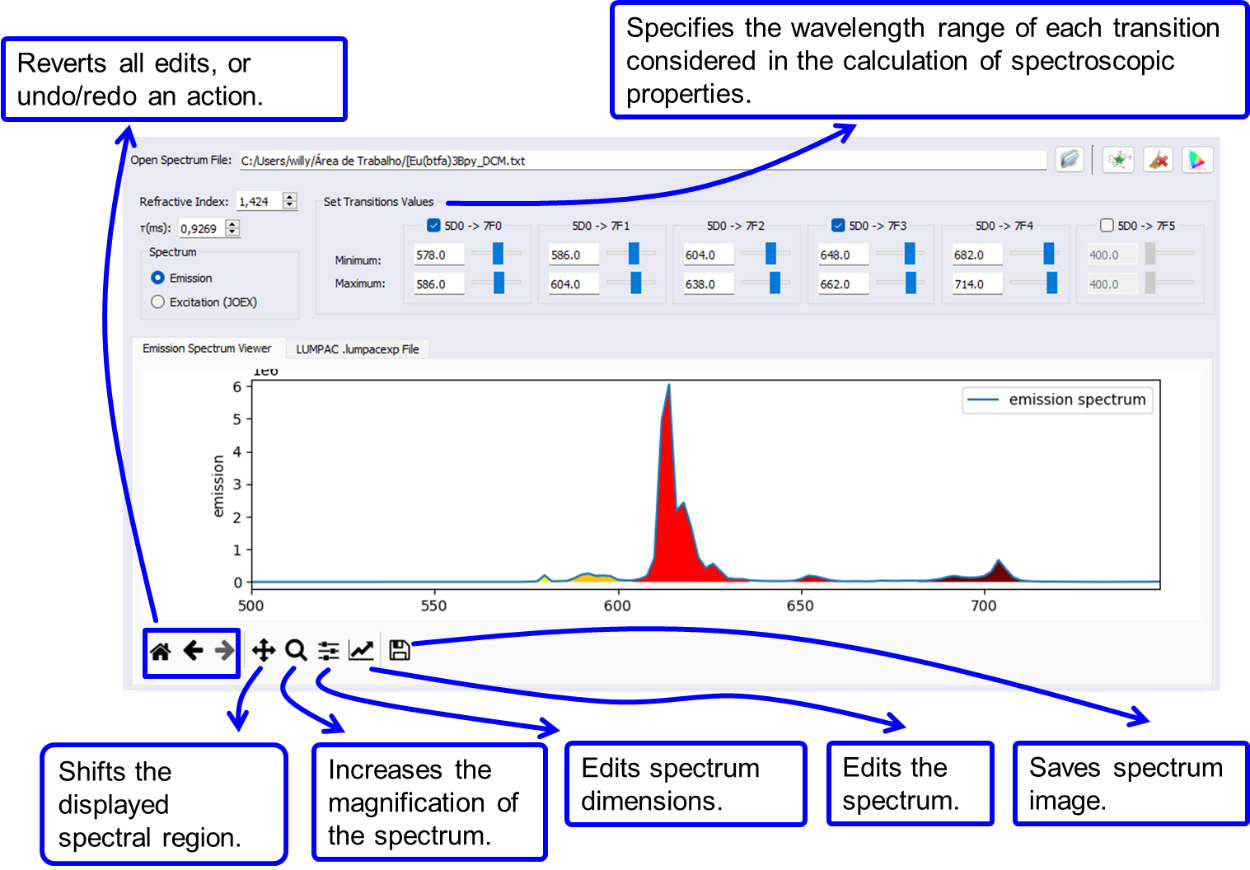


**Figure 24**. Emission spectrum viewer with all possible areas selected and spectrum visualization options.

1. *To calculate the spectroscopic properties using an excitation spectrum, select the “Excitation (JOEX)” option (***Figure *25****). The interface operates similarly to that of the emission spectrum, but refractive indices must be added individually for each band.*


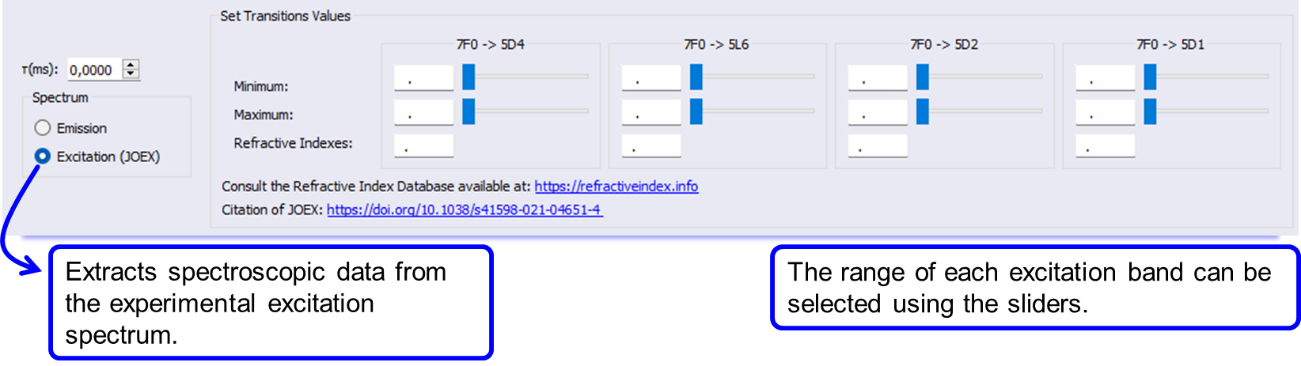


**Figure 25**. Interface for selecting the bands of the excitation spectrum.

1. *After selecting the bands from the emission or excitation spectrum and adding the required data, click on button
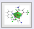
 (***Figure *21*** *and* **Figure *24****) to execute the calculation of the experimental intensity parameters and radiative emission rate.*

Warning: The data is saved in a file with the .lumpacexp extension, which adopts the same filename as the corresponding spectrum .txt file.

Theoretical Calculation of Intensity Parameters

**Figure 26** shows the LUMPAC submodule, which calculates the theoretical intensity parameters. A nonlinear algorithm adjusts the charge factors (*g*) and polarizabilities (*α*), used in the calculation of the $\gamma_{p}^{t}$ and $\Gamma_{p}^{t}$ parameters, respectively, to reproduce the experimental values of Ω_2_ and Ω_4_.


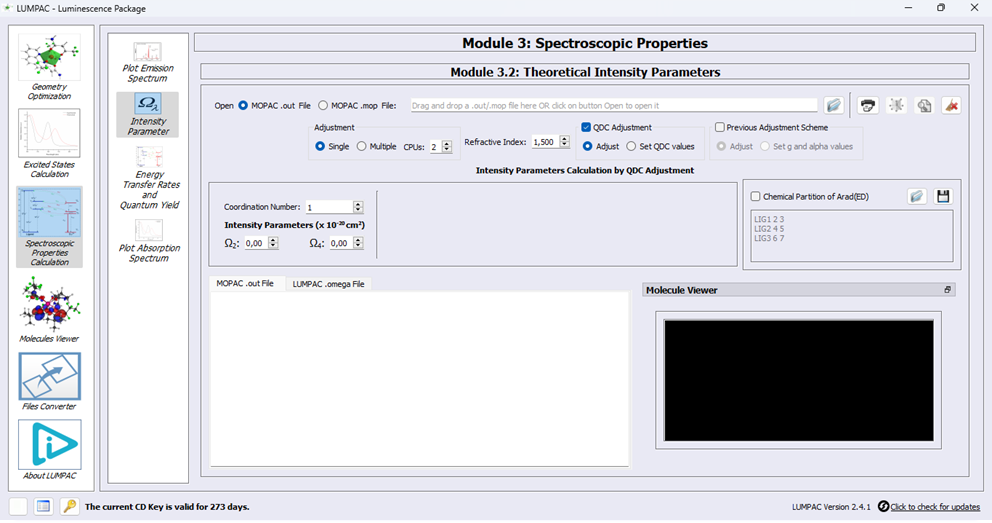


**Figure 26**. LUMPAC submodule for calculating the theoretical intensity parameters.

Procedure for Calculation of the Theoretical Intensity Parameters using LUMPAC

1. *Click on button
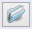
 (***Figure *27****) to open the MOPAC output file (.out) containing the optimized geometry or the MOPAC input file (.mop) with the initial structure.*

**Figure 27** lists all quantities that can be included in the output file of the submodule for calculating the intensity parameters. By default, theoretical intensity parameters (Ω_λ_), forced electric dipole parameters (Ω_λ_^ED^), charge factors (*g*), and polarizabilities (*α*) are always calculated. Other data, such as the spherical coordinates of the coordination polyhedron, theoretical radiative emission rate (*A*_rad_), and the effect of each ligand on *A*_rad_, are selected by default but can be omitted, and additional options can be selected.


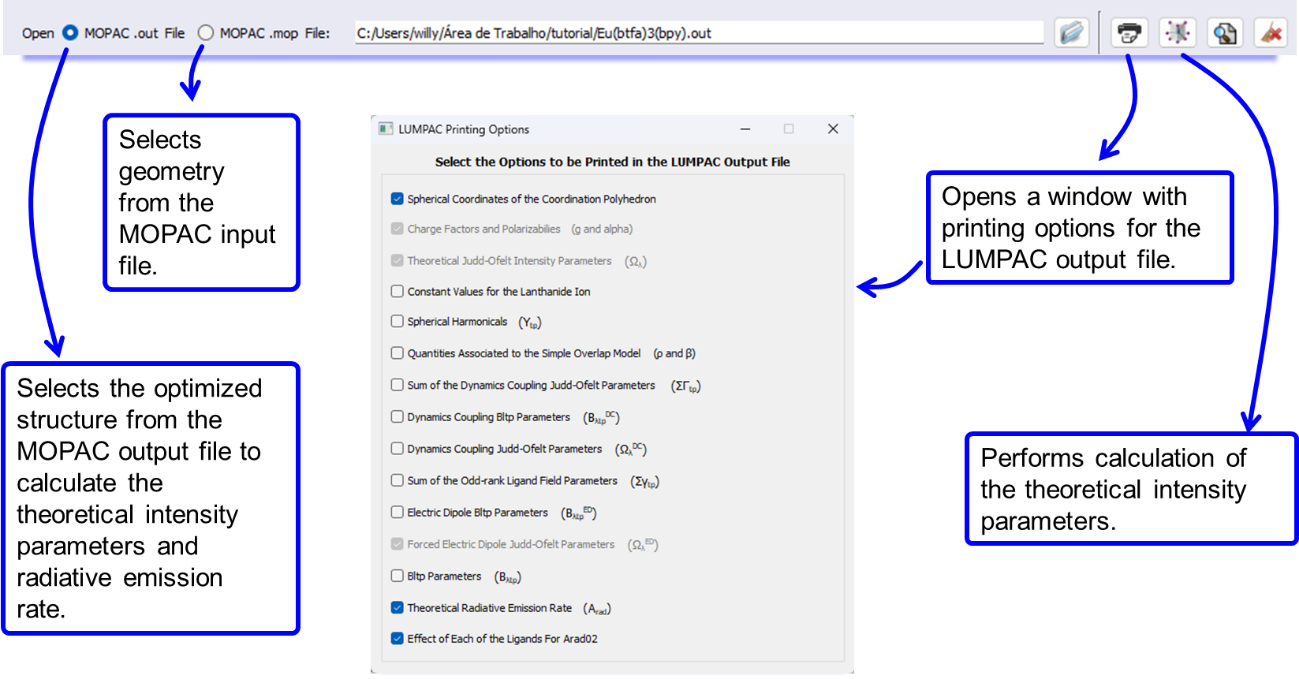


**Figure 27**. Input files supported by LUMPAC in calculation of the theoretical intensity parameters and optional quantities for the output file.

1. *In LUMPAC 2.0, there are four different procedures for calculating the theoretical intensity parameters (***Figure *28****).*

LUMPAC allows the simultaneous calculation of the theoretical intensity parameters for multiple files in the same directory by selecting the “Multiple” option (**Figure 28**). For LUMPAC to recognize the experimental intensity parameters for each complex, the .lumpacexp and .out files must have identical prefixes.


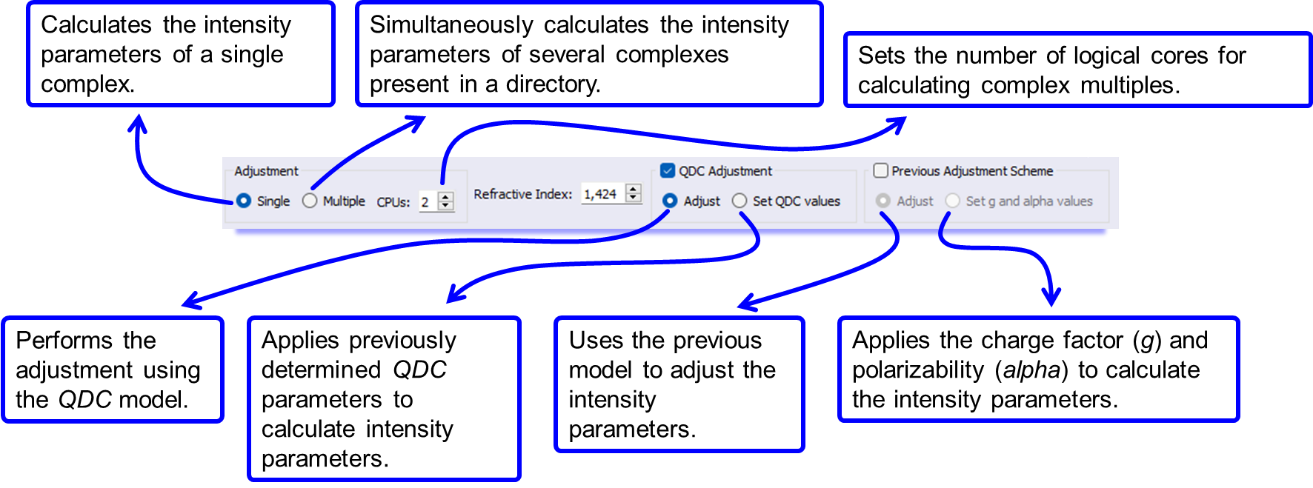


**Figure 28**. LUMPAC 2.0 interface for selecting the method of fitting theoretical intensity parameters.

- 1. *By default, LUMPAC uses the QDC fitting (***Figure *29****). The g and α quantities, within physical limits, are calculated using three adjustable parameters (Q, D, and C).*

The intensity parameters and the coordination number can be manually entered if they are not automatically loaded (**Figure 29**). The *QDC* fitting, initially implemented in the first version of LUMPAC and continued in version 2.0, assigns a different *g* and *α* to each ligand atom, because these quantities are estimated as a function of the charge and electrophilic superdelocalizability of each atom, which vary among atoms. When applying the *QDC* fitting, the chemical partitioning can also be performed, accounting for the contribution of each ligand to *A_rad_*, as illustrated in **Figure 30**.


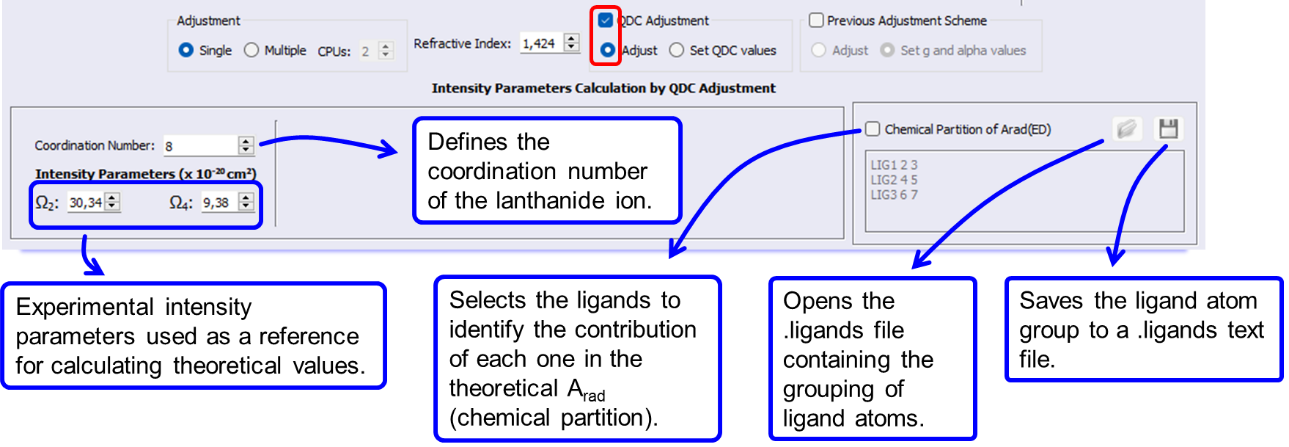


**Figure 29**. LUMPAC interface dedicated to the QDC fitting.

- 1. *Another option is to manually add the Q, D, and C values from a previous calculation, as shown in* **Figure *30****.*


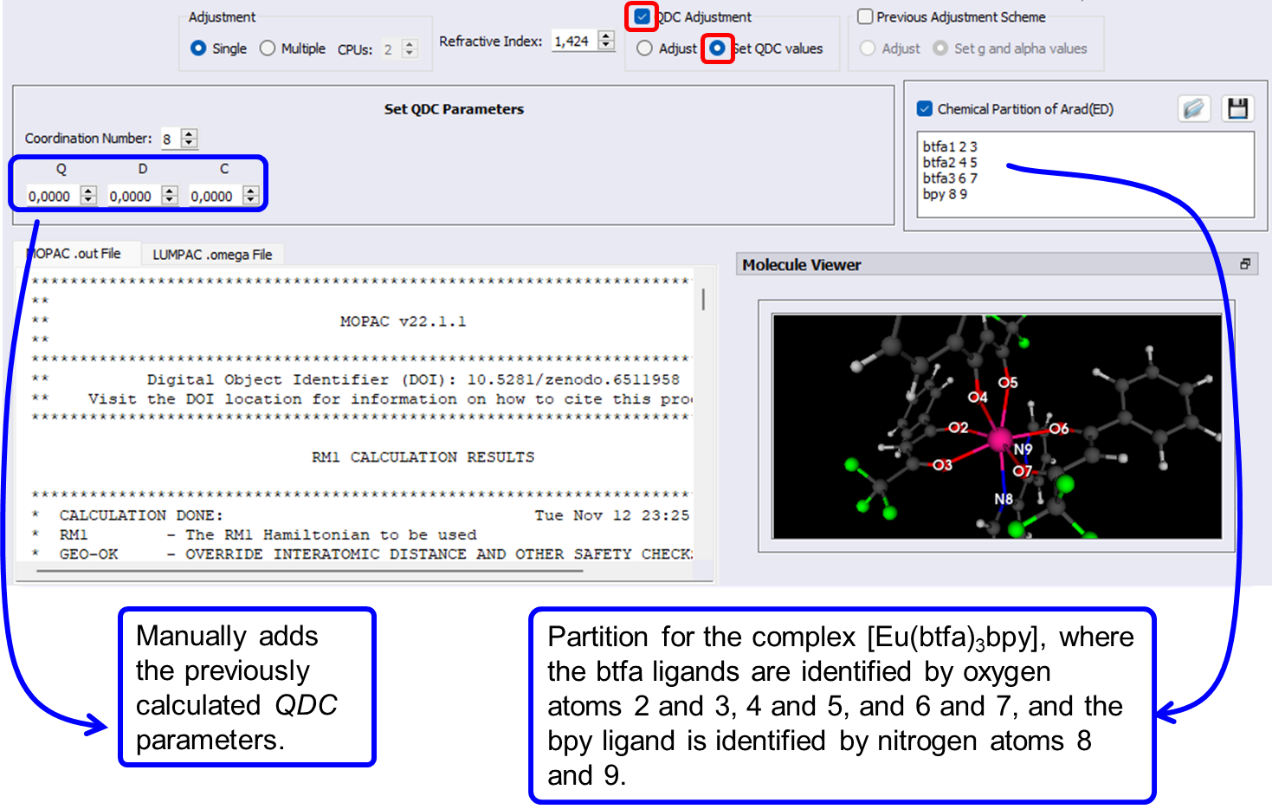


**Figure 30**. Interface for manual input of the QDC parameters and ligand labels assignment to evaluate the chemical partitioning of the ligands on Arad.

- 1. An alternative procedure for calculating intensity parameters applies to the previous adjustment, used by default before the implementation of the *QDC* model. In the previous adjustment, *g* and *α* for the ligand atoms are arbitrarily grouped based on their chemical environment. LUMPAC provides a simple way for grouping atoms that share the same chemical environment *(***Figure *31****).*

The following atoms are grouped together (**Figure 31**) for the [Eu(btfa)_3_(bpy)] complex: N8 and N9 (bipyridine nitrogens); O2, O3, O4, O5, O6, and O7 (β-diketone oxygens). Users can drag the ligand atoms from the "Ligand Atoms" box to their respective groups.


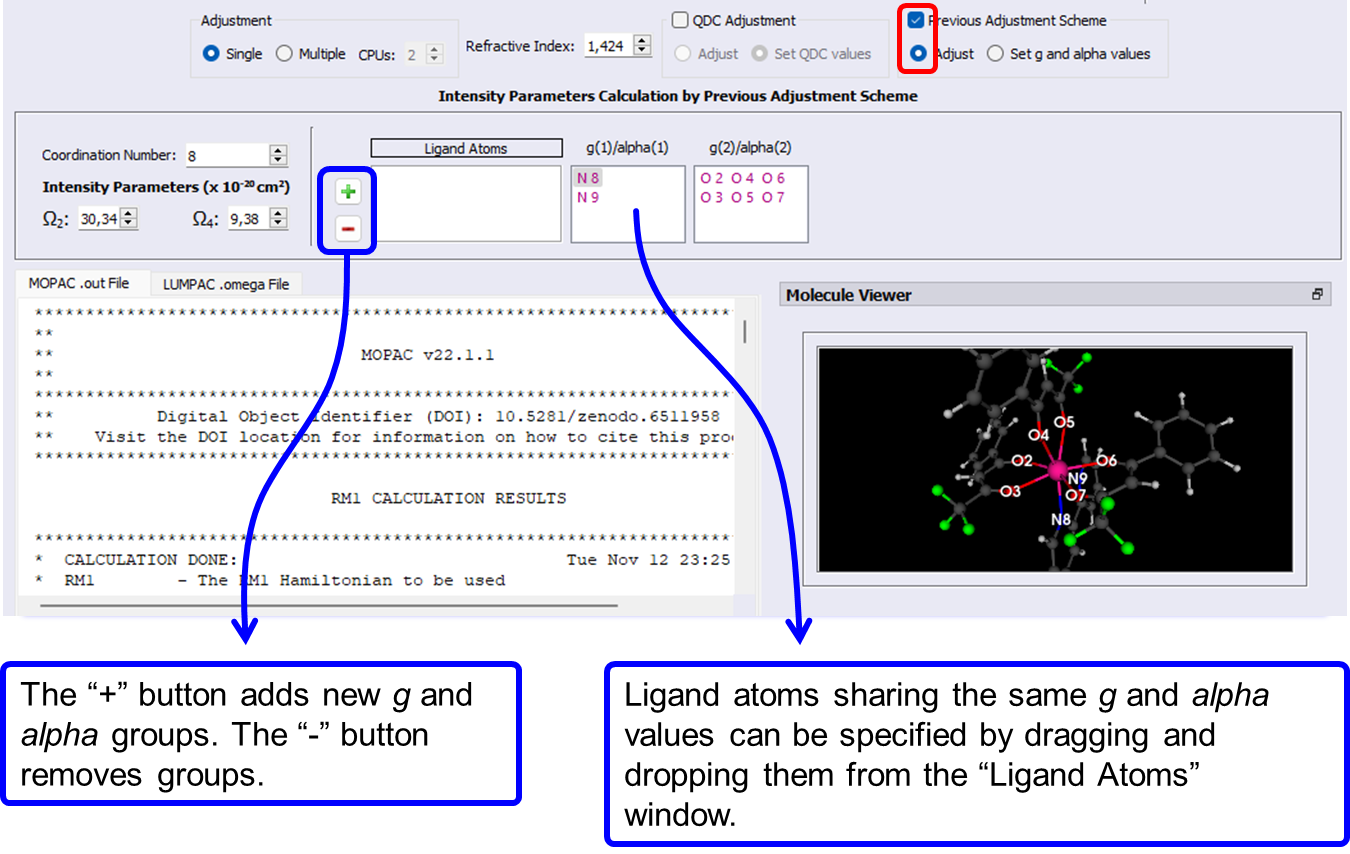


**Figure 31**. LUMPAC interface for defining the groups of ligand atoms based on the charge factors and polarizabilities.

- 1. *Finally, g and α determined previously can be manually entered for each ligand atom, as shown in* **Figure *32****.*


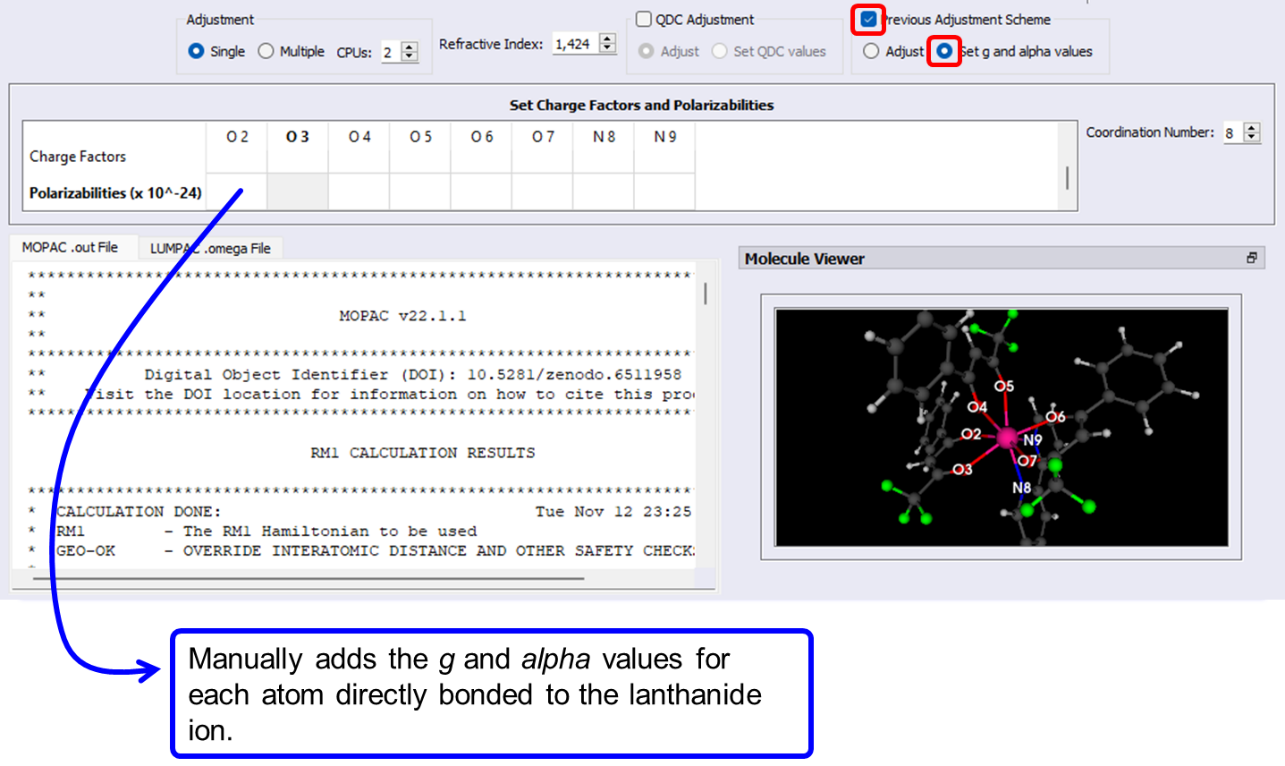


**Figure 32**. LUMPAC interface for manual insertion of g and α.

Because the radiative emission rate depends on Ω_6_, which is not measured experimentally, the calculations of the theoretical intensity parameters are essential. These calculations determine the contributions of the dynamic coupling (Ω_λ_^DC^) and the electric dipole (Ω_λ_^ED^) mechanisms, and Ω_λ_^ED^ is useful for calculating the ligand-metal ion energy transfer rates via the multipolar mechanism.

Calculation of Energy Transfer Rate and Emission Quantum Yield

**Figure 33** shows the module for calculating the energy transfer rates and, if the lifetime is provided, the emission quantum yield. These quantities are calculated using the intensity parameters estimated in the previous section, and the excited state energies contained in the ORCA output file.


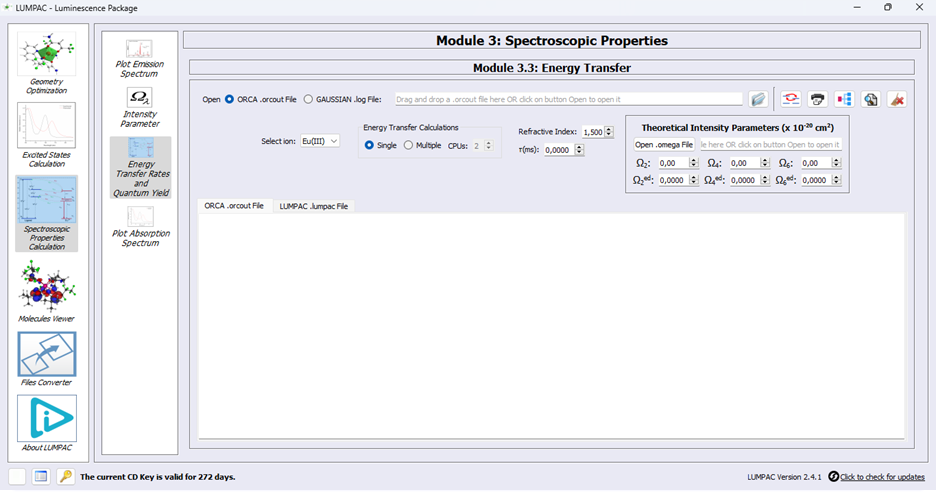


**Figure 33**. Module for calculating energy transfer rates and emission quantum yield.

Procedure for Calculating Energy Transfer Rates and Emission Quantum Yield using LUMPAC

1. *Click on button
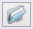
 (***Figure *34****) to open the ORCA output file (.orcout), generated by the procedure in Module 2.*

In LUMPAC 2.0, in addition to using the semiempirical excited states calculated in Module 2, data from DFT calculations performed with the ORCA and GAUSSIAN programs can be used. Another new feature of LUMPAC 2.0 is the possibility of calculating the energy transfer rates for Tb^3+^ complexes. The choice of the ion in question can be made as shown in **Figure 34***.* However, the theoretical quantum yield cannot yet be determined Tb^3+^.

1. *When the .orcout file is opened, the .omega file containing the previously calculated intensity parameters (****Figure 34****) will load automatically. To enable this automatic loading, the .orcout and .omega files must be in the same directory and have identical base names.*

Attention: If the .omega file is not loaded automatically, manually provide the intensity parameters calculated through the LUMPAC interface or specify the corresponding .omega file.


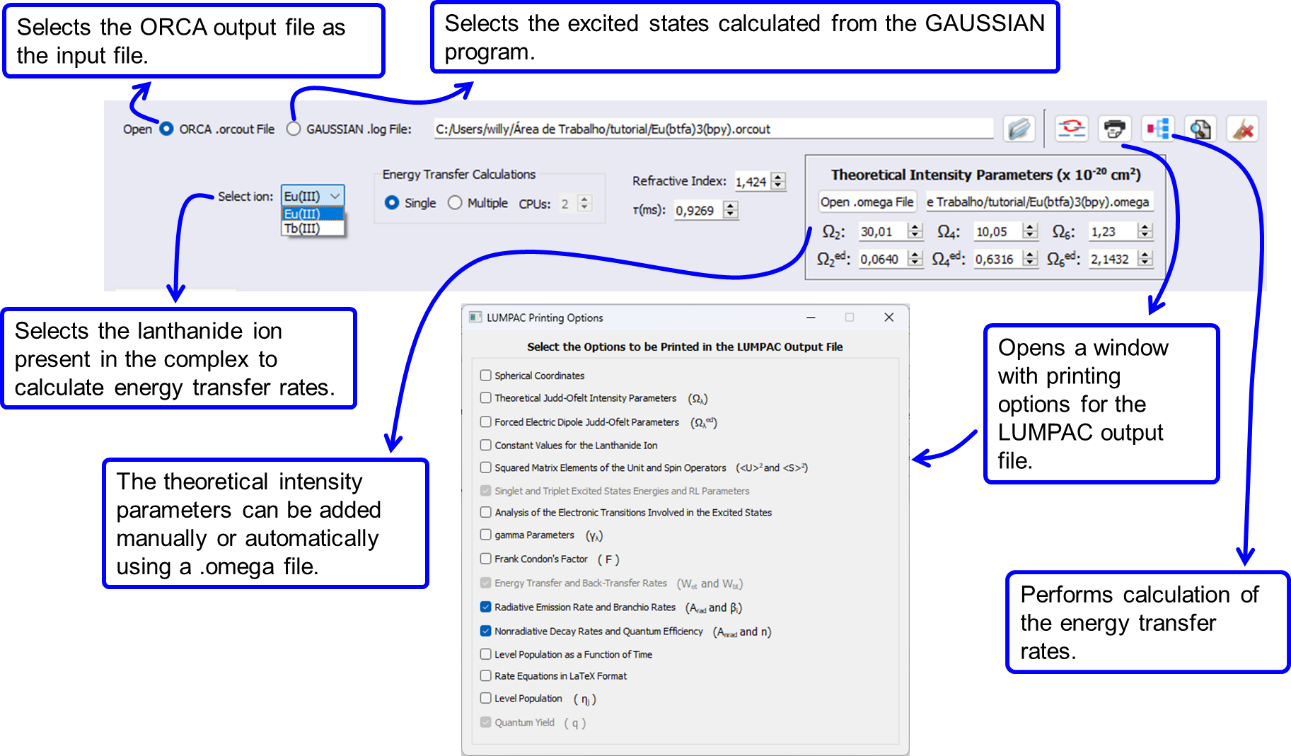


**Figure 34**. Calculation of the energy transfer rates and emission quantum yield from the ORCA output file and theoretical intensity parameters.

1. *To modify the energy transfer channels, intraligand decay rates, ligand states, and typical values of quantities important for calculating the ligand-lanthanide ion energy transfer rates, users must click on button
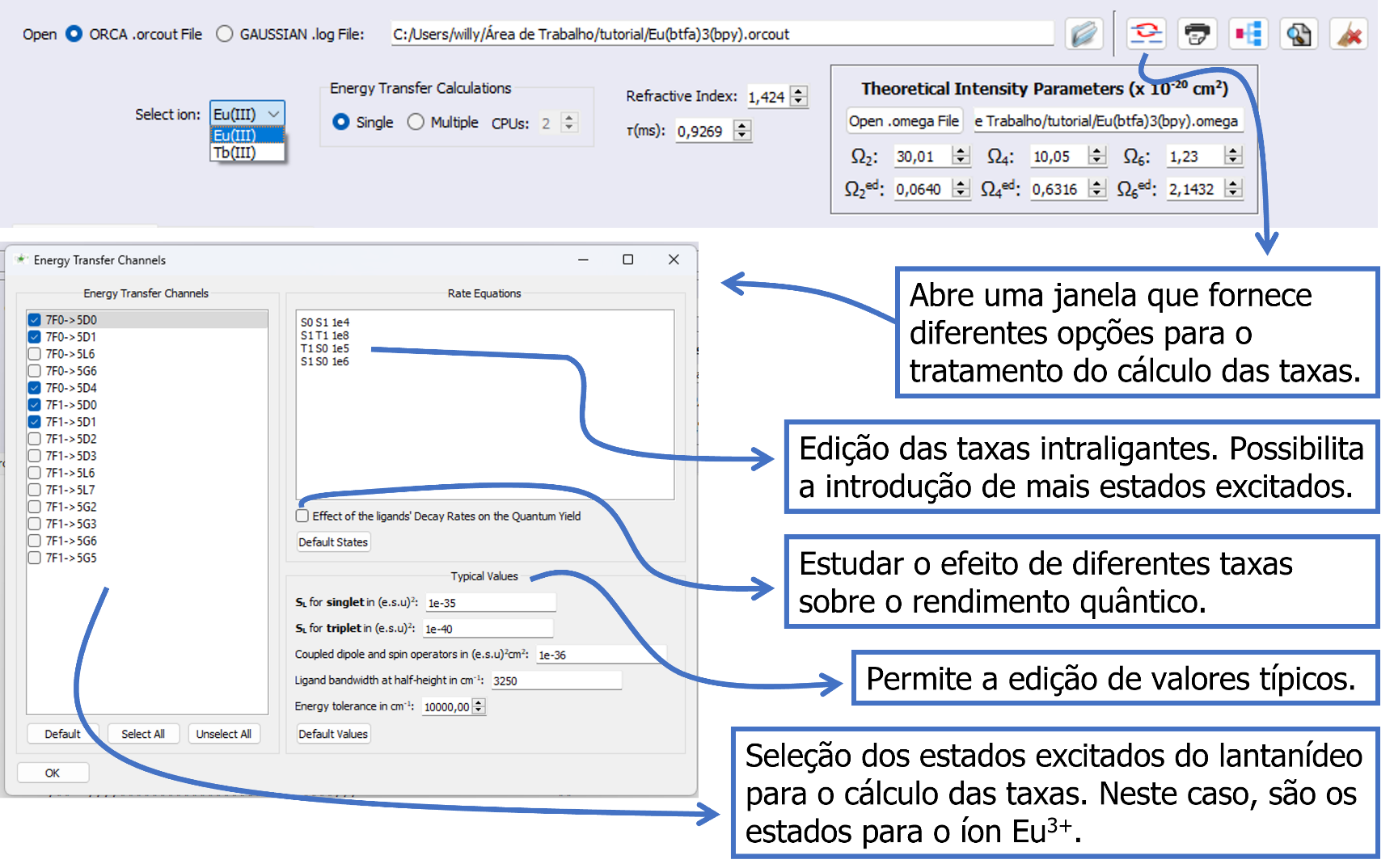
 (***Figure *35****).*

It is possible to select energy transfer channels involving various excited acceptor levels of the Eu^3+^ ion: ^5^D_0_, ^5^D_1_, ^5^D_2_, ^5^D_3_, ^5^D_4_, ^5^L_6_, ^5^L_7_, ^5^G_2_, ^5^G_3_, ^5^G_5_, and ^5^G_6_, excited from the ^7^F_0_ or ^7^F_1_ ground states. For Tb^3+^, the available acceptor levels are ^5^D_3_, ^5^D_4_, ^5^G_4_, ^5^G_5_, ^5^G_6_, ^5^L_6_, ^5^L_10_, ^5^H_5_, ^5^H_6_, ^5^H_7_, and ^5^F_5_, excited from the ^7^F_5_ or ^7^F_6_ ground states. In LUMPAC 2.0, several different ligand levels can be considered in the rate equations. The S_0_, S_1_, and T_1_ states are automatically selected by LUMPAC with the following typical rate values: 1×10^4^ s^-1^ for the S_0_→S_1_ channel and 1×10^8^ s^-1^, 1×10^5^ s^-1^, and 1×10^6^ s^-1^ for the S_1_→T_1_, T_1_→S_0_, e S_1_→S_0_ channels, respectively (**Figure 35**). However, both the levels and rate values can be manually modified. To consider a new interaction between levels, simply provide the initial state, the final state, and the respective rate. For example, to include the T_5_ state with decay rate of 5×10^9^ s^-1^ for S_1_→T_5_ and 1×10^10^ s^-1^ for T_5_→T_1_, two lines must be added in the “Rate Equations” window (**Figure 35**): “S1 T1 5e9” and “T5 T1 1e10”.


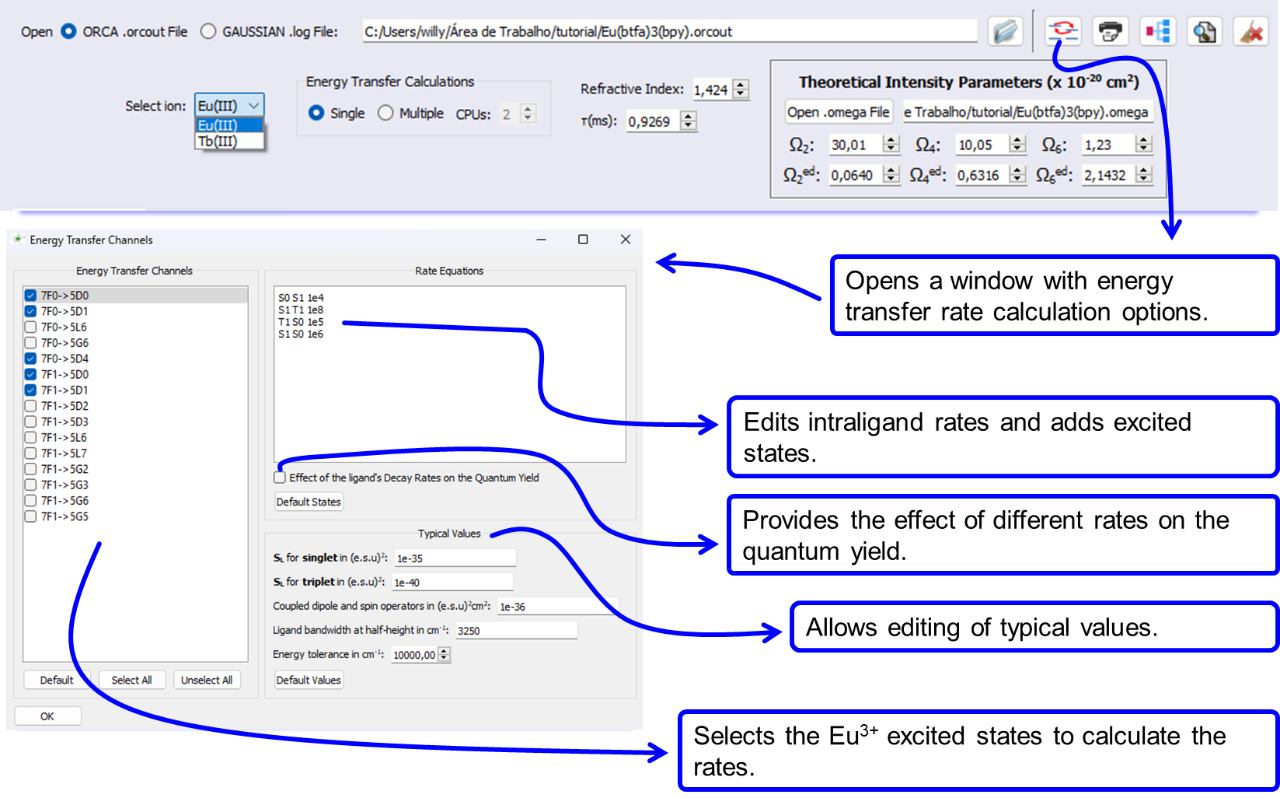


**Figure 35**. Interface for modifying parameters for calculating the energy transfer rates.

When the “Effect of the ligand’s Decay Rates on the Quantum Yield” option is selected (**Figure 35**), LUMPAC varies the decay rates involving the ligand states. This process generates all possible combinations of intraligand decay rates from 1×10^0^ s^-1^ to 1×10^12^ s^-1^, calculating the theoretical quantum yield for each combination. The results are stored in a .simul file, saved in the directory of the .orcout file.

1. *Click on button
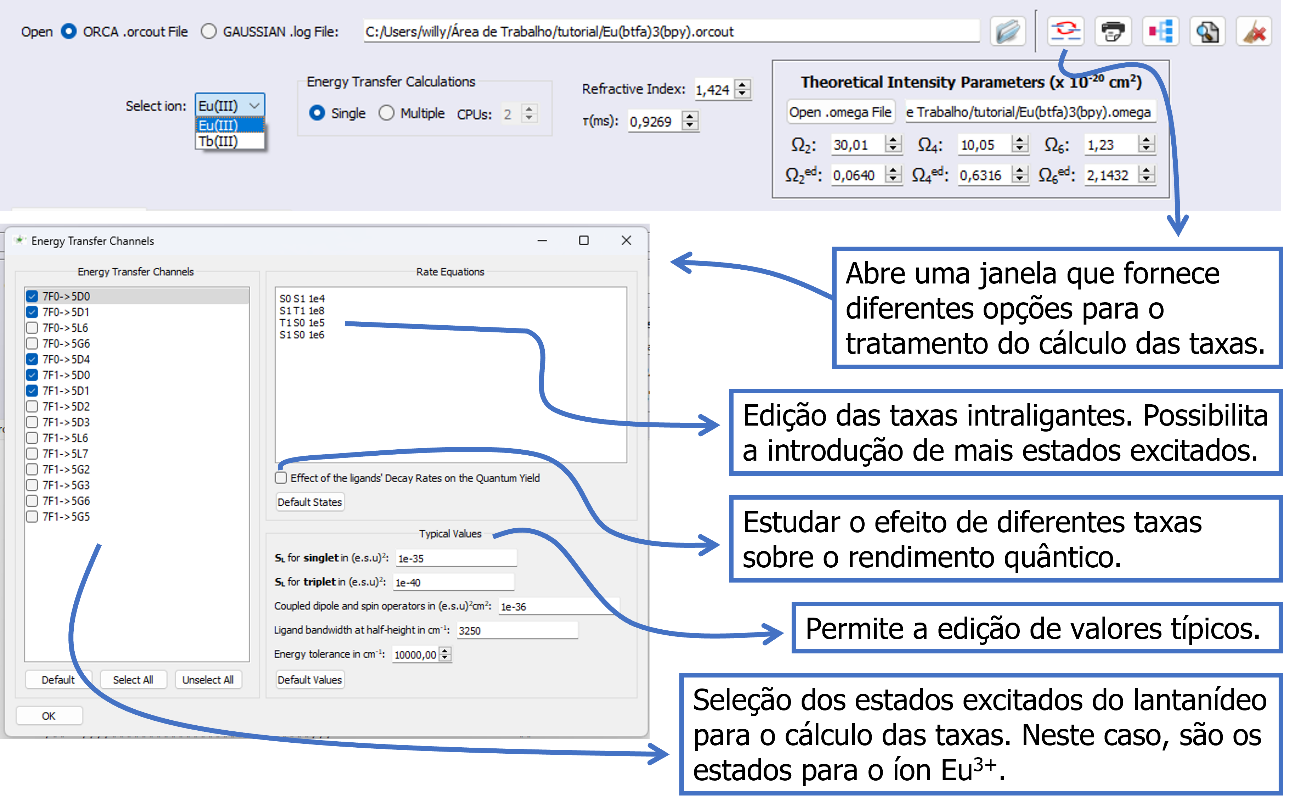
 (***Figure *34*** *and* **Figure *35****) to execute the calculation of energy transfer and back-transfer rates using the selected parameters. If the lifetime is provided, the emission quantum yield will also be calculated.*

The .simul file with the results of the emission quantum yield evaluation as a function of the intraligand decay rates for the [Eu(btfa)_3_(bpy)] complex is shown in **Figure 36**. The columns labeled “S1_T1”, “T1_S0”, and “S1_S0” refer to the S_1_→T_1_, T_1_→S_0_, and S_1_→S_0_ decay rates, respectively. If an additional ligand level were included in the Rate Equations edit field (**Figure 36**), it would also be incorporated into the analysis. The “s_calc” column is the theoretical sensitization efficiency, calculated as the ratio between theoretical quantum yield and efficiency, while the “s_exp” column represents the experimental sensitization efficiency. The “yield” column displays the emission quantum yield for each rate combination, limited by the theoretical quantum efficiency. The “erro_s” column shows the error between the theoretical and experimental sensitization efficiency. The final three lines of **Figure 36** present the correlation coefficient between the rates for each transfer channel and the quantum yield of the complex. Essentially, the correlation coefficient reveals how much each transfer channel influences the quantum yield. A positive value means that increasing the rate of that channel increases the quantum yield, while a negative value indicates that it decreases it. The absolute value of the coefficient indicates the strength of the effect.

| 1 | S1_T1 | T1_S0 | | S1_S0 | | s_exp | s_calc | yield | error_s |
| --- | --- | --- | --- | --- | --- | --- | --- | --- | --- |
| 2 | 1e12 | 1e0 | | 1e0 | | 100.00 | 100.00 | 87.20 | 0.0000 |
| 3 | 1e11 | 1e0 | | 1e0 | | 100.00 | 100.00 | 87.20 | 0.0000 |
| 4 | 1e12 | 1e0 | | 1e1 | | 100.00 | 100.00 | 87.20 | 0.0000 |
| ... |  |  | |  | |  |  |  |  |
| 825 | 1e12 | 1e8 | | 1e10 | | 100.00 | 83.84 | 73.11 | 16.1572 |
| 826 | 1e5 | 1e8 | | 1e4 | | 100.00 | 81.70 | 71.24 | 18.2959 |
| 827 | 1e4 | 1e8 | | 1e4 | | 100.00 | 80.33 | 70.04 | 19.6740 |
| ... |  |  | |  | |  |  |  |  |
| 2196 | 1e2 | 1e12 | | 1e12 | | 100.00 | 0.00 | 0.00 | 100.0000 |
| 2197 | 1e1 | 1e12 | | 1e12 | | 100.00 | 0.00 | 0.00 | 100.0000 |
| 2198 | 1e0 | 1e12 | | 1e12 | | 100.00 | 0.00 | 0.00 | 100.0000 |
| 2199 |  |  | |  | |  |  |  |  |
| 2200 |  |  | |  | |  |  |  |  |
| 2201 | S1->T1 | | 0.1769 | |  | | | | |
| 2202 | T1->S0 | | -0.3040 | |  | | | | |
| 2203 | S1->S0 | | -0.2772 | |  | | | | |

**Figure 36**. Eu(btfa)_3_(bpy).simul file displaying the emission quantum yield as function of the S_1_→T_1_, T_1_→S_0_, and S_1_→S_0_ decay rates.

After the calculation finishes, an energy diagram is generated, as illustrated in **Figure 37**. This diagram displays the selected energy levels of the ligands and the Eu^3+^ ion on a common scale and also highlights the specified energy transfer and back-transfer channels.


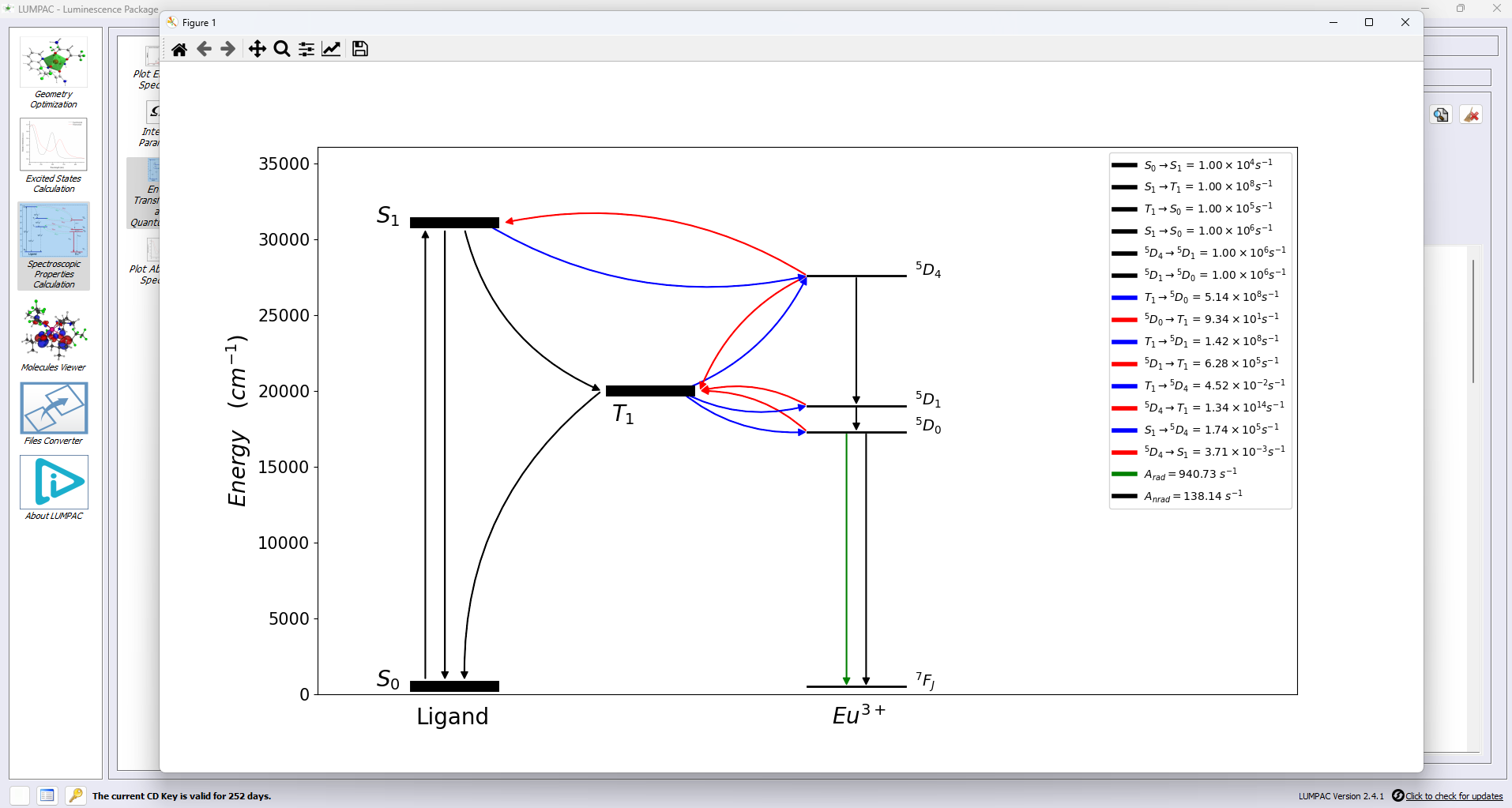


**Figure 37**. Energy diagram of the [Eu(btfa)_3_(bpy)] complex generated by LUMPAC after calculating the energy transfer rates.

**Figure 38** shows the Eu(btfa)3(bpy).lumpac output file containing all the calculated properties, with all options enabled in the “LUMPAC Printing Options” window (**Figure 34**).

| ********************************************************************************** | | | | | | | | | | | | | | | | | | | | |
| --- | --- | --- | --- | --- | --- | --- | --- | --- | --- | --- | --- | --- | --- | --- | --- | --- | --- | --- | --- | --- |
|  | | |  | | |  | | |  | |  | | |  | | |  | | |  |
| ## | | | ## ## | | | ## ## | | | ##### | | #### | | | #### | | | ##### | | | #### |
| ## | | | ## ## | | | ### ### | | | ## ## | | ## ## | | | ## ## | | | ## | | | ## ## |
| ## | | | ## ## | | | ## # ## | | | ##### | | ###### | | | ## | | | #### | | | ## ## |
| ## | | | ## ## | | | ## ## | | | ## | | ## ## | | | ## ## | | | ## ## | | | ## ## |
| ###### | | | #### | | | ## ## | | | ## | | ## ## | | | #### | | | ###### ## | | | #### |
|  | | | | | | | | | | | | | | | | | | | | |
| LUMPAC - Lanthanide Luminescence Software - version 2.0 | | | | | | | | | | | | | | | | | | | | |
| Cite this work as: | | | | | Dutra, J. D. L.; Bispo, T. D.; Freire, R. O. | | | | | | | | | | | | | | | |
|  | | | | | LUMPAC Lanthanide Luminescence Software: efficient and user friendly | | | | | | | | | | | | | | | |
|  | | | | | Journal of Computational Chemistry, 2014, 35, 772_775. | | | | | | | | | | | | | | | |
|  | | | | | http://dx.doi.org/10.1002/jcc.23542 | | | | | | | | | | | | | | | |
|  | | | | | DOI:10.1002/jcc.23542 | | | | | | | | | | | | | | | |
| ********************************************************************************** | | | | | | | | | | | | | | | | | | | | |
|  | | | | | | | | | | | | | | | | | | | | |
| The data shown below were calculated from the geometry and excited states energies read in following file: | | | | | | | | | | | | | | | | | | | | |
| C:/Users/willy/Área de Trabalho/tutorial/Eu(btfa)3(bpy).orcout | | | | | | | | | | | | | | | | | | | | |
|  | | | | | | | | | | | | | | | | | | | | |
| ----------------------------------------------------------- | | | | | | | | | | | | | | | | | | | | |
| Forced Electric Dipole Judd-Ofelt Parameters, omegaED (l) | | | | | | | | | | | | | | | | | | | | |
| ----------------------------------------------------------- | | | | | | | | | | | | | | | | | | | | |
|  | | | | | | | | | | | | | | | | | | | | |
| omegaED(2) = 6.4000e-22 cm^2 | | | | | | | | | | | | | | | | | | | | |
| omegaED(4) = 6.3160e-21 cm^2 | | | | | | | | | | | | | | | | | | | | |
| omegaED(6) = 2.1432e-20 cm^2 | | | | | | | | | | | | | | | | | | | | |
|  | | | | | | | | | | | | | | | | | | | | |
| ----------------------------------- | | | | | | | | | | | | | | | | | | | | |
| Constant values for the Ln3+ Ion | | | | | | | | | | | | | | | | | | | | |
| ----------------------------------- | | | | | | | | | | | | | | | | | | | | |
|  | | | | | | | | | | | | | | | | | | | | |
| Racah Tensor Operator Used: <f\|C(2)\|f> = -1.3660 <f\|C(4)\|f> = 1.1280 <f\|C(6)\|f> = -1.2700 | | | | | | | | | | | | | | | | | | | | |
|  | | | | | | | | | | | | | | | | | | | | |
| Shielding Factor Used: sigma(2) = 0.6000 sigma(4) = 0.1390 sigma(6) = 0.1000 | | | | | | | | | | | | | | | | | | | | |
|  | | | | | | | | | | | | | | | | | | | | |
| Radial Integrals: r(2) = 2.5693e-17 cm^2 r(4) = 1.5840e-33 cm^4 r(6) = 1.9848e-49 cm^6 | | | | | | | | | | | | | | | | | | | | |
|  | | | | | | | | | | | | | | | | | | | | |
| SL for singlet = 1.00e-35 (e.s.u)^2 | | | | | | | | | | | | | | | | | | | | |
|  | | | | | | | | | | | | | | | | | | | | |
| SL for triplet = 1.00e-40 (e.s.u)^2 | | | | | | | | | | | | | | | | | | | | |
|  | | | | | | | | | | | | | | | | | | | | |
| Coupled dipole and spin operators = 1.00e-36 (e.s.u)^2 cm^2 | | | | | | | | | | | | | | | | | | | | |
|  | | | | | | | | | | | | | | | | | | | | |
| Ligand bandwidth at half-height = 3250.00 cm^-1 | | | | | | | | | | | | | | | | | | | | |
|  | | | | | | | | | | | | | | | | | | | | |
| -------------------------------------------------------- | | | | | | | | | | | | | | | | | | | | |
| Squared matrix elements of the unit and spin operators | | | | | | | | | | | | | | | | | | | | |
| -------------------------------------------------------- | | | | | | | | | | | | | | | | | | | | |
|  | | | | | | | | | | | | | | | | | | | | |
| Donor | | Acceptor | | | <U(2)>^2 | | | | | <U(4)>^2 | | | <U(6)>^2 | | <S>^2 | | | | | |
| 7F0 | | 5D0 | | | 0.0032 | | | | | 0.0000 | | | 0.0000 | | 0.0000 | | | | | |
| 7F0 | | 5D1 | | | 0.0000 | | | | | 0.0000 | | | 0.0000 | | 0.0273 | | | | | |
| 7F0 | | 5D4 | | | 0.0000 | | | | | 0.0011 | | | 0.0000 | | 0.0000 | | | | | |
| 7F1 | | 5D0 | | | 0.0000 | | | | | 0.0000 | | | 0.0000 | | 0.1170 | | | | | |
| 7F1 | | 5D1 | | | 0.0025 | | | | | 0.0000 | | | 0.0000 | | 0.0000 | | | | | |
|  | | | | | | | | | | | | | | | | | | | | |
| ------------------------------------------------- | | | | | | | | | | | | | | | | | | | | |
| Singlet and Triplet Excited States Energies and | | | | | | | | | | | | | | | | | | | | |
| RL Parameters | | | | | | | | | | | | | | | | | | | | |
| ------------------------------------------------- | | | | | | | | | | | | | | | | | | | | |
|  | | | | | | | | | | | | | | | | | | | | |
| Chosen Triplet Excited State = T1 | | | | | | | | | | | | | | | | | | | | |
| RL Triplet: 4.4793 Angs Triplet Energy: 19990.60 cm^-1 | | | | | | | | | | | | | | | | | | | | |
|  | | | | | | | | | | | | | | | | | | | | |
| Chosen Singlet Excited State = S1 | | | | | | | | | | | | | | | | | | | | |
| RL Singlet: 4.1648 Angs Singlet Energy: 31077.60 cm^-1 | | | | | | | | | | | | | | | | | | | | |
|  | | | | | | | | | | | | | | | | | | | | |
| RL: distance from the donor state located at the organic ligands | | | | | | | | | | | | | | | | | | | | |
| and the Ln3+ ion nucleus. | | | | | | | | | | | | | | | | | | | | |
|  | | | | | | | | | | | | | | | | | | | | |
|  | | | | | | | | | | | | | | | | | | | | |
| ---------------------------------------------------------- | | | | | | | | | | | | | | | | | | | | |
| Transitions with coeff less than 5.0% will be neglected. | | | | | | | | | | | | | | | | | | | | |
| ---------------------------------------------------------- | | | | | | | | | | | | | | | | | | | | |
|  | | | | | | | | | | | | | | | | | | | | |
| State T1 Fosc = 0.0000 RL = 4.4793 Angs E = 19990.60 cm^-1 lambda = 500.24 nm | | | | | | | | | | | | | | | | | | | | |
|  | | | | | | | | | | | | | | | | | | | | |
| HOMO->LUMO+3 | | | | | 59.06% | | |  | | | | | | | | | | | | |
| HOMO-5->LUMO+3 | | | | | 19.43% | | |  | | | | | | | | | | | | |
| HOMO-5->LUMO+12 | | | | | 9.04% | | |  | | | | | | | | | | | | |
| HOMO->LUMO+12 | | | | | 6.44% | | |  | | | | | | | | | | | | |
| TOTAL | | | | | 93.98% | | |  | | | | | | | | | | | | |
|  | | | | | | | | | | | | | | | | | | | | |
| State S1 Fosc = 0.0055 RL = 4.1648 Angs E = 31077.60 cm^-1 lambda = 321.78 nm | | | | | | | | | | | | | | | | | | | | |
|  | | | | | | | | | | | | | | | | | | | | |
| HOMO-10->LUMO+3 | | | | | 82.63% | | | | | | | | | | | | |  | | |
| TOTAL | | | | | 82.63% | | | | | | | | | | | | |  | | |
|  | | | | | | | | | | | | | | | | | | | | |
| --------------------- | | | | | | | | | | | | | | | | | | | | |
| gamma(l) Parameters | | | | | | | | | | | | | | | | | | | | |
| --------------------- | | | | | | | | | | | | | | | | | | | | |
|  | | | | | | | | | | | | | | | | | | | | |
| State T1 | | | | | | | | | | | | | | | | | | | | |
| gamma(2) = 3.6482e+25 gamma(4) = 1.8138e+23 gamma(6) = 1.3717e+21 | | | | | | | | | | | | | | | | | | | | |
|  | | | | | | | | | | | | | | | | | | | | |
| State S1 | | | | | | | | | | | | | | | | | | | | |
| gamma(2) = 6.5316e+25 gamma(4) = 4.3451e+23 gamma(6) = 4.3971e+21 | | | | | | | | | | | | | | | | | | | | |
|  | | | | | | | | | | | | | | | | | | | | |
| ------------------------------------------------------ | | | | | | | | | | | | | | | | | | | | |
| Frank Condon's Factor (F) | | | | | | | | | | | | | | | | | | | | |
| ------------------------------------------------------ | | | | | | | | | | | | | | | | | | | | |
|  | | | | | | | | | | | | | | | | | | | | |
| F(T1 7F0->5D0) = 4.5132e+11 erg^-1 | | | | | | | | | | | | | | | | | | | | |
| F(T1 7F0->5D1) = 6.8457e+11 erg^-1 | | | | | | | | | | | | | | | | | | | | |
| F(T1 7F0->5D4) = 1.6509e+10 erg^-1 | | | | | | | | | | | | | | | | | | | | |
| F(T1 7F1->5D0) = 3.9205e+11 erg^-1 | | | | | | | | | | | | | | | | | | | | |
| F(T1 7F1->5D1) = 6.4720e+11 erg^-1 | | | | | | | | | | | | | | | | | | | | |
|  | | | | | | | | | | | | | | | | | | | | |
| F(S1 7F0->5D0) = 2.7955e+06 erg^-1 | | | | | | | | | | | | | | | | | | | | |
| F(S1 7F0->5D1) = 5.2870e+07 erg^-1 | | | | | | | | | | | | | | | | | | | | |
| F(S1 7F0->5D4) = 3.2691e+11 erg^-1 | | | | | | | | | | | | | | | | | | | | |
| F(S1 7F1->5D0) = 1.4133e+06 erg^-1 | | | | | | | | | | | | | | | | | | | | |
| F(S1 7F1->5D1) = 2.9090e+07 erg^-1 | | | | | | | | | | | | | | | | | | | | |
|  | | | | | | | | | | | | | | | | | | | | |
| ------------------------------------------------------ | | | | | | | | | | | | | | | | | | | | |
| Energy Transfer (Wet) and Back-Transfer Rates (Wbt) | | | | | | | | | | | | | | | | | | | | |
| Wet from the Multipolar Mechanism (WetMM) | | | | | | | | | | | | | | | | | | | | |
| Wet from the Exchange Mechanism (WetEX) | | | | | | | | | | | | | | | | | | | | |
| ------------------------------------------------------ | | | | | | | | | | | | | | | | | | | | |
|  | | | | | | | | | | | | | | | | | | | | |
| CHANNEL | | | | DELTA(cm^-1) | | | WetMM(s^-1) | | | | | WetEX(s^-1) | | | | Wet(s^-1) | | | Wbt(s^-1) | |
| T1 7F0->5D0 | | | | 2697.60 | | | 3.86e+00 | | | | | 0.00e+00 | | | | 3.86e+00 | | | 9.30e-06 | |
| T1 7F0->5D1 | | | | 963.60 | | | 0.00e+00 | | | | | 5.33e+07 | | | | 5.33e+07 | | | 5.24e+05 | |
| T1 7F0->5D4 | | | | -7595.40 | | | 1.44e-02 | | | | | 0.00e+00 | | | | 1.44e-02 | | | 9.54e+13 | |
| T1 7F1->5D0 | | | | 3069.60 | | | 0.00e+00 | | | | | 2.02e+08 | | | | 2.02e+08 | | | 8.18e+01 | |
| T1 7F1->5D1 | | | | 1335.60 | | | 2.98e+02 | | | | | 2.67e+04 | | | | 2.70e+04 | | | 4.47e+01 | |
|  | | | |  | | |  | | | | |  | | | |  | | |  | |
| S1 7F0->5D0 | | | | 13784.60 | | | 1.29e+01 | | | | | 0.00e+00 | | | | 1.29e+01 | | | 2.50e-28 | |
| S1 7F0->5D1 | | | | 12050.60 | | | 0.00e+00 | | | | | 9.17e+03 | | | | 9.17e+03 | | | 7.29e-22 | |
| S1 7F0->5D4 | | | | 3491.60 | | | 1.87e+05 | | | | | 0.00e+00 | | | | 1.87e+05 | | | 9.97e-03 | |
| S1 7F1->5D0 | | | | 14156.60 | | | 0.00e+00 | | | | | 1.62e+03 | | | | 1.62e+03 | | | 5.31e-27 | |
| S1 7F1->5D1 | | | | 12422.60 | | | 7.18e+03 | | | | | 2.68e+00 | | | | 7.19e+03 | | | 9.60e-23 | |
|  | | | | | | | | | | | | | | | | | | | | |
| ------------------------------------------------- | | | | | | | | | | | | | | | | | | | | |
| Theoretical Radiative emission rate (Arad) and | | | | | | | | | | | | | | | | | | | | |
| Branchio Rates (beta) | | | | | | | | | | | | | | | | | | | | |
| ------------------------------------------------- | | | | | | | | | | | | | | | | | | | | |
|  | | | | | | | | | | | | | | | | | | | | |
| Refractive Index Used = 1.424 | | | | | | | | | | | | | | | | | | | | |
|  | | | | | | | | | | | | | | | | | | | | |
| Theoretical Arad = 940.77 s^-1 | | | | | | | | | | | | | | | | | | | | |
|  | | | | | | | | | | | | | | | | | | | | |
| Beta values (contribution of each 5D0 -> 7FJ transition in percentage to radiative decay rate) | | | | | | | | | | | | | | | | | | | | |
|  | | | | | | | | | | | | | | | | | | | | |
| 5D0->7F1: 4.48 5D0->7F2: 81.61 5D0->7F3: 0.00 | | | | | | | | | | | | | | | | | | | | |
|  | | | | | | | | | | | | | | | | | | | | |
| 5D0->7F4: 13.83 5D0->7F5: 0.00 5D0->7F6: 0.09 | | | | | | | | | | | | | | | | | | | | |
|  | | | | | | | | | | | | | | | | | | | | |
| ----------------------------------------------------- | | | | | | | | | | | | | | | | | | | | |
| Theoretical Nonradiative Decay Rate (Anrad) and | | | | | | | | | | | | | | | | | | | | |
| Quantum Efficiency | | | | | | | | | | | | | | | | | | | | |
| ----------------------------------------------------- | | | | | | | | | | | | | | | | | | | | |
|  | | | | | | | | | | | | | | | | | | | | |
| Chosen Experimental Lifetime = 0.9269 ms | | | | | | | | | | | | | | | | | | | | |
|  | | | | | | | | | | | | | | | | | | | | |
| Theoretical Anrad = 138.10 s^-1 | | | | | | | | | | | | | | | | | | | | |
|  | | | | | | | | | | | | | | | | | | | | |
| Theoretical Quantum Efficiency = 87.20 % | | | | | | | | | | | | | | | | | | | | |
|  | | | | | | | | | | | | | | | | | | | | |
| ------------------------------------------------------------- | | | | | | | | | | | | | | | | | | | | |
| Rates Considered to Build the Rate Equations System | | | | | | | | | | | | | | | | | | | | |
| ------------------------------------------------------------- | | | | | | | | | | | | | | | | | | | | |
|  | | | | | | | | | | | | | | | | | | | | |
| Arad = 940.73 s^-1 | | | | | | | | | | | | | | | | | | | | |
| Anrad = 138.14 s^-1 | | | | | | | | | | | | | | | | | | | | |
| Emitting State: 5D0 | | | | | | | | | | | | | | | | | | | | |
| Absorbing State: S0 | | | | | | | | | | | | | | | | | | | | |
|  | | | | | | | | | | | | | | | | | | | | |
| Donor | Acceptor | | | | Rate(s^-1) | | | | | | | | | | | | | | | |
| S0 | S1 | | | | 1.0000e+04 | | | | | | | | | | | | | | | |
| S1 | T1 | | | | 1.0000e+08 | | | | | | | | | | | | | | | |
| T1 | S0 | | | | 1.0000e+05 | | | | | | | | | | | | | | | |
| S1 | S0 | | | | 1.0000e+06 | | | | | | | | | | | | | | | |
| 5D4 | 5D1 | | | | 1.0000e+06 | | | | | | | | | | | | | | | |
| 5D1 | 5D0 | | | | 1.0000e+06 | | | | | | | | | | | | | | | |
| T1 | 5D0 | | | | 5.1360e+08 | | | | | | | | | | | | | | | |
| 5D0 | T1 | | | | 9.3443e+01 | | | | | | | | | | | | | | | |
| T1 | 5D1 | | | | 1.4171e+08 | | | | | | | | | | | | | | | |
| 5D1 | T1 | | | | 6.2751e+05 | | | | | | | | | | | | | | | |
| T1 | 5D4 | | | | 4.5186e-02 | | | | | | | | | | | | | | | |
| 5D4 | T1 | | | | 1.3441e+14 | | | | | | | | | | | | | | | |
| S1 | 5D4 | | | | 1.7443e+05 | | | | | | | | | | | | | | | |
| 5D4 | S1 | | | | 3.7127e-03 | | | | | | | | | | | | | | | |
|  | | | | | | | | | | | | | | | | | | | | |
| --------------------------------------------------------------------- | | | | | | | | | | | | | | | | | | | | |
| Population of each State Involved in the Process of Energy Transfer | | | | | | | | | | | | | | | | | | | | |
| --------------------------------------------------------------------- | | | | | | | | | | | | | | | | | | | | |
|  | | | | | | | | | | | | | | | | | | | | |
| S0 population = 0.098257 | | | | | | | | | | | | | | | | | | | | |
| S1 population = 0.000010 | | | | | | | | | | | | | | | | | | | | |
| T1 population = 0.000002 | | | | | | | | | | | | | | | | | | | | |
| 5D4 population = 0.000000 | | | | | | | | | | | | | | | | | | | | |
| 5D1 population = 0.000153 | | | | | | | | | | | | | | | | | | | | |
| 5D0 population = 0.901578 | | | | | | | | | | | | | | | | | | | | |
|  | | | | | | | | | | | | | | | | | | | | |
| ----------------------------- | | | | | | | | | | | | | | | | | | | | |
| Quantum Yield: 86.32 % | | | | | | | | | | | | | | | | | | | | |
| ----------------------------- | | | | | | | | | | | | | | | | | | | | |
|  | | | | | | | | | | | | | | | | | | | | |
| Sensitization Efficiency = 98.99 % | | | | | | | | | | | | | | | | | | | | |
|  | | | | | | | | | | | | | | | | | | | | |
| --------------------------------- | | | | | | | | | | | | | | | | | | | | |
| Rates equations in LaTeX format | | | | | | | | | | | | | | | | | | | | |
| --------------------------------- | | | | | | | | | | | | | | | | | | | | |
|  | | | | | | | | | | | | | | | | | | | | |
| \[\frac{d{\eta_{S0}}}{dt} = -1.00\times{{10}^{4}} \eta_{S0}+1.00\times{{10}^{6}} \eta_{S1}+1.00\times{{10}^{5}} \eta_{T1}\] | | | | | | | | | | | | | | | | | | | | |
|  | | | | | | | | | | | | | | | | | | | | |
| \[\frac{d{\eta_{S1}}}{dt} = -(1.00\times{{10}^{6}} + 1.00\times{{10}^{8}} + 1.74\times{{10}^{5}}) \eta_{S1}+3.71\times{{10}^{-3}} \eta_{5D4}+1.00\times{{10}^{4}} \eta_{S0}\] | | | | | | | | | | | | | | | | | | | | |
|  | | | | | | | | | | | | | | | | | | | | |
| \[\frac{d{\eta_{T1}}}{dt} = -(1.00\times{{10}^{5}} + 4.52\times{{10}^{-2}} + 1.42\times{{10}^{8}} + 5.14\times{{10}^{8}}) \eta_{T1}+9.34\times{{10}^{1}} \eta_{5D0}+6.28\times{{10}^{5}} \eta_{5D1}+1.34\times{{10}^{14}} \eta_{5D4}+1.00\times{{10}^{8}} \eta_{S1}\] | | | | | | | | | | | | | | | | | | | | |
|  | | | | | | | | | | | | | | | | | | | | |
| \[\frac{d{\eta_{5D4}}}{dt} = -(3.71\times{{10}^{-3}} + 1.34\times{{10}^{14}} + 1.00\times{{10}^{6}}) \eta_{5D4}+1.74\times{{10}^{5}} \eta_{S1}+4.52\times{{10}^{-2}} \eta_{T1}\] | | | | | | | | | | | | | | | | | | | | |
|  | | | | | | | | | | | | | | | | | | | | |
| \[\frac{d{\eta_{5D1}}}{dt} = -(6.28\times{{10}^{5}} + 1.00\times{{10}^{6}}) \eta_{5D1}+1.00\times{{10}^{6}} \eta_{5D4}+1.42\times{{10}^{8}} \eta_{T1}\] | | | | | | | | | | | | | | | | | | | | |
|  | | | | | | | | | | | | | | | | | | | | |
| \[\frac{d{\eta_{5D0}}}{dt} = -9.34\times{{10}^{1}} \eta_{5D0}+1.00\times{{10}^{6}} \eta_{5D1}+5.14\times{{10}^{8}} \eta_{T1}\] | | | | | | | | | | | | | | | | | | | | |
|  | | | | | | | | | | | | | | | | | | | | |
|  | | | | | | | | | | | | | | | | | | | | |
| ------------------------------------------------------ | | | | | | | | | | | | | | | | | | | | |
| Required Citations for this Calculation | | | | | | | | | | | | | | | | | | | | |
| ------------------------------------------------------ | | | | | | | | | | | | | | | | | | | | |
|  | | | | | | | | | | | | | | | | | | | | |
| ORCA Program Reference | | | | | | | | | | | | | | | | | | | | |
|  | | | | | | | | | | | | | | | | | | | | |
| Neese F. | | | | | | | | | | | | | | | | | | | | |
| The ORCA program system. | | | | | | | | | | | | | | | | | | | | |
| Wiley Interdisciplinary Reviews-Computational Molecular Science, 2012, 2(1), 73-78. | | | | | | | | | | | | | | | | | | | | |
| http://dx.doi.org/10.1002/wcms.81 | | | | | | | | | | | | | | | | | | | | |
| doi:10.1002/wcms.81 | | | | | | | | | | | | | | | | | | | | |
|  | | | | | | | | | | | | | | | | | | | | |
|  | | | | | | | | | | | | | | | | | | | | |
| Revisited model in the Chapter 310 (Handbook) was used to calculate the energy transfer rates between the ligands and the lanthanide trivalent ion | | | | | | | | | | | | | | | | | | | | |
|  | | | | | | | | | | | | | | | | | | | | |
| Carneiro Neto, A. N.; Teotonio, E. E. S.; de Sa, G. F.; Brito, H. F.; Legendziewicz, J.; Carlos, L. D.; | | | | | | | | | | | | | | | | | | | | |
| Felinto, M. C. F. C.; Gawryszewskae, P.; Moura Jr, R. T.; Longo, R. L.; Faustino, W. M.; Malta, O. L. | | | | | | | | | | | | | | | | | | | | |
| Modeling intramolecular energy transfer in lanthanide chelates: | | | | | | | | | | | | | | | | | | | | |
| A critical review and recent advances. | | | | | | | | | | | | | | | | | | | | |
| Chapter 310 - Modeling intramolecular energy transfer in lanthanide chelates: A critical review and recent advances, | | | | | | | | | | | | | | | | | | | | |
| in Handbook on the Physics and Chemistry of Rare Earths, J.-C. G. Bunzli and V. K. Pecharsky, Editors. 2019, Elsevier. p. 55-162. | | | | | | | | | | | | | | | | | | | | |
| http://dx.doi.org/10.1016/bs.hpcre.2019.08.001 | | | | | | | | | | | | | | | | | | | | |
| doi:10.1016/bs.hpcre.2019.08.001 | | | | | | | | | | | | | | | | | | | | |
|  | | | | | | | | | | | | | | | | | | | | |
|  | | | | | | | | | | | | | | | | | | | | |
| Numerical solution for the rate equations and calculation of the emission quantum yield | | | | | | | | | | | | | | | | | | | | |
|  | | | | | | | | | | | | | | | | | | | | |
| Malta, O. L.; Brito, H. F.; Menezes, J. F. S.; Silva, F. R. G. E.; Donega, C. D.; Alves, S. | | | | | | | | | | | | | | | | | | | | |
| Experimental and theoretical emission quantum yield in the compound Eu(thenoyltrifluoroacetonate)(3).2(dibenzyl sulfoxide) | | | | | | | | | | | | | | | | | | | | |
| Chemical Physics Letter, 1998, 282(3-4), 233-238 | | | | | | | | | | | | | | | | | | | | |
| http://dx.doi.org/10.1016/S0009-2614(97)01283-9 | | | | | | | | | | | | | | | | | | | | |

**Figure 38**. LUMPAC output file for [Eu(btfa)_3_(bpy)] showing all calculated spectroscopic properties.

Theoretical Calculation of the Absorption Spectrum

**Figure 39** shows the module responsible for obtaining the theoretical absorption spectrum from the output file created by the ORCA and GAUSSIAN programs.


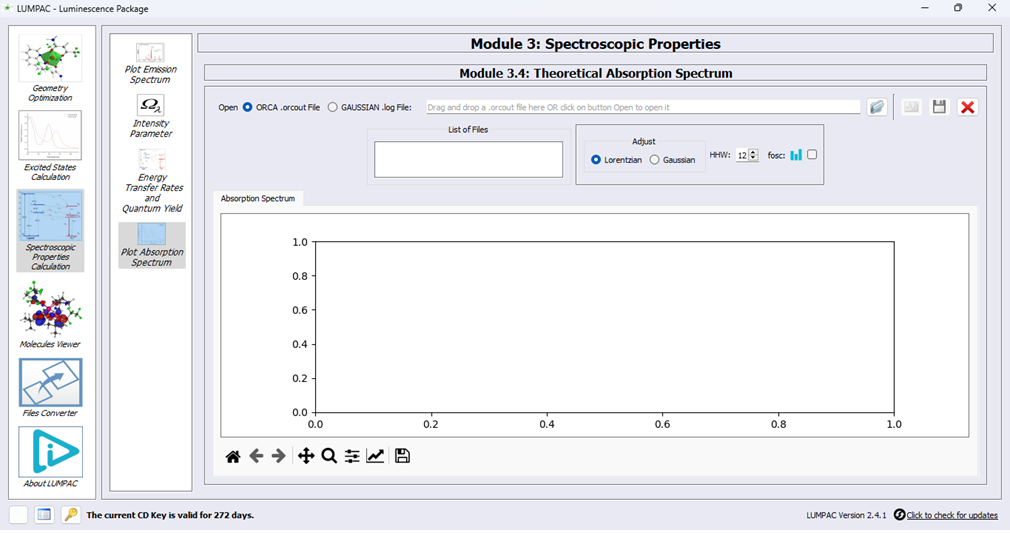


**Figure 39**. Module for generating the theoretical absorption spectrum from the ORCA and GAUSSIAN output file.

Procedure for Calculation of the Theoretical Absorption Spectrum using LUMPAC

1. *Click on button
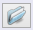
 (***Figure *40****) to open the ORCA output file (.orcout) and select the files listed in the List of Files for generating the spectrum.*

Like the previous module, LUMPAC 2.0 also applies DFT data calculated by ORCA and GAUSSIAN for generating the theoretical absorption spectra.


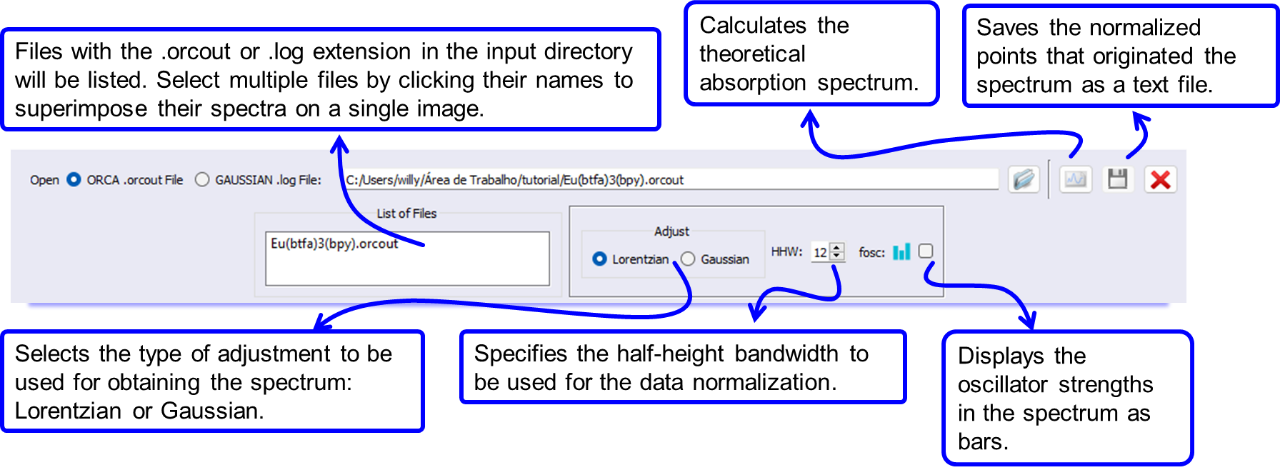


**Figure 40**. LUMPAC interface for generating the absorption spectrum from the ORCA and GAUSSIAN output file.

Electronic transitions from the singlet ground state to the singlet excited states are allowed, with their probability proportional to the oscillator strength of the transition (fosc), as shown in **Figure 41**. The theoretical absorption spectrum is generated using these oscillator strengths and excitation energies, applying an arbitrary full width at half maximum (FWHM). Both the FWHM and the wavelength range can be modified to match the experimental spectrum, if needed.

| . . . | | | | | | | | | |
| --- | --- | --- | --- | --- | --- | --- | --- | --- | --- |
| ------------------------------------------------------------------------------------------------------------------------ | | | | | | | | | |
| ABSORPTION SPECTRUM VIA TRANSITION ELECTRIC DIPOLE MOMENTS | | | | | | | | | |
| ------------------------------------------------------------------------------------------------------------------------ | | | | | | | | | |
| State | | | Energy | Wavelength | fosc | T2 | TX | TY | TZ |
|  | | | (cm-1) | (nm) |  | (au**2) | (au) | (au) | (au) |
| ------------------------------------------------------------------------------------------------------------------------ | | | | | | | | | |
| 1 | 31077.6 | | | 321.8 | 0.005489327 | 0.05815 | 0.20543 | -0.06040 | 0.11092 |
| 2 | 31269.4 | | | 319.8 | 0.001656443 | 0.01744 | 0.02418 | 0.12235 | 0.04342 |
| 3 | 31280.1 | | | 319.7 | 0.001575516 | 0.01658 | -0.03326 | 0.00598 | 0.12426 |
| . . . | | | | | | | | | |
| 48 | | 33217.7 | | 301.0 | spin forbidden (mult=3) | | | | |
| 49 | | 33259.4 | | 300.7 | spin forbidden (mult=3) | | | | |
| 50 | | 33950.4 | | 294.5 | spin forbidden (mult=3) | | | | |
| . . . | | | | | | | | | |

**Figure 41**. Singlet energies and oscillator strengths (singlet→singlet transitions) from the .orcout file, used to generate the theoretical absorption spectrum.

1. *Click on button
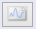
 to calculate the theoretical absorption spectrum, as shown in* **Figure *42****.*

**Figure 42** demonstrates the ability to overlay two spectra by selecting two different .orcout files. These files contain the excited states calculated for [Eu(btfa)_3_(bpy)] and a generic Eu^3+^ complex, both located in the calculation directory. A click on the bar representing the band intensity provides the molecular orbital composition of the most significant transitions.


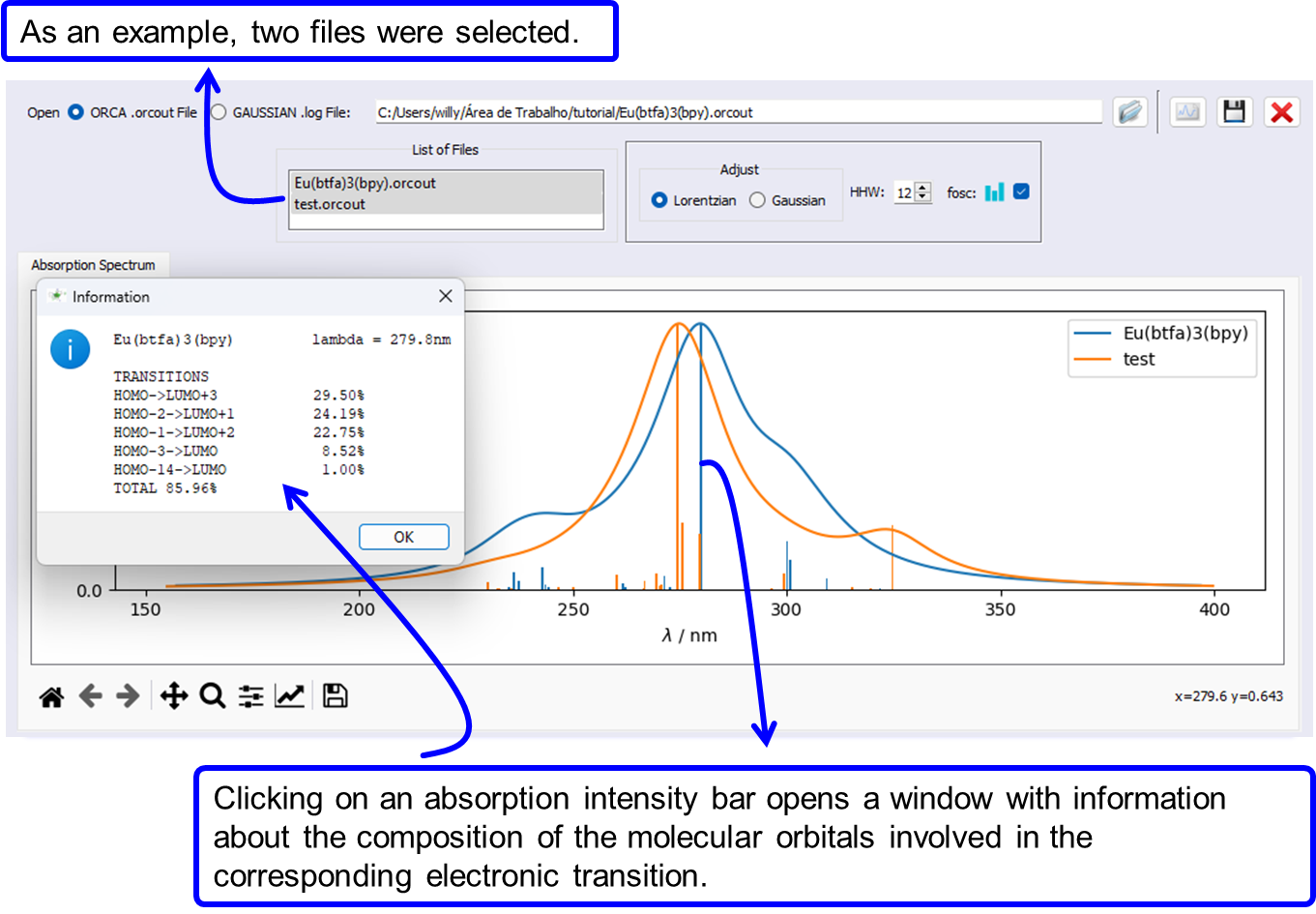


**Figure 42**. Absorption spectrum created by LUMPAC and options for visualizing excitation properties.

Module 4 – Molecule Viewer

LUMPAC 2.0 introduces a new molecule viewer module (**Figure 43**) for observing, editing, and saving images of the studied complex structures and calculated molecular orbitals.


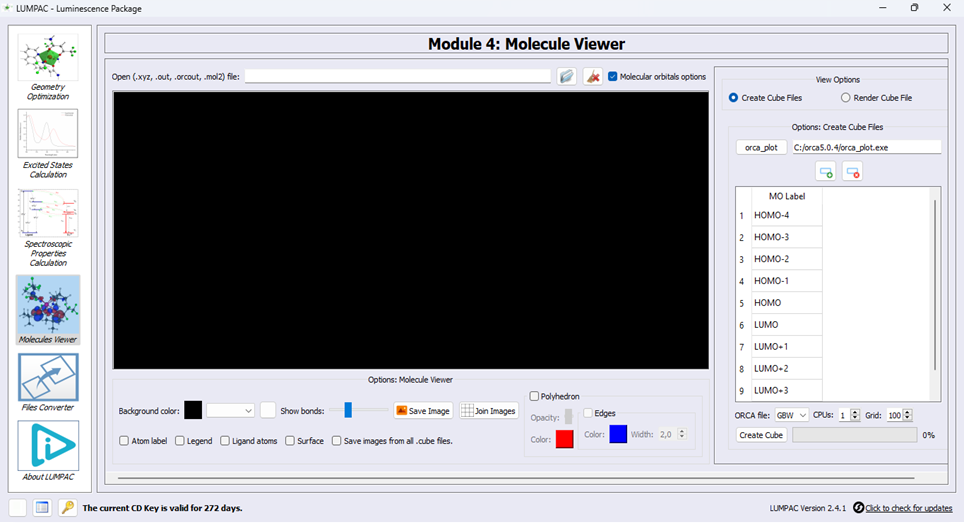


**Figure 43**. Module responsible for the molecule visualization.

1. *Click on button
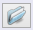
 (***Figure *44****) to open the desired input file.*

After opening the desired file, the structure will be displayed, as in Module 1 (**Figure 11**).


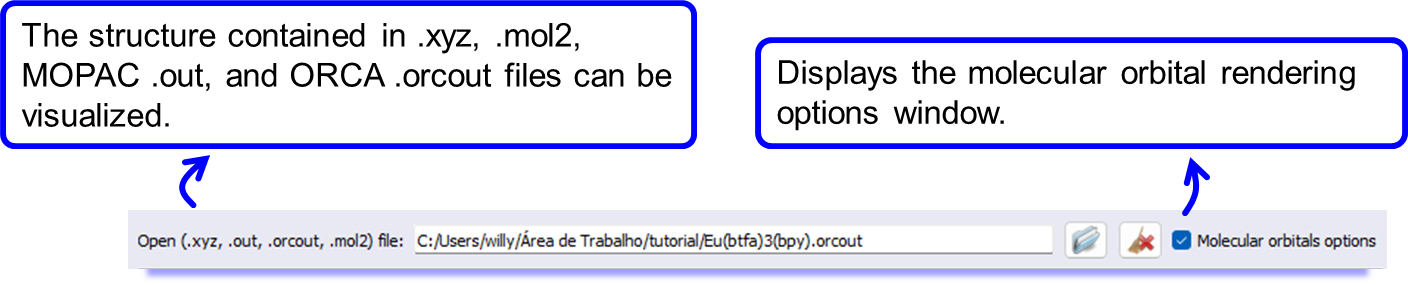


**Figure 44**. LUMPAC interface for opening the input file for structural visualization of the complex.

1. *To visualize the orbitals of the complex, select the options on the right side of the screen (***Figure *45****).*

LUMPAC 2.0 requires the utility orca_plot.exe program to visualize molecular orbitals (**Figure 45**). This program is distributed with ORCA and is in the same directory as orca.exe. The orca_plot.exe program creates .cube files for the selected orbitals, which will be saved in the directory containing the files with orbital information (.gbw, .nto, or .loc) created during the excited states calculations. **Figure 45** illustrates how to select molecular orbitals for visualization.


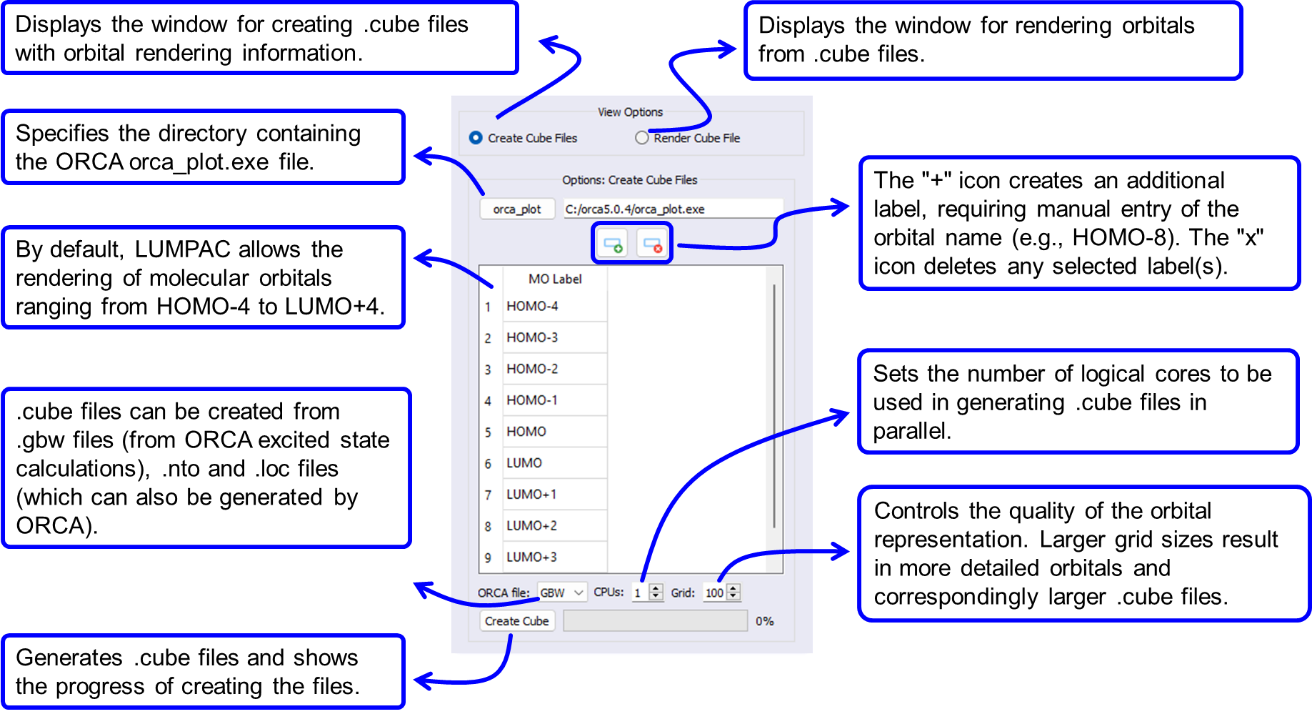


**Figure 45**. Interface with options for generating molecular orbitals.

1. *Select the orbitals for rendering, following the instructions in* **Figure *46****.*


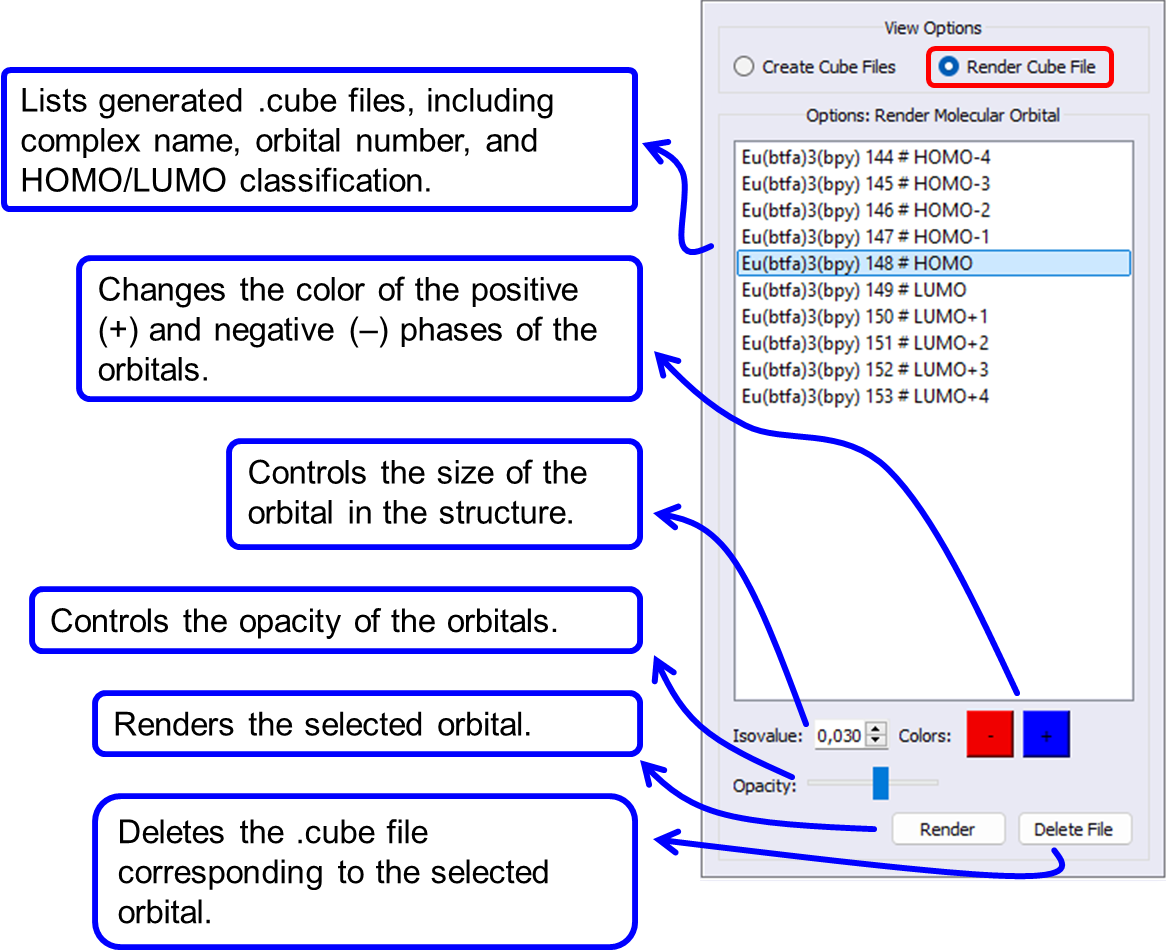


**Figure 46**. Interface for selecting the molecular orbitals to be rendered.

1. *Once rendered, the orbitals will be displayed superimposed on the molecule (***Figure *47****).*


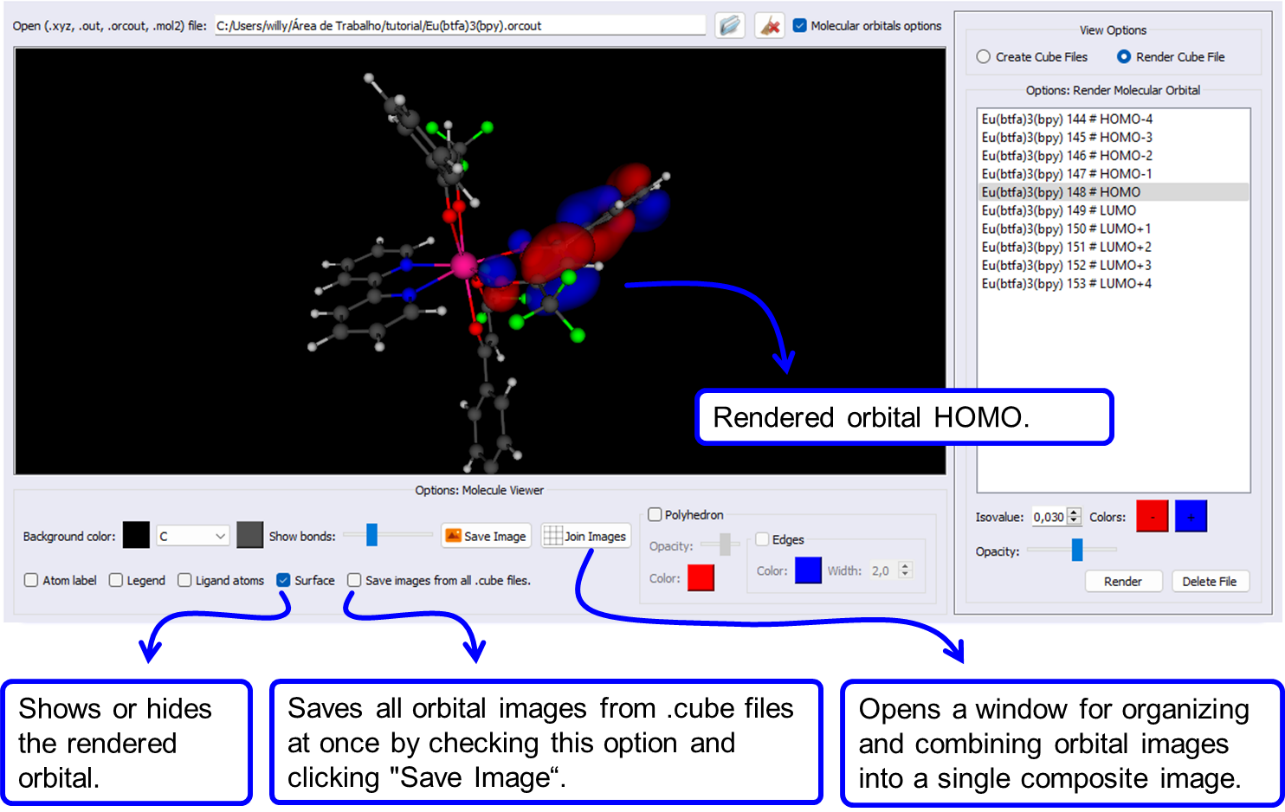


**Figure 47**. Visualization of the rendered molecular orbital in the molecule viewer.

LUMPAC 2.0 includes a feature called “Join Images” (**Figure 47**) for organizing and labeling the molecular orbitals into a single image, and the available options are displayed in **Figure 48**. The individual orbital images are saved as .png format, while the combined image is saved as new_image.jpg.


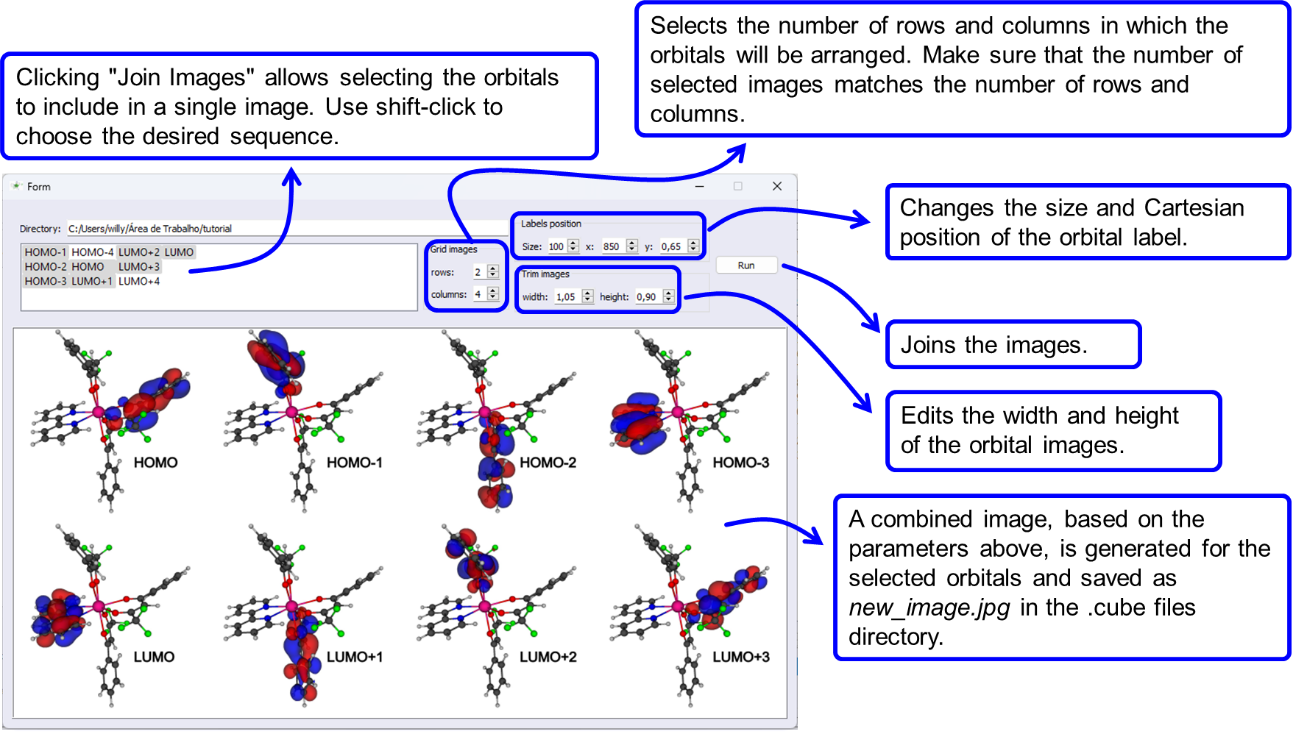


**Figure 48**. “Join Images” window with options for combining the orbital images.

Module 5 – File Converter

**Figure 49** illustrates the LUMPAC module for file conversion. The conversion can be done for a single or multiple files within a directory. **Table 2** lists the available conversions and their descriptions.


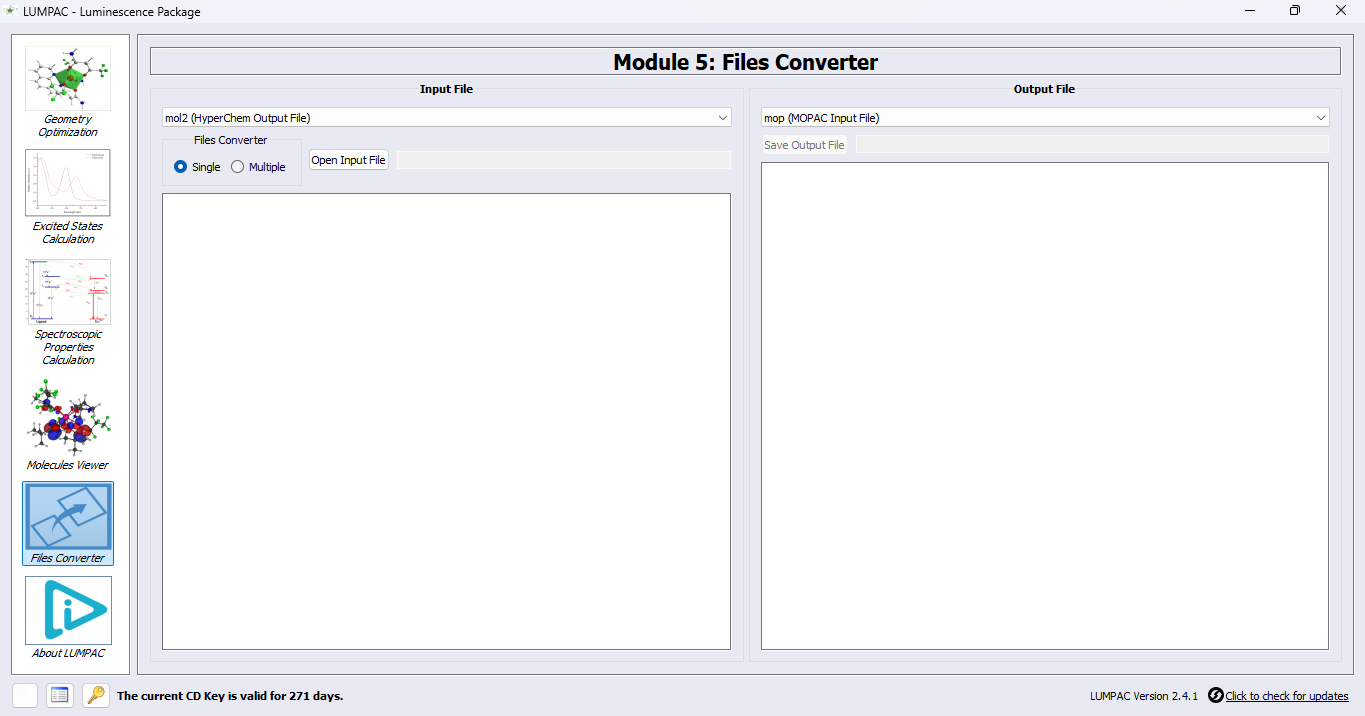


**Figure 49**. Module responsible for file conversion.

| **Table 2**. File conversions that LUMPAC can execute. | | |
| --- | --- | --- |
| **Input files** | **Output files** | **Description** |
| **.mol2** | **.mop**  *Input file for MOPAC* | From the .mol2 file, a MOPAC input file is generated with the RM1 model as the default. Thus, geometry optimization can be performed externally using the generated .mop file. |
|  | **.orcinp**  *Input file for ORCA* | From the .mol2 file, an ORCA input file is generated for semiempirical INDO/S-CIS, DFT, and TD-DFT calculations (**Figure 50**). This enables users to perform DFT geometry optimizations and excited state calculations externally to LUMPAC. |
| **.log**  *Gaussian output file* | **.spec**  *File with spectroscopic properties.* | Extracts spectroscopic properties from the GAUSSIAN output files. |
|  | **.txt**  *File with theoretical absorption spectrum.* | From the GAUSSIAN output file, the theoretical absorption spectrum is generated, providing options to set the full width at half maximum (FWHM) and the minimum and maximum wavelength of the resulting spectrum. |
| **.out**  *MOPAC output file.* | **.gjf**  *Input file for GAUSSIAN.* | From the geometry optimized with MOPAC, a GAUSSIAN input file is generated for DFT calculations using the B3LYP method. |
|  | **.mop**  *Input file for MOPAC.* | Using the MOPAC output file (.out), a MOPAC input file is generated. |
|  | **.orcinp**  *Input file for ORCA.* | From the MOPAC output file, an ORCA input file is generated for semiempirical INDO/S-CIS, DFT, and TD-DFT calculations (**Figure 50**). |
|  | **.sph**  *File with spherical coordinates.* | The Cartesian coordinates optimized by MOPAC are converted to spherical coordinates, using the Ln^3+^ ion listed at the top as the reference point. |
|  | **.zmt**  *File with internal coordinates.* | The Cartesian coordinates optimized by MOPAC are converted to internal coordinates for visualization in other programs. |
| **.orcout**  *ORCA output file.* | **.gjf**  *Input file for GAUSSIAN.* | From the optimized geometry, a GAUSSIAN input file is generated for DFT calculations using the B3LYP method. |
|  | **.mop**  *Input file for MOPAC.* | Using the ORCA output file (.orcout), whether semiempirical or DFT, a MOPAC input file is generated. |
|  | **.orcinp**  *Input file for ORCA.* | From the ORCA output file, another ORCA input file is generated for semiempirical with INDO/S-CIS, DFT, and TD-DFT calculations (**Figure 50**). |
|  | **.spec**  *File with spectroscopic properties.* | The excited states calculated by ORCA are extracted, and the corresponding *R_L_* parameters are determined. |
|  | **.sph**  *File with spherical coordinates.* | The Cartesian coordinates optimized in the ORCA output file are converted to spherical coordinates, using the Ln^3+^ ion listed at the top as the reference point. |
|  | **.txt**  *File with theoretical absorption spectrum.* | From the GAUSSIAN output file, the theoretical absorption spectrum is generated, providing options to set the full width at half maximum (FWHM) and the minimum and maximum wavelength of the resulting spectrum. |
|  | **.zmt**  *File with internal coordinates.* | The Cartesian coordinates optimized by MOPAC are converted to internal coordinates for visualization in other programs. |

Among the ORCA input file options (**Figure 50**), INDO/S-CIS calculations can be selected considering the default keywords used in Module 2. By selecting the DFT option, an input file for geometry optimization is created with default PBE1PBE/TZVP settings. The TDDFT option creates an input file for calculating singlet and triplet excited states using the hybrid functional CAM-B3LYP with the TZVP basis set, calculating 25 transitions (or roots, “nroots 25”) and generating the natural transition orbital files (“donto true”). All keywords in **Figure 50** can be edited directly, allowing users to customize the calculation parameters. For any DFT calculation (geometry optimization or excited state) with Eu^3+^, a database of ECPs can be selected by clicking on the ECP option.


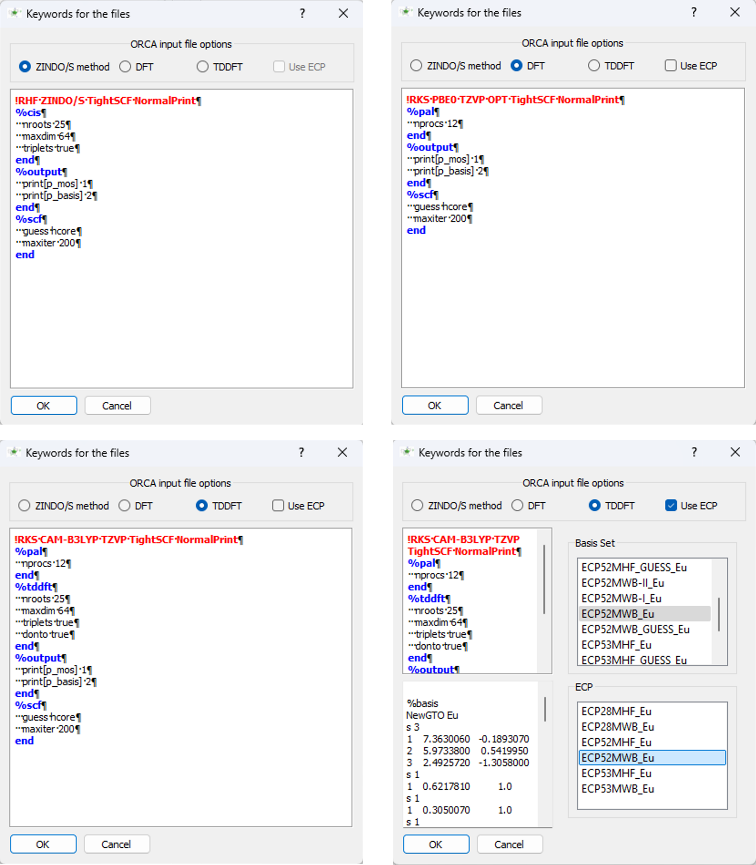


**Figure 50**. Input file options for ORCA after selecting the output file for .orcinp (ORCA Input File) and clicking on Save Output File.

Module 6 – About LUMPAC

This is not strictly a module, but rather information about the program and the team responsible for developing LUMPAC 2.0 (**Figure 51**).


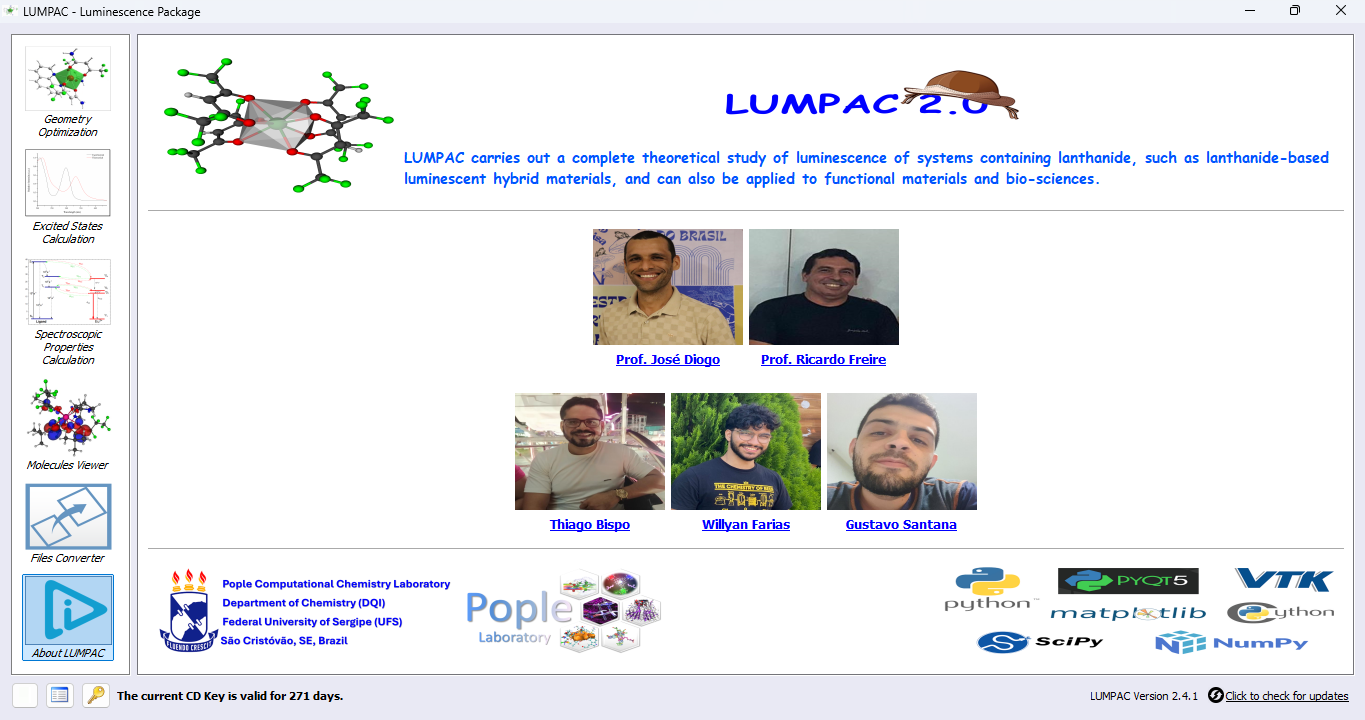


**Figure 51**. The last LUMPAC module, containing information about the developers, the institution, and the libraries used to develop LUMPAC 2.0.

**Referencies**

1. M. A. M. Filho, J. D. L. Dutra, H. L. B. Cavalcanti, G. B. Rocha, A. M. Simas, R. O. Freire, *J. Chem. Theory Comput.*, **2014**, *10*, 3031–3037.

2. M. A. M. Filho, J. D. L. Dutra, G. B. Rocha, R. O. Freire, A. M. Simas, *RSC Adv.*, **2013**, *3*, 16747–16755.

3. N. B. Da Costa, R. O. Freire, G. B. Rocha, A. M. Simas, *Inorg. Chem. Commun.*, **2005**, *8*, 831–835.

4. R. O. Freire, G. B. Rocha, A. M. Simas, *J. Braz. Chem. Soc.*, **2009**, *20*, 1638–1645.

5. R. O. Freire, A. M. Simas, *J. Chem. Theory Comput.*, **2010**, *6*, 2019–2023.

6. J. D. L. Dutra, M. A. M. Filho, G. B. Rocha, R. O. Freire, A. M. Simas, J. J. P. Stewart, *J. Chem. Theory Comput.*, **2013**, *9*, 3333–3341.

7. I. J. Al-Busaidi, R. Ilmi, D. Zhang, J. D. L. Dutra, W. F. Oliveira, N. K. Al Rasbi, L. Zhou, W. Y. Wong, P. R. Raithby, M. S. Khan, *Dye. Pigment.*, **2022**, *197*.

8. O. Ivanciuc, *J. Chem. Inf. Comput. Sci.*, **1996**, *36*, 612–614.

9. A. Allouche, *J. Comput. Chem.*, **2012**, *32*, 174–182.

10. M. D. Hanwell, D. E. Curtis, D. C. Lonie, T. Vandermeerschd, E. Zurek, G. R. Hutchison, *J. Cheminform.*, **2012**, *4*.

11. C. F. MacRae, I. Sovago, S. J. Cottrell, P. T. A. Galek, P. McCabe, E. Pidcock, M. Platings, G. P. Shields, J. S. Stevens, M. Towler, P. A. Wood, *J. Appl. Crystallogr.*, **2020**, *53*, 226–235.

12. J. E. Moussa, J. J. P. Stewart, MOPAC. 2024.

13. F. Neese, Software update: The ORCA program system—Version 5.0, *Wiley Interdisciplinary Reviews: Computational Molecular Science*, *12*. 2022.

14. J. Ridley, M. Zerner, *Theor. Chim. Acta*, **1973**, *32*, 111–134.

15. J. E. Ridley, M. C. Zerner, *Theor. Chim. Acta*, **1976**, *42*, 223–236.

16. J. D. L. Dutra, N. B. D. Lima, R. O. Freire, A. M. Simas, *Sci. Rep.*, **2015**, *5*, 1–12.

17. A. Ćirić, Ł. Marciniak, M. D. Dramićanin, *Sci. Rep.*, **2022**, *12*, 1–10.
